# Post-Marketing Safety of mRNA Vaccines: A Real-World Study Integrating Literature Case Reports and Vaccine Adverse Event Reporting System

**DOI:** 10.3390/vaccines14060524

**Published:** 2026-06-12

**Authors:** Bagejiang Tulisibaike, Tian-Yi Yang, Wen-Jun Gu, Huan Liu, Yuan-Hui Wang, Jin-Qi Yang, Tong Wang, Si-Miao Ding, Rong-Xue Cai, Yuan-Jie Wang, Wei Wang, Hong-Xing Pan, Fang Shao, Yu-Wen Su

**Affiliations:** 1 National Vaccine Innovation Platform, School of Pharmacy, Nanjing Medical University, Nanjing 211166, China; 2 Institute of Pharmacovigilance, Nanjing Medical University, Nanjing 211166, China; 3 Department of Surgery, Mount Sinai West Hospital, New York, NY 10019, USA; 4 Department of Clinical Pharmacology, Sir Run Run Hospital, Nanjing Medical University, Nanjing 211166, China; 5 Jiangsu Provincial Centre for Disease Control and Prevention, Public Health Research Institute of Jiangsu Province, Nanjing 210009, China; 6 National Vaccine Innovation Platform, School of Public Health, Nanjing Medical University, Nanjing 211166, China

**Keywords:** mRNA vaccine, VAERS, adverse event following immunizations, real-world study

## Abstract

**Background**: mRNA vaccines, first approved in December 2020, have been used globally to prevent infectious diseases, and those for treating cancers are being developed. Safety-related labelling changes of Comirnaty and Spikevax were made in June 2025; however, concerns remain. This study assessed the potential risks associated with mRNA vaccines on the indications previously approved, utilizing Real-World Data (RWD) of Adverse Events Following Immunization (AEFIs) derived from the Vaccine Adverse Event Reporting System (VAERS) and Academic Literature Databases (ALD). **Methods**: A Disproportionality Analysis (DPA) was performed using the Reporting Odds Ratio (ROR) and the Bayesian Confidence Propagation Neural Network (BCPNN) algorithm on spontaneous case reports from VAERS. Statistical positive signals were cross-validated with literature case reports from ALD to provide more comprehensive medical descriptions and clearer causal assessments, and compared with safety information documented in clinical trials and on vaccine labelling. Time-to-onset, stratified, and immunization schedule analyses were conducted to characterize the safety profiles of mRNA vaccines. **Results**: In total, 5,040,725 spontaneous case reports and 4,387 literature case reports were analyzed. In both VAERS and ALD, new signals involving blood and lymphatic system disorders (e.g., thrombotic thrombocytopenic purpura) and ear and labyrinth disorders (e.g., deafness) were detected from Comirnaty as Designated Medical Events (DMEs), while blood and lymphatic system disorders (e.g., thrombotic thrombocytopenic purpura) from Spikevax in ALD only. No new signals were detected from other vaccines on the DMEs list. In VAERS, Serious Adverse Events (SAEs) were more common in females, while death risk was higher in males. In ALD, SAEs were more common in males for most mRNA vaccines, except Comirnaty. Medical history emerged as a key risk factor for SAEs, particularly among older adults. **Conclusions**: Statistically significant safety signals were detected across all mRNA vaccines based on five-year cumulative RWD, indicating the need of intensified monitoring of specific populations, including older adults and individuals with medical histories, alongside further optimization of vaccination strategies.

## 1. Introduction

Vaccination is the most effective strategy for preventing and controlling the transmission of infectious diseases [1,2]. The mRNA vaccines, first approved in December 2020, have demonstrated favourable safety and efficacy [3,4,5,6], significantly curbing the prevention and control of COVID-19 and lower respiratory tract infections caused by Respiratory Syncytial Virus (RSV) [7]. However, the long-term safety of these products still requires wider evaluation. In 2021, studies identified an increasing number of myocarditis and pericarditis reports associated with Comirnaty and Spikevax, particularly among the adolescents and young adult males [8]. Meanwhile, the warnings and precautions section for both vaccines was updated in July 2025 [9].

The Vaccine Adverse Event Reporting System (VAERS) is a national surveillance system jointly operated by the U.S. Food and Drug Administration (FDA) and the U.S. Centres for Disease Control and Prevention (CDC) to monitor the safety of all authorized vaccines in the United States [10,11]. Disproportionality Analysis (DPA) is the most widely used tool for signal detection in pharmacovigilance [12].

In this study, the safety profiles of all authorized mRNA vaccines (Comirnaty, Spikevax, mRESVIA, MNEXSPIKE, Comirnaty Bivalent, Spikevax Bivalent) were evaluated. The AEFIs from VAERS were analyzed using the methods of Reporting Odds Ratio (ROR) and Bayesian Confidence Propagation Neural Network (BCPNN) through DPA. These AEFIs were further cross-validated with the signals through literature case reports from Academic Literature Databases (ALD), including PubMed, Embase, Web of Science, and Scopus, which provide more comprehensive medical descriptions and clearer causal assessments. Signals were compared with the safety information of clinical trials and product labelling. The statistically positive signals identified in this study are expected to guide the risk management and rational use of mRNA vaccines.

## 2. Materials and Methods

### 2.1. Data Source and Study Design

**Figure 1:**
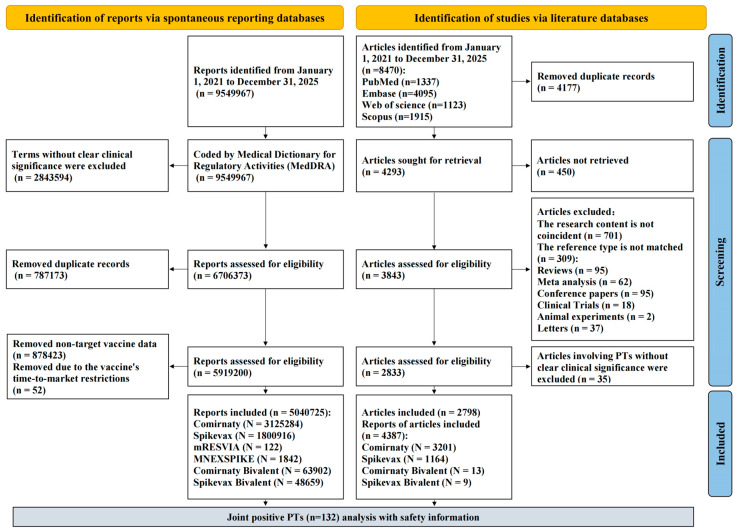
Flowchart of data extraction from the VAERS and Literature case reports.

This Real-World Study (RWS) was designed as a safety signal detection and evidence integration study of vaccine pharmacovigilance. DPA was employed to detect the safety signals of mRNA vaccines (see Table S1) in VAERS by using both ROR and BCPNN methods [13], while literature case reports and clinical trial publications were retrieved from ALD. The datasets used in this study were obtained from publicly accessible sources, including VAERS and ALD. All data analyses were performed using R software (version 4.5.2), and the study flowchart is presented in Figure 1.

DPA was conducted in accordance with the guidelines of the REporting of A Disproportionality analysis for drUg Safety signal detection using spontaneous case reports in PharmacoVigilance (READUS-PV) [14]. Literature case reports were analyzed according to the Preferred Reporting Items for Systematic Reviews and Meta-Analyses (PRISMA) 2020 guidelines [15]. The PICOS (Population, Intervention, Comparison, Outcomes, Study) framework was adopted to define the study scope (see Table S2) [16].

Safety signals were cross-validated by integrating DPA results based on VAERS and literature case reports from ALD and compared with safety information on the FDA-approved mRNA vaccine labelling. New safety signals, defined as those with unconfirmed causality and not listed in the labelling, were thereby identified. As Comirnaty Bivalent and Spikevax Bivalent were withdrawn [17], the safety signals of both vaccines were not compared with their labelling in the present study.

### 2.2. Data Description, Access, and Preprocessing

In this study, all AEFI-related medical events were coded by Preferred Terms (PTs) in the Medical Dictionary for Regulatory Activities (MedDRA) version 28.1, and these terms were further standardized into System Organ Classes (SOCs) [10]. We excluded certain terms without clear clinical significance (see Table S3).

### 2.3. Variables Definition

Serious Adverse Events (SAEs) were classified as died (DIED), life-threatening (L_THREAT), hospitalized (HOSPITAL) or prolonged hospitalization (X_STAY), disability (DISABLE), and Congenital anomaly or birth defect (BIRTH_DEFECT) [10,11]. The proportion of SAEs among all AEFIs during primary or booster immunization was calculated as follows: $\mathrm{proportion}\;\mathrm{of}\;\mathrm{SAE}\;(\%)\;=\;\frac{\mathrm{Number}\;\mathrm{of}\;\mathrm{SAE}\;\mathrm{cases}}{\mathrm{Total}\;\mathrm{Number}\;\mathrm{of}\;\mathrm{AEFI}\;\mathrm{cases}}\;\times \;100\%$.

To determine the clinical importance of AEFIs, we adopted the list of Designated Medical Events (DMEs) established by the European Medicines Agency (EMA), which serves as a standardized criterion for assessing the importance of medical events in routine pharmacovigilance activities [18].

### 2.4. DPA

The ROR and BCPNN methods were applied using a 2 × 2 contingency table (see Table S4) [19,20]. The corresponding formulas, detection thresholds, and confidence/credible intervals (CIs) are provided in Table S5; other vaccines refer to all vaccines in the VAERS except the target vaccine. Statistical significance was determined as follows: for the ROR, a signal was considered if the number of AEFI reports (a) was ≥3 and the lower limit of the 95% CI (ROR_025_) was >1. For the BCPNN, a signal was considered if the lower limit of the 95% CI (IC_025_) was >0. Both ROR and BCPNN were used to minimize potential bias originating from the use of a single algorithm [13]. A signal was defined as positive if it exceeded the threshold in both algorithms. A higher value of ROR_025_ or IC_025_ indicated a stronger association between the target vaccine and the AEFI.

### 2.5. Analysis of Literature Case Reports

Based on literature case reports in ALD, mRNA vaccine-related AEFIs were identified using search terms listed in Table S6. Core fields of AEFIs were extracted individually from more detailed medical descriptions, including age, sex, medical history, vaccine product name, immunization dose and procedure, diagnosis terms, seriousness, onset and duration time. The terms to make a diagnosis were coded using PT in MedDRA, and the seriousness of AEFIs was classified in accordance with the international standard. A signal was defined as positive if the corresponding PT was reported in ≥3 cases.

## 3. Results

### 3.1. AEFIs in the VAERS

#### 3.1.1. Description

A total of 5,040,725 AEFI reports were retrieved from VAERS, including 3,125,284 for Comirnaty, 1,800,916 for Spikevax, 122 for mRESVIA, 1842 for MNEXSPIKE, 63,902 for Comirnaty Bivalent, and 48,659 for Spikevax Bivalent (Table 1). Data for all mRNA vaccines were aggregated, involving a population with a median age of 50 years. AEFIs were more common in females (69.44%), a trend observed across all vaccines. For all mRNA vaccines, the proportion of vaccinees with a medical history ranged from 26.46% to 70.49%.

#### 3.1.2. AEFI Distributions at the PT and SOC Levels

Top-ranked Positive PTs listed in Table S7 were identified by the top 10 ranking criteria (sorted by either report count or DPA results, i.e., ROR_025_ or IC_025_), or selected from PTs with positive signal included in the DMEs list. The DPA results for all positive PTs are presented in Table S8.

A total of 27 positive PTs of all mRNA vaccines were obtained across 12 SOCs, with 2 on the DMEs list: anaphylactic reaction and acute kidney injury. Comirnaty generated 45 positive PTs across 19 SOC, with 18 on the DMEs list, such as anaphylactic reaction, angioedema, and deafness. Compared with Comirnaty’s labelling (August 2025) [21], new signals were detected, including some on the DMEs list (e.g., deafness, blindness, sudden hearing loss, anaphylactic shock, renal failure, rhabdomyolysis, pancreatitis acute, pancreatitis, autoimmune hepatitis, optic ischaemic neuropathy, autoimmune haemolytic anemia, thrombotic thrombocytopenic purpura, haemolysis, sudden cardiac death, and dermatitis exfoliative generalized), and other positive PTs (e.g., dysmenorrhoea, menstruation delayed, disease recurrence, toxoplasmosis, and marasmus). Toxoplasmosis is an infectious disease and pathogenically unrelated to the vaccine [22]. Spikevax generated 20 positive PTs across 6 SOC, with 1 on the DMEs list (erythema multiforme). Compared with Spikevax’s labelling (August 2025) [23], new signals included sedation and pain in the extremity. A total of 4positive PTs across 3SOCs were generated for mRESVIA, with none on the DMEs list. Compared with mRESVIA’s labelling (June 2025) [24], a new signal appeared as feeling abnormal. MNEXSPIKE generated 20 positive PTs across 6SOCs, with none on the DMEs list. Compared with MNEXSPIKE’s labelling (August 2025) [25], new signals included pain in an extremity, loss of consciousness, urticaria, hyperhidrosis, and gait disturbance. Comirnaty Bivalent showed 23 positive PTs across 8 SOCs, with 4 on the DMEs list: acute kidney injury, pulmonary hypertension, rhabdomyolysis, and febrile neutropenia. Spikevax Bivalent had 19 positive PTs across 4 SOCs, with 1 on the DMEs list (pulmonary fibrosis).

Monovalent mRNA vaccines (Comirnaty, Spikevax, mRESVIA, and MNEXSPIKE) generated 26 positive PTs across 11 SOCs, with 2 on the DMEs list: anaphylactic reaction and sudden hearing loss. Bivalent mRNA vaccines (Comirnaty Bivalent and Spikevax Bivalent) generated 23 positive PTs across 5 SOC, with 3 on the DMEs list: acute kidney injury, pulmonary hypertension, and pulmonary fibrosis.

#### 3.1.3. Time-to-Onset (TTO) Analysis

**Figure 2:**
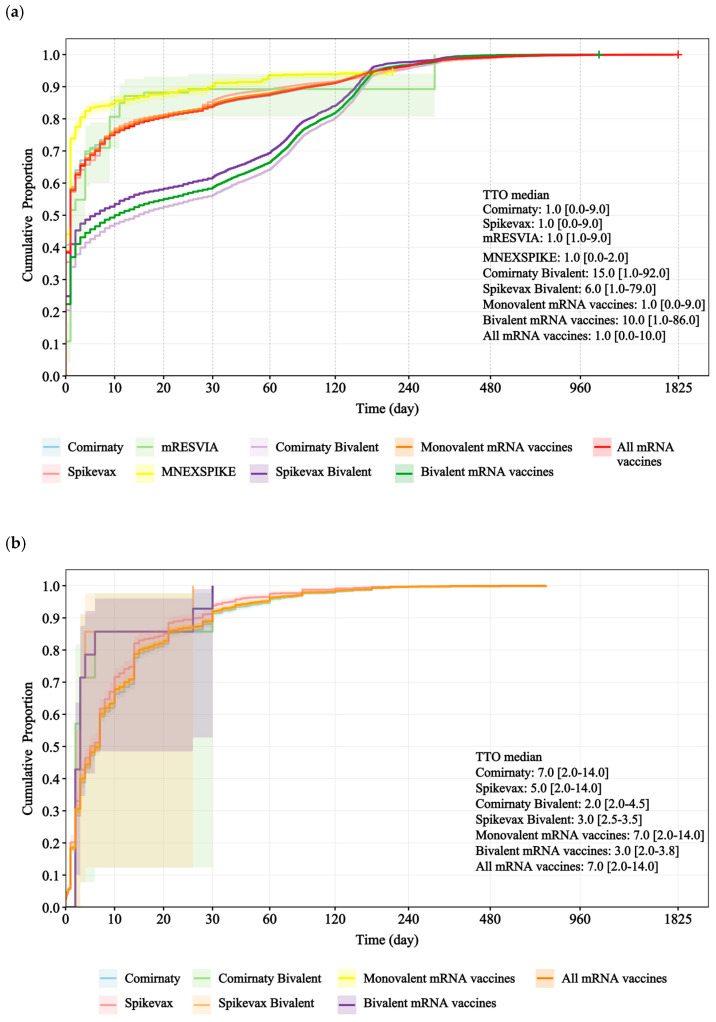

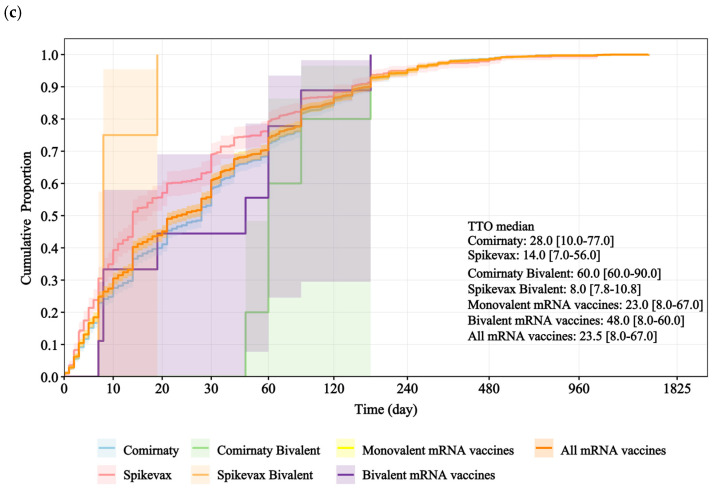
The TTO or Duration of AEFIs in VAERS and ALD. (**a**) The TTO of AEFIs in VAERS; (**b**) Intervals from vaccination to AEFIs onset in the literature case reports; (**c**) Duration of AEFIs in the literature case reports.

The TTO analysis showed AEFI intervals using median time and interquartile ranges (IQRs) (Figure 2a). For all mRNA vaccines, AEFIs occurred shortly after vaccination. The earliest occurrences were observed in the use of Comirnaty (median = 1.0 day, IQR: 0.0–9.0 days), Spikevax (median = 1.0 day, IQR: 0.0–9.0 days), mRESVIA (median = 1.0 day, IQR: 1.0–9.0 days), and MNEXSPIKE (median = 1.0 day, IQR: 0.0–2.0 days), followed by Spikevax Bivalent (median = 6.0 days, IQR: 1.0–79.0 days), and Comirnaty Bivalent (median = 15.0 days, IQR: 1.0–92.0 days). AEFIs occurred earlier in the use of monovalent mRNA vaccines (median = 1.0 day, IQR: 0.0–9.0 days) than bivalent mRNA vaccines (median = 10.0 days, IQR: 1.0–86.0 days).

#### 3.1.4. SAEs Analysis

The median age of individuals with SAEs (see Table S9) was generally older than that with AEFIs (Table 1), indicating a higher risk of SAEs in older adults. Most SAEs were more frequent in females, but more males had DIED. Meanwhile, an older median age was linked to a greater proportion of vaccinees with medical histories, reflecting a cumulative risk for SAEs. Additionally, multiple SAEs were related to general disorders and administration site conditions (e.g., fatigue) and respiratory, thoracic and mediastinal disorders (e.g., dyspnoea) (see Table S10).

#### 3.1.5. Immunization Schedule Analysis

SAEs during primary and booster immunization were analyzed (see Table S11). For all mRNA vaccines and monovalent mRNA vaccines, the number of SAEs was larger in primary immunization, but the proportion was higher in booster immunization. However, for bivalent mRNA vaccines, the number of SAEs was larger in booster immunization, but the proportion was higher in primary immunization. The highest incidence was in primary immunization with Comirnaty Bivalent (48.04%), and the lowest was in booster immunization with Spikevax Bivalent (15.13%). Other SAEs had incidences around 20%. PTs leading to SAEs, including pyrexia, dyspnoea, cardiac arrest, cerebrovascular accident, pulmonary embolism, and myalgia, shared a similar distribution (see Table S12).

### 3.2. AEFIs in ALD

#### 3.2.1. Description

A total of 4387 literature case reports were identified (Table 1), involving a population (46.00% male and 52.47% female) with a median age of 48 years. Monovalent mRNA vaccines had 4370 reports, involving a population (52.45% females) with a median age of 48 years. Among these, Comirnaty appeared in 3204 reports (median age 47 years, 52.72% female), and Spikevax in 1166 reports (median age 49 years, 51.72% female). No reports about mRESVIA and MNEXSPIKE were found. Bivalent mRNA vaccines appeared in 17 reports: 10 about Comirnaty Bivalent, 7 about Spikevax Bivalent.

#### 3.2.2. AEFI Distributions at the PT and SOC Levels

PTs reported in ≥3 cases were considered as PTs with positive signals (positive PTs) in ALD analysis. Top-ranked positive PTs in Table S13 were identified using the top 10 ranking criteria (sorted by either the number of corresponding events in the literature case reports or VAERS-derived DPA results, i.e., ROR_025_ or IC_025_), or selected from PTs with positive signals included in the DMEs list.

A total of 38 positive PTs of all mRNA vaccines were obtained across 15 SOCs, with 20 on the DMEs list, such as immune thrombocytopenia, thrombotic thrombocytopenic purpura, and autoimmune hepatitis. Comirnaty generated 32 positive PTs across 14 SOCs, with 17 on the DMEs list, such as immune thrombocytopenia, thrombotic thrombocytopenic purpura, and autoimmune hepatitis. Compared with Comirnaty’s labelling (August 2025) [21], new signals were detected, including some on the DMEs list (e.g., immune thrombocytopenia, thrombotic thrombocytopenic purpura, autoimmune hepatitis, rhabdomyolysis, optic ischaemic neuropathy, pancreatitis acute, autoimmune haemolytic anemia, acute kidney injury, deafness, aplastic anemia, pulmonary hypertension, stevens–johnson syndrome, deafness neurosensory, and toxic epidermal necrolysis), and other positive PTs (e.g., guillain-barre syndrome, herpes zoster, rheumatic disorder, sarcoidosis, and polyneuropath). Spikevax generated 30 positive PTs across 10 SOCs, with 12 on the DMEs list, such as immune thrombocytopenia, autoimmune hepatitis, and thrombotic thrombocytopenic purpura. Compared with Spikevax’s labelling (August 2025) [23], new signals were detected, including some on the DMEs list (e.g., immune thrombocytopenia, autoimmune hepatitis, thrombotic thrombocytopenic purpura, rhabdomyolysis, hemolysis, aplastic anemia, acute kidney injury, autoimmune hemolytic anemia, and Stevens–Johnson syndrome) and other positive PTs (e.g., bacille calmette–guerin scar reactivation, guillain–barre syndrome, and haematuria). Comirnaty Bivalent showed only 1 positive PT, namely, immune thrombocytopenia, on the DMEs list. Spikevax Bivalent had only 1 positive PT, namely lymphadenopathy.

Monovalent mRNA vaccines generated 37 positive PTs across 15 SOCs, with 20 on the DMEs list, such as immune thrombocytopenia, thrombotic thrombocytopenic purpura, and autoimmune hepatitis. Bivalent mRNA vaccines generated 2 positive PTs, including lymphadenopathy and immune thrombocytopenia (on the DMEs list).

#### 3.2.3. TTO Analysis

AEFIs occurred later in the use of monovalent mRNA vaccines (median = 7.0 days, IQR: 2.0–14.0 days) than in the use of bivalent mRNA vaccines (median = 3.0 days, IQR: 2.0–3.8 days). Comirnaty Bivalent (median = 2.0 days, IQR: 2.0–4.5 days) and Spikevax Bivalent (median = 3.0 days, IQR: 2.5–3.5 days) generated earlier AEFIs than Comirnaty (median = 7.0 days, IQR: 2.0–14.0 days) and Spikevax (median = 5.0 days, IQR: 2.0–14.0 days) (Figure 2b). Among all mRNA vaccines, Comirnaty Bivalent (median = 60.0 days, IQR: 60.0–90.0 days) had the longest duration (Figure 2c).

#### 3.2.4. SAEs Analysis

The median age of individuals with SAEs (see Table S14) was generally older than that of those with AEFIs (Table 1), indicating a higher risk in older adults. Unlike VAERS, most SAEs were more frequent in males, but Comirnaty caused more SAEs in females. Meanwhile, an older median age was linked to a greater proportion of vaccinees with medical histories, reflecting a cumulative risk for SAEs. Additionally, multiple SAEs were related to respiratory, thoracic and mediastinal disorders (e.g., breakthrough COVID-19) and cardiac disorders (e.g., myocarditis, myopericarditis) (see Table S15).

#### 3.2.5. Immunization Schedule Analysis

SAEs during primary and booster immunization were analyzed (see Table S16). For all mRNA vaccines and monovalent mRNA vaccines, the number of SAEs was larger in primary immunization, but their proportion was higher in booster immunization. For bivalent mRNA vaccines, only the primary immunization result was detected. The highest incidence was in the primary immunization with Comirnaty Bivalent (50.00%), and the lowest was in the booster immunization with Comirnaty (32.31%). Other SAEs had incidences around 40%. PTs leading to SAEs, including breakthrough COVID-19, myocarditis, pericarditis, guillain–barre syndrome, and immune thrombocytopenia, shared a similar distribution (see Table S17).

### 3.3. Cross-Validation of Multi-Source Signals with Positive Signals in the VAERS and ALD

**Figure 3:**
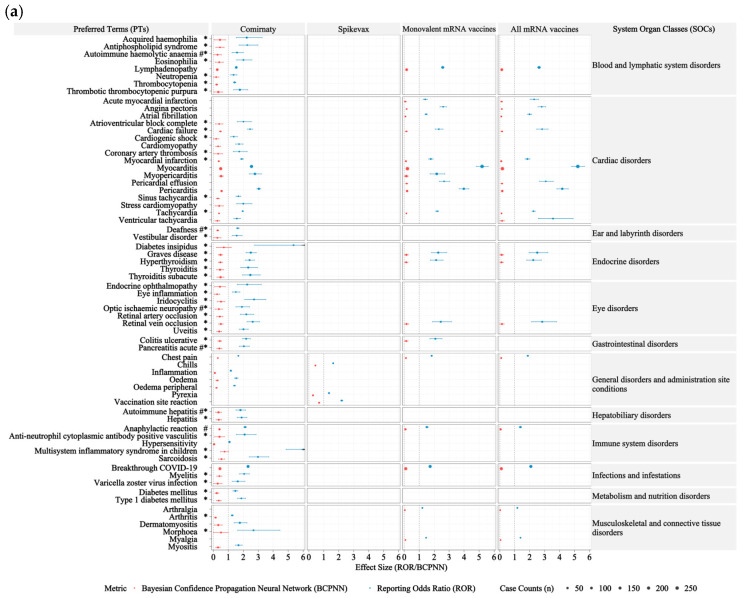

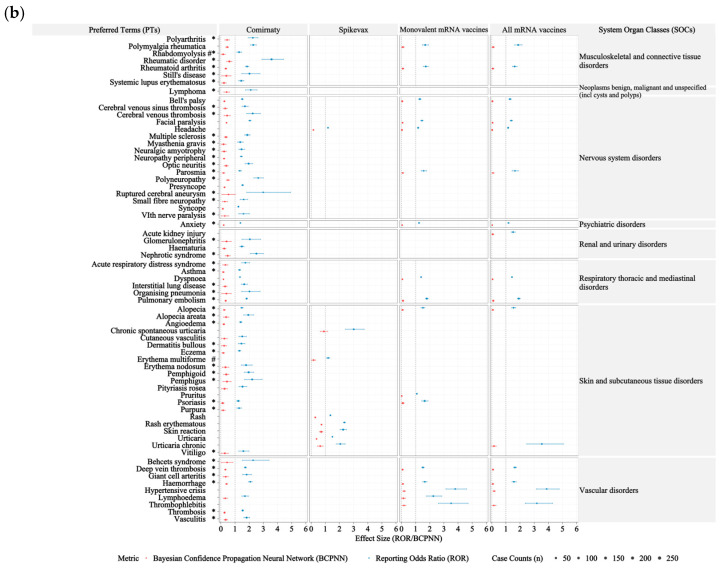
Cross-validation of positive PTs in the VAERS and ALD. (**a**) The upper panel represents. (**b**) The lower panel represents. # indicates PTs within the DME list; * indicates signals not listed in the Comirnaty labelling; → indicates values exceeding the axis limit.

AEFIs with positive signals at the PT level from ALD were cross-validated with the DPA results from VAERS (Figure 3).

Comirnaty was detected in 111 positive PTs across 19 SOCs, with 9 on the DMEs list, including thrombotic thrombocytopenic purpura, autoimmune hemolytic anemia, and anaphylactic reaction. Compared with Comirnaty’s labelling (August 2025) [21], new signals were detected, including some on the DMEs list (e.g., thrombotic thrombocytopenic purpura, autoimmune hepatitis, rhabdomyolysis, optic ischaemic neuropathy, pancreatitis acute, autoimmune hemolytic anemia, and deafness), and other positive PTs (e.g., thyroiditis subacute, multiple sclerosis, pemphigoid, graves’ disease, and rheumatic disorder). Spikevax was detected in 11 positive PTs across 3 SOCs, with 1 on the DMEs list: erythema multiforme. Comirnaty Bivalent and Spikevax Bivalent were not detected as positive PT.

Notably, after cross-validating the positive signals of Comirnaty, monovalent mRNA vaccines, and all mRNA vaccines, breakthrough COVID-19 and lymphadenopathy were frequently reported in the literature case reports and showed high signals with narrow 95% CIs in DPA results. In Spikevax, skin reactions were common in the literature case reports, and likewise demonstrated high signals with narrow 95% CIs in DPA results.

### 3.4. Myocarditis and Pericarditis in VAERS and ALD

Based on the age–sex stratified analysis in Table S18, myocarditis and pericarditis following mRNA vaccines showed clear sex- and age-related distribution patterns. No cases of myocarditis or pericarditis were detected for mRESVIA.

In VAERS, both myocarditis and pericarditis were reported more frequently in males (11,286 and 6023 cases) than in females (5806 and 5451 cases). Across all mRNA vaccines as well as in analyses of individual vaccines, most reports with the age field of both myocarditis and pericarditis were aggregated in the 18–44 years age group. However, analysis of the median age of myocarditis and pericarditis reports showed that males tended to be younger than females. For all mRNA vaccines, the median ages of males with myocarditis and pericarditis were 27 and 35 years, compared with 40 and 45 years among females. A similar pattern was observed for each individual vaccine. In contrast, death reports regarding myocarditis and pericarditis (332 males vs. 171 females) were predominantly observed in older adults, particularly those aged ≥65 years.

A similar pattern was observed in ALD, where reports were more common in males (214 and 36 cases) than in females (63 and 16 cases). Across all mRNA vaccines as well as in analyses of individual vaccines, most reports with the age field of both myocarditis and pericarditis were aggregated in the 18–44 years age group. However, analysis of the median age of myocarditis and pericarditis reports showed that males tended to be younger than females. For all mRNA vaccines, the median ages of males were 22 and 36 years, compared with 37 and 59 years among females. A similar pattern was observed for each individual vaccine. Death cases were rarely reported in ALD, were mainly observed in males, and tended to occur in adults and older individuals.

Overall, myocarditis and pericarditis were reported in both males and females, with a higher reporting frequency in males and a predominance in younger adults aged 18–44 years. Death outcomes were more commonly reported in older adults, particularly those aged ≥65 years.

## 4. Discussion

As their benefits outweigh known risks in the pandemic period, mRNA vaccines are considered a critical tool in combating infectious diseases globally and are expected to open a window in the treatment of cancers. To assess the safety of all mRNA vaccines after Emergency Use Authorization (EUA) or approval, we analyzed 5,040,725 VAERS case reports and 4387 literature case reports over a 5-year period. Among monovalent mRNA vaccines, Comirnaty caused more AEFIs than Spikevax and mRESVIA. Among bivalent mRNA vaccines, Comirnaty Bivalent caused more AEFIs than Spikevax Bivalent. However, AEFIs incidence could not be calculated, due to the lack of vaccination dose data. Comparative studies show that the occurrence of Comirnaty-associated AEFIs is lower than that of Spikevax [26]. Sales data from the U.S. Securities and Exchange Commission (SEC) indicate Comirnaty has a wider application, which may explain the larger number of Comirnaty-related reports [27,28]. Therefore, vaccine safety cannot be evaluated solely by AEFI reports [29].

As shown in Table S19, the AEFIs of mRNAs in RWD were much more diverse than in clinical trials, likely due to the controlled environments and restricted populations in clinical trials, compared to the broader, the larger number of populations in the real world. These factors highlight the importance of post-marketing safety monitoring, as illustrated by the detection of intussusception following the rotavirus vaccine [30], guillain–barre syndrome following the 2009-H1N1 influenza vaccine [31], myocarditis and pericarditis following mRNA COVID-19 vaccine [32], and syncope and thromboembolic events following HPV vaccine through large-scale vaccine safety surveillance systems [33].

We identified a series of statistically positive signals included on the DMEs list but not labelled. In VAERS and ALD, new signals for Comirnaty included thrombotic thrombocytopenic purpura, autoimmune hemolytic anemia, and deafness. In the literature case reports, new signals for Spikevax included immune thrombocytopenia and autoimmune hepatitis. Additionally, for Spikevax, VAERS and ALD detected multiple general disorders and administration site conditions; skin reactions were common in the literature case reports, showing a high signal with narrow 95% CIs in VAERS DPA results. This may relate to the sizes of lipid nanoparticles (LNPs) of vaccines [34,35,36]. No new signals on the DMEs list were observed from other mRNA vaccines. The limited data for mRESVIA and MNEXSPIKE may explain the lack of new signals, warranting further monitoring in larger populations. Previous post-marketing studies have reported menstrual-related AEFIs following administration of all types of COVID-19 vaccines, including mRNA vaccines [37,38,39]. Potential mechanisms may involve vaccine-induced immune-inflammatory responses and prolactin-related signalling pathways. In the present study, menstrual-related positive PTs were likewise detected in VAERS, predominantly for mRNA COVID-19 vaccines. Although causality cannot be established between these menstrual adverse events and mRNA vaccines, the high reporting rate of relevant PTs among mRNA vaccines warrants further investigation.

In the TTO results, the AEFIs of monovalent mRNA vaccines (median = 1.0 day, IQR: 0.0–9.0 days) occurred earlier than those of bivalent mRNA vaccines (median = 10.0 days, IQR: 1.0–86.0 days) in VAERS. In contrast, the AEFIs of monovalent mRNA vaccines (median = 7.0 days, IQR: 2.0–14.0 days) occurred later than those of bivalent mRNA vaccines (median = 3.0 days, IQR: 2.0–3.8 days) in ALD. This discrepancy may be related to the limited number of reports on bivalent mRNA vaccines in ALD and may vary across specific AEFIs, warranting further investigation.

Although breakthrough COVID-19 ranked highly among reported cases, Antibody-Dependent Enhancement (ADE) has not been demonstrated for mRNA COVID-19 vaccines in phase III trials or real-world studies [40,41]. In phase III trials, Comirnaty was effective in preventing COVID-19 among 95% of the recipients [42], while Spikevax efficacy was 94.1% [43]. This indicates high but incomplete protection and therefore an expected occurrence of breakthrough infections. In addition, studies of Comirnaty Bivalent, Spikevax Bivalent, and MNEXSPIKE also suggest that vaccine effectiveness may not provide complete protection for all recipients [25,44,45]. Notably, breakthrough COVID-19 was not a positive safety signal for each mRNA COVID-19 vaccine, such as Spikevax and MNEXSPIKE. Several additional factors may further contribute to the high reporting frequency of breakthrough COVID-19. First, during the pandemic period, sustained viral transmission, continuous viral evolution, and the emergence of immune-evasive variants could increase the likelihood of post-vaccination infection [46]. Second, the protective efficacy of vaccines is influenced by the match between vaccine antigens and circulating strains, which may decline as new variants emerge [46]. Third, immunity after vaccination is not permanent, and waning immune protection over time may contribute to breakthrough infections [40,41,47,48,49]. Fourth, in seasonal epidemic settings, individuals may already have been exposed to SARS-CoV-2 before sufficient protective antibody levels were generated after vaccination, particularly when vaccination was administered shortly before exposure [46]. Finally, regional differences in nucleic acid testing policies, surveillance intensity, and diagnostic capacity may also affect the number of reported breakthrough COVID-19 cases.

We observed that older adults (particularly with a medical history) might be prone to SAEs from vaccination in both VAERS and ALD. In older adults, functional decline and immunosenescence increase the risk of infections and slow the development of protective immunity [50,51,52]. The higher proportion of SAEs in females may be due to their stronger health awareness and proactive medical care [52]. Notably, males accounted for more DIED cases in VAERS and more SAEs in ALD. Furthermore, in both overall reports and death reports, myocarditis and pericarditis following mRNA COVID-19 vaccination were reported more frequently in males than in females. Affected males presented with a younger median age compared with females, whereas death cases were predominantly observed among older adults (Table S18). This difference is likely related to biological mechanisms. Previous studies show higher susceptibility to myocarditis and pericarditis in adolescent and young adult males [8]. Sex steroid hormones influence the immune and inflammatory response [53], and estrogen has been shown to modulate cardiac mast cells and attenuate adverse cardiac remodelling [54,55]. These factors may contribute to the higher reporting of SAE in males. Additionally, dyspnoea is frequently reported as an SAE in VAERS. As dyspnoea may be a clinical manifestation of allergic reactions or myocarditis [56,57], both of which are also commonly reported as SAEs in ALD, persistent monitoring of these three AEFIs is warranted. Although these three AEFIs are already described in the product labelling, they still carry a potential risk of leading to serious adverse reactions. The distribution of SAE varies across the two phases of immunization. Both VAERS and ALD showed a higher proportion of SAEs during the booster immunization, although more SAEs were observed during the primary immunization for all mRNA vaccines and monovalent mRNA vaccines. This may relate to immune response sensitization after primary immunization or population differences. Common PTs leading to SAEs, like myocarditis and immune thrombocytopenia, are similar on both VAERS and ALD. These findings underscore the importance of continued safety monitoring.

However, as a passive surveillance system, VAERS data are subject to underreporting, reporting biases and variable quality that potentially compromise result accuracy. Without access to actual vaccination doses, we failed to calculate the actual incidence of AEFIs. Sales-based indirect estimates were unreliable for cross-vaccine safety comparisons. Therefore, this study focused on safety signal detection and evaluation rather than incidence assessment. VAERS case reports lack detailed medical descriptions and follow-up data, which may only reflect the temporal, not causal, association between AEFIs and mRNA vaccines. In addition, some AEFIs overlap with COVID-19 symptoms, further increasing the complexity of signal interpretation. In contrast, the literature case reports provide more comprehensive medical descriptions, including patient history, laboratory findings, and clinical course, which allow clearer assessment of causality between vaccination and AEFIs. Therefore, we used literature case reports to cross-verify the signals identified by DPA, providing stronger evidence for a potential causal relationship than VAERS alone. Notably, as passive rather than active surveillance sources, both VAERS and ALD provide insufficient evidence for comparing the safety profiles of different vaccines. The signals detected in the present study cannot establish a definitive causal relationship between mRNA vaccines and reported AEFIs. In addition, cognitive biases may influence reporting [58], making monitoring alone insufficient. Moreover, although VAERS and ALD include cases reported from global sources, further studies integrating other spontaneous reporting systems from different geographical regions, as well as comparative analyses involving other vaccines, are warranted in the future.

## 5. Conclusions

In summary, this RWS focused on the safety profiles of all marketed mRNA vaccines with different indications. Statistically positive safety signals of AEFIs across various SOCs were detected, some of which were also included on the DMEs list. Special populations (such as females, older adults, and individuals with underlying medical conditions) had a higher risk of SAEs and should be monitored more closely. Limited by the insufficient accuracy dosage data of mRNA vaccines, this study could not calculate AEFI incidence rates or make direct comparisons of safety profiles among different vaccines. These findings provide solid empirical evidence for improving post-marketing safety surveillance and pharmacovigilance practices, as well as optimizing immunization strategies scientifically.

## Figures and Tables

**Table 1:** Demographic characteristics of mRNA vaccines AEFIs in VAERS and literature case reports.

Characteristic	Comirnaty	Spikevax	mRESVIA	MNEXSPIKE	Comirnaty Bivalent	Spikevax Bivalent	Monovalent mRNA Vaccines	Bivalent mRNA Vaccines	All mRNA Vaccines
AEFIs in **VAERS**									
Age (*n* (%))									
Median	48	53	69	67	65	66	50	65	50
<6 months	406 (0.01)	314 (0.02)	0 (0.00)	0 (0.00)	22 (0.03)	0 (0.00)	720 (0.01)	22 (0.02)	742 (0.01)
6 months–11 years	23,711 (0.76)	4268 (0.24)	0 (0.00)	29 (1.57)	1024 (1.60)	519 (1.07)	28,008 (0.57)	1543 (1.37)	29,551 (0.59)
12–17 years	78,854 (2.52)	4523 (0.25)	0 (0.00)	23 (1.25)	1147 (1.79)	349 (0.72)	83,400 (1.69)	1496 (1.33)	84,896 (1.68)
18–44 years	760,720 (24.34)	598,786 (33.25)	9 (7.38)	290 (15.74)	9247 (14.47)	7160 (14.71)	1,359,805 (27.59)	16,407 (14.58)	1,376,212 (27.30)
45–64 years	664,562 (21.26)	604,897 (33.59)	20 (16.39)	380 (20.63)	17,167 (26.86)	13,617 (27.98)	1,269,859 (25.77)	30,784 (27.35)	1,300,643 (25.80)
≥65 years	415,552 (13.30)	481,594 (26.74)	80 (65.57)	1006 (54.61)	28,931 (45.27)	25,423 (52.25)	898,232 (18.23)	54,354 (48.29)	952,586 (18.90)
Unknown	1,181,479 (37.80)	106,534 (5.92)	13 (10.66)	114 (6.19)	6364 (9.96)	1591 (3.27)	1,288,140 (26.14)	7955 (7.07)	1,296,095 (25.71)
Sex (n (%))									
F	2,133,231 (68.26)	1,291,212 (71.70)	100 (81.97)	1292 (70.14)	41,675 (65.22)	32,593 (66.98)	3,425,835 (69.52)	74,268 (65.98)	3,500,103 (69.44)
M	920,203 (29.44)	477,176 (26.50)	22 (18.03)	519 (28.18)	21,702 (33.96)	15,112 (31.06)	1,397,920 (28.37)	36,814 (32.71)	1,434,734 (28.46)
Unknown	71,850 (2.30)	32,528 (1.81)	0 (0.00)	31 (1.68)	525 (0.82)	954 (1.96)	104,409 (2.12)	1479 (1.31)	105,888 (2.10)
History (n (%))									
No history	222,605 (7.12)	246,213 (13.67)	11 (9.02)	220 (11.94)	7493 (11.73)	5643 (11.60)	469,049 (9.52)	13,136 (11.67)	482,185 (9.57)
With history	826,922 (26.46)	702,360 (39.00)	86 (70.49)	732 (39.74)	32,286 (50.52)	25,419 (52.24)	1,530,100 (31.05)	57,705 (51.27)	1,587,805 (31.50)
Unknown	2,075,757 (66.42)	852,343 (47.33)	25 (20.49)	890 (48.32)	24,123 (37.75)	17,597 (36.16)	2,929,015 (59.43)	41,720 (37.06)	2,970,735 (58.93)
Reported year (n (%))									
2021	1,946,890 (62.29)	1,332,039 (73.96)	0 (0.00)	0 (0.00)	0 (0.00)	0 (0.00)	3,278,929 (66.53)	0 (0.00)	3,278,929 (65.05)
2022	876,680 (28.05)	358,507 (19.91)	0 (0.00)	0 (0.00)	22,545 (35.28)	16,654 (34.23)	1,235,187 (25.06)	39,199 (34.82)	1,274,386 (25.28)
2023	203,029 (6.50)	64,488 (3.58)	0 (0.00)	0 (0.00)	34,631 (54.19)	24,209 (49.75)	267,517 (5.43)	58,840 (52.27)	326,357 (6.47)
2024	69,343 (2.22)	31,433 (1.75)	36 (29.51)	0 (0.00)	5893 (9.22)	7278 (14.96)	100,812 (2.05)	13,171 (11.70)	113,983 (2.26)
2025	29,342 (0.94)	14,449 (0.80)	86 (70.49)	1842 (100.00)	833 (1.30)	518 (1.06)	45,719 (0.93)	1351 (1.20)	47,070 (0.93)
Total	3,125,284	1,800,916	122	1842	63,902	48,659	4,928,164	112,561	5,040,725
AEFIs in the **literature case reports**									
Age (*n* (%))									
Median	47	49	NA	NA	80	47	48	74	48
<6 months	0 (0.00)	0 (0.00)	NA	NA	0 (0.00)	0 (0.00)	0 (0.00)	0 (0.00)	0 (0.00)
6 months–11 years	29 (0.91)	4 (0.34)	NA	NA	0 (0.00)	0 (0.00)	33 (0.76)	0 (0.00)	33 (0.75)
12–17 years	272 (8.49)	13 (1.11)	NA	NA	0 (0.00)	0 (0.00)	285 (6.52)	0 (0.00)	285 (6.50)
18–44 years	1152 (35.96)	447 (38.34)	NA	NA	0 (0.00)	1 (14.29)	1599 (36.59)	1 (5.88)	1600 (36.47)
45–64 years	931 (29.06)	385 (33.02)	NA	NA	0 (0.00)	4 (57.14)	1316 (30.11)	4 (23.53)	1320 (30.09)
≥65 years	776 (24.22)	300 (25.73)	NA	NA	7 (70.00)	2 (28.57)	1076 (24.62)	9 (52.94)	1085 (24.73)
Unknown	44 (1.37)	17 (1.46)	NA	NA	3 (30.00)	0 (0.00)	61 (1.40)	3 (17.65)	64 (1.46)
Sex (n (%))									
F	1689 (52.72)	603 (51.72)	NA	NA	7 (70.00)	3 (42.86)	2292 (52.45)	10 (58.82)	2302 (52.47)
M	1460 (45.57)	551 (47.26)	NA	NA	3 (30.00)	4 (57.14)	2011 (46.02)	7 (41.18)	2018 (46.00)
Unknown	55 (1.72)	12 (1.03)	NA	NA	0 (0.00)	0 (0.00)	67 (1.53)	0 (0.00)	67 (1.53)
History (n (%))									
No history	633 (19.76)	93 (7.98)	NA	NA	0 (0.00)	3 (42.86)	726 (16.61)	3 (17.65)	729 (16.62)
With history	1580 (49.31)	652 (55.92)	NA	NA	6 (60.00)	3 (42.86)	2232 (51.08)	9 (52.94)	2241 (51.08)
Unknown	991 (30.93)	421 (36.11)	NA	NA	4 (40.00)	1 (14.29)	1412 (32.31)	5 (29.41)	1417 (32.30)
Reported year (n (%))									
2021	585 (18.26)	281 (24.10)	NA	NA	0 (0.00)	0 (0.00)	866 (19.82)	0 (0.00)	866 (19.74)
2022	1546 (48.25)	531 (45.54)	NA	NA	0 (0.00)	1 (14.29)	2077 (47.53)	1 (5.88)	2078 (47.37)
2023	730 (22.78)	233 (19.98)	NA	NA	9 (90.00)	4 (57.14)	963 (22.04)	13 (76.47)	976 (22.25)
2024	233 (7.27)	84 (7.20)	NA	NA	0 (0.00)	2 (28.57)	317 (7.25)	2 (11.76)	319 (7.27)
2025	110 (3.43)	37 (3.17)	NA	NA	1 (10.00)	0 (0.00)	147 (3.36)	1 (5.88)	148 (3.37)
Total	3204	1166	NA	NA	10	7	4370	17	4387

*n*: Number of reports. NA: Non-positive signal, indicating that the signal detection thresholds for both the ROR and BCPNN methods were not met.

## Data Availability

All data analyzed in this study are public data from the VAERS (https://vaers.hhs.gov/data/datasets.html, accessed on 3 January 2026) and the ALD, including PubMed (https://pubmed.ncbi.nlm.nih.gov/, accessed on 3 January 2026), Embase (https://www.embase.com/search/quick, accessed on 3 January 2026), Web of Science (https://www.webofscience.com/wos/alldb/basic-search, accessed on 3 January 2026) and Scopus (https://www.scopus.com, accessed on 3 January 2026).

## Supplementary Materials

The following supporting information can be downloaded at: https://www.mdpi.com/article/10.3390/vaccines14060524/s1, Table S1: Basic information of mRNA vaccines; Table S2: PICOS of literature case reports; Table S3: AEFI terms without clear clinical significance; Table S4: Two-by-two contingency table for DPA; Table S5: The principles of disproportionate measurement and the criteria for signal detection of DPA; Table S6: Search terms of literature case reports; Table S7: Top-ranked positive PT of mRNA vaccines in VAERS; Table S8: All positive PT of mRNA vaccines in VAERS; Table S9: Demographic characteristics of SAEs in VAERS; Table S10: Top 10 PTs Leading to SAEs in VAERS; Table S11: Immunization schedules and SAEs analysis in VAERS; Table S12: Top 10 PTs Leading to SAEs during primary and booster immunization in VAERS; Table S13: Top-ranked positive PT of mRNA vaccines in literature case reports; Table S14: Demographic characteristics of SAEs in literature case reports; Table S15: Top 10 PTs Leading to SAEs in literature case reports; Table S16: Immunization schedules and SAEs analysis in literature case reports; Table S17: Top 10 PTs Leading to SAEs during primary and booster immunization in literature case reports; Table S18: The age-sex stratified analysis of myocarditis and pericarditis following mRNA vaccines; Table S19: Safety characteristics of mRNA vaccines in clinical trials.

## Abbreviations

The following abbreviations are used in this manuscript:

ADE	Antibody-Dependent Enhancement
AEFI	Adverse Events Following Immunization
ALD	Academic Literature Databases
BCPNN	Bayesian Confidence Propagation Neural Network
CDC	Centres for Disease Control and Prevention
CI	Confidence/Credible interval
DME	Designated Medical Event
DPA	Disproportionality Analysis
EMA	European Medicines Agency
EUA	Emergency Use Authorization
FDA	Food and Drug Administration
IQR	Interquartile range
LNP	lipid nanoparticle
MedDRA	Medical Dictionary for Regulatory Activities
PICOS	Population, Intervention, Comparison, Outcomes, Study
PRISMA	Preferred Reporting Items for Systematic Reviews and Meta-Analyses 2020 guidelines
PT	Preferred Term
READUS-PV	REporting of A Disproportionality analysis for drUg Safety signal detection using spontaneous case reports in PharmacoVigilance
ROR	Reporting Odds Ratio
RSV	Respiratory Syncytial Virus
RWD	Real-World Data
RWS	Real-World Study
SAE	Serious Adverse Event
SOC	System Organ Class
TTO	Time-to-onset
VAERS	Vaccine Adverse Event Reporting System
