# Supplementary material for: Post-Marketing Safety of mRNA Vaccines: A Real-World Study Integrating Literature Case Reports and Vaccine Adverse Event Reporting System
Source: Vaccines (Basel). 2026 Jun 12;14(6):524. doi: 10.3390/vaccines14060524 (PMC13308135; doi:10.3390/vaccines14060524)
Supplement: Supplementary file 1 [file vaccines-14-00524-s001.zip › Table S1.pdf]

Table S1. Basic information of mRNA vaccines

Brand Name	Antigen Design	Indications	Market Launch Time	Licensing Authority	Marketing Authorization Holder	Research Cross-Section
Comirnaty	SARS-CoV-2 full-length spike protein	COVID-19	December 11, 2020	FDA	Pfizer BioNTech	January 1, 2021-December 31, 2025
Spikevax	SARS-CoV-2 full-length spike protein	COVID-19	December 11, 2020	FDA	Moderna	January 1, 2021-December 31, 2025
Comirnaty Bivalent	SARS-CoV-2 full-length spike protein, and Omicron BA.4/5 Spike	COVID-19	August 31, 2022	FDA	Pfizer BioNTech	September 1, 2022-December 31, 2025
Spikevax Bivalent	SARS-CoV-2 full-length spike protein, and Omicron BA.4/5 Spike	COVID-19	August 31, 2022	FDA	Moderna	September 1, 2022-December 31, 2025
mRESVIA	RSV F glycoprotein stabilized in the prefusion conformation (preF protein)	RSV	May 31, 2024	FDA	Moderna	June 1, 2024-December 31, 2025
MNEXSPIKE	Receptor binding domain (RBD) and N-terminal domain (NTD) of the SARS-CoV-2 spike protein	COVID-19	May 30, 2025	FDA	Moderna	June 1, 2025-December 31, 2025
