# Supplementary material for: Post-Marketing Safety of mRNA Vaccines: A Real-World Study Integrating Literature Case Reports and Vaccine Adverse Event Reporting System
Source: Vaccines (Basel). 2026 Jun 12;14(6):524. doi: 10.3390/vaccines14060524 (PMC13308135; doi:10.3390/vaccines14060524)
Supplement: Supplementary file 1 [file vaccines-14-00524-s001.zip › Table S10.pdf]

Table S10. Top 10 PTs Leading to SAEs in VAERS.

Vaccines	DIED	L_THREAT	HOSPITAL	X_STAY	DISABLE	BIRTH_DEFECT	All SAEs
**Comirnaty**	Death（10208）	Dyspnoea（3929）	Breakthrough COVID-19（28439）	Pyrexia（294）	Fatigue（12321）	Abortion spontaneous（181）	Breakthrough COVID-19（32198）
	Breakthrough COVID-19（6697）	Pulmonary embolism（2820）	Dyspnoea（20083）	Dyspnoea（245）	Headache（10606）	Foetal death（44）	Fatigue（26387）
	Dyspnoea（2966）	Fatigue（2287）	Pyrexia（15138）	Headache（231）	Arthralgia（7545）	Foetal growth restriction（43）	Dyspnoea（25972）
	Pyrexia（1817）	Headache（2256）	Headache（14638）	Fatigue（185）	Myalgia（7302）	Congenital anomaly（39）	Headache（25572）
	Cardiac arrest（1617）	Pyrexia（2161）	Fatigue（13894）	Breakthrough COVID-19（170）	Pyrexia（6498）	Fatigue（38）	Pyrexia（22989）
	Asthenia（1229）	Chest pain（2123）	Chest pain（11715）	Dizziness（165）	Pain in extremity（5679）	Headache（35）	Dizziness（16441）
	General physical health deterioration（1143）	Dizziness（1615）	Dizziness（11263）	Chest pain（158）	Nausea（5337）	Dyspnoea（35）	Chest pain（14797）
	Malaise（1135）	Cerebrovascular accident（1372）	Asthenia（9744）	Vomiting（120）	Dizziness（5020）	Dizziness（34）	Nausea（14585）
	Fatigue（1098）	Myocarditis（1346）	Nausea（8761）	Malaise（117）	Pain（4913）	Pyrexia（34）	Asthenia（13492）
	Pneumonia（1061）	Malaise（1335）	Malaise（7982）	Asthenia（108）	Malaise（4585）	Stillbirth（27）	Malaise（13390）
**Spikevax**	Death（7667）	Dyspnoea	Breakthrough	Pyrexia（65）	Fatigue	Abortion	Breakthrough

		（1582）	COVID-19（18377）		（4177）	spontaneous（68）	COVID-19（20314）
	Breakthrough COVID-19（4111）	Pulmonary embolism（1152）	Dyspnoea（8293）	Dyspnoea（47）	Headache（3833）	Pyrexia（37）	Pyrexia（10526）
	Dyspnoea（1461）	Pyrexia（976）	Pyrexia（6705）	Headache（43）	Pyrexia（3315）	Fatigue（31）	Dyspnoea（10279）
	Cardiac arrest（730）	Fatigue（923）	Fatigue（4971）	Fatigue（38）	Myalgia（2827）	Headache（29）	Fatigue（9360）
	Pyrexia（729）	Headache（823）	Headache（4586）	Dizziness（31）	Arthralgia（2538）	Pain（23）	Headache（8598）
	Asthenia（662）	Chest pain（801）	Asthenia（4131）	Breakthrough COVID-19（28）	Chills（2329）	Chills（16）	Death（7671）
	General physical health deterioration（590）	Cerebrovascular accident（613）	Chest pain（3861）	Chest pain（27）	Nausea（2183）	Pain in extremity（15）	Nausea（5685）
	Pneumonia（537）	Dizziness（581）	Cough（3483）	Nausea（26）	Malaise（1992）	Dizziness（15）	Asthenia（5371）
	Fatigue（510）	Asthenia（569）	Dizziness（3369）	Chills（25）	Pain in extremity（1719）	Nausea（15）	Chills（5189）
	Cough（494）	Nausea（539）	Nausea（3311）	Myalgia（24）	Pain（1582）	Dyspnoea（14）	Myalgia（5090）
**mRESVIA**	Dysarthria（1）	Basal ganglia haemorrhage（1）	Delivery（1）	NA	Bell's palsy（1）	Chills（1）	Bell's palsy（1）
	Dyspnoea（1）	Blood brain barrier defect（1）	Induced labour（1）	NA	NA	Cough（1）	Chills（1）

	Feeling abnormal（1）	Brain oedema（1）	Pre-eclampsia（1）	NA	NA	Headache（1）	Cough（1）
	Malaise（1）	Cerebral haemorrhage(1）	Hypoaesthesia(1）	NA	NA	Myalgia（1）	Headache（1）
	Paranasal sinus hypersecretion（1）	Cerebral mass effect（1）	Limb discomfort（1）	NA	NA	Nausea（1）	Myalgia（1）
	Productive cough（1）	Depressed level of consciousness（1）	Muscular weakness（1）	NA	NA	Pain（1）	Nausea（1）
	Respiratory tract congestion（1）	Encephalopathy（1）	Paraesthesia（1）	NA	NA	NA	Pain（1）
	Rhonchi（1）	Hydrocephalus（1）	Rash（1）	NA	NA	NA	Dysarthria（1）
	Sputum discoloured（1）	Hypopnoea（1）	Tinea infection(1）	NA	NA	NA	Dyspnoea（1）
	Sudden death（1）	Intraventricular haemorrhage(1）	Basal ganglia haemorrhage（1）	NA	NA	NA	Feeling abnormal（1）
**MNEXSPIKE**	Death（8）	Myocarditis（5）	Pyrexia（9）	Pulmonary embolism（1）	Anxiety（4）	Abdominal distension（1）	Pyrexia（11）
	Dyspnoea（3）	Asthenia（3）	Asthenia（6）	NA	Fatigue（4）	Abdominal pain（1）	Death（8）
	Dysphagia（2）	Confusional state（3）	Headache（5）	NA	Arthralgia（4）	Amnesia（1）	Asthenia（6）
	Pyrexia（1）	Headache（3）	Vomiting（5）	NA	Burning sensation（3）	Anaphylactic reaction（1）	Vomiting（6）

	Vomiting（1）	Loss of consciousness（3）	Confusional state（4）	NA	Gait disturbance（3）	Anxiety（1）	Dyspnoea（6）
	Amyotrophic lateral sclerosis（1）	Pyrexia（3）	Dizziness（4）	NA	Nausea（3）	Arthralgia（1）	Dizziness（5）
	Bulbar palsy（1）	Vomiting（3）	Muscular weakness（4）	NA	Dizziness（3）	Bone pain（1）	Headache（5）
	Speech disorder（1）	Dyspnoea（3）	Anxiety（4）	NA	Muscular weakness（3）	Brain fog（1）	Muscular weakness（5）
	Throat tightness（1）	Hypertension（3）	Nausea（4）	NA	Sleep disorder（2）	Condition aggravated（1）	Anxiety（5）
	Pneumonia（1）	Unresponsive to stimuli（2）	Myocarditis（4）	NA	Tinnitus（2）	Confusional state（1）	Myocarditis（5）
**Comirnaty Bivalent**	Death（202）	Dyspnoea（74）	Breakthrough COVID-19（1117）	Confusional state（2）	Fatigue（103）	Pain（3）	Breakthrough COVID-19（1156）
	Breakthrough COVID-19（83）	Fatigue（55）	Acute respiratory failure（459）	Cognitive disorder（2）	Arthralgia（62）	Arthralgia（2）	Dyspnoea（480）
	Dyspnoea（39）	Pulmonary embolism（51）	Dyspnoea（419）	T-cell prolymphocytic leukaemia（1）	Headache（59）	Muscular weakness（2）	Acute respiratory failure（464）
	Cardiac arrest（32）	Pyrexia（47）	Hypoxia（323）	Exercise tolerance decreased（1）	Dizziness（55）	Pain in extremity（2）	Asthenia（365）
	General physical health deterioration（32）	Cerebrovascular accident（39）	Asthenia（322）	Feeling abnormal（1）	Dyspnoea（53）	Ectopic pregnancy with contraceptive	Hypoxia（330）

						device（1）	
	Acute respiratory failure（30）	Asthenia（35）	Cough（289）	Gait disturbance（1）	Pain（52）	Haemorrhage（1）	Pyrexia（321）
	Asthenia（29）	Chest pain（33）	Pyrexia（272）	Loss of control of legs（1）	Pyrexia（52）	Pregnancy（1）	Cough（309）
	Malaise（28）	Atrial fibrillation（28）	Condition aggravated（265）	Muscle tightness（1）	Tinnitus（52）	Bone pain（1）	Condition aggravated（306）
	Unresponsive to stimuli（23）	Palpitations（28）	Symptom recurrence（207）	Poor quality sleep（1）	Malaise（48）	Bursitis（1）	Fatigue（301）
	Hypotension（23）	Dizziness（26）	Fatigue（206）	Sensory loss（1）	Myalgia（47）	Joint range of motion decreased（1）	Hypotension(211）
**Spikevax Bivalent**	Death（112）	Dyspnoea（41）	Breakthrough COVID-19（632）	Appendix cancer（1）	Fatigue（76）	Fatigue（1）	Breakthrough COVID-19（646）
	Neoplasm malignant（28）	Chest pain（21）	Dyspnoea（211）	Dyspnoea exertional（1）	Headache（64）	Pyrexia（1）	Dyspnoea（255）
	Breakthrough COVID-19（27）	Fatigue（20）	Acute respiratory failure（155）	Cough（1）	Pain in extremity（49）	Cerebral artery occlusion（1）	Pyrexia（175）
	Dyspnoea（21）	Pulmonary embolism（19）	Pyrexia（132）	Dizziness（1）	Pyrexia（44）	Left-to-right cardiac shunt（1）	Fatigue（171）
	Unresponsive to stimuli（13）	Asthenia（18）	Asthenia（128）	Headache（1）	Dyspnoea（42）	Muscular weakness（1）	Acute respiratory failure（157）
	Pneumonia（11）	Atrial fibrillation（18）	Cough（121）	Lymph node pain（1）	Dizziness（40）	Speech disorder（1）	Asthenia（152）
	Cardiac arrest（11）	Headache（17）	Hypoxia（110）	Lymphadenopathy	Pain（37）	Deep vein	Cough（135）

				（1）		thrombosis（1）	
	Condition aggravated（11）	Pyrexia（16）	Condition aggravated（105）	Malaise（1）	Nausea（36）	Eye disorder（1）	Condition aggravated（129）
	Acute respiratory failure（11）	Malaise（15）	Fatigue（102）	Nausea（1）	Arthralgia（32）	Pharyngeal disorder（1）	Headache（124）
	Asthenia（10）	Cerebrovascular accident（13）	Chest pain（79）	Skin warm（1）	Chest pain（28）	Renal pain（1）	Death（112）
**Monovalent mRNA vaccines**	Death（17883）	Dyspnoea（5514）	Breakthrough COVID-19（46817）	Pyrexia（359）	Fatigue（16502）	Abortion spontaneous(249）	Breakthrough COVID-19（52514）
	Breakthrough COVID-19（10808）	Pulmonary embolism（3974）	Dyspnoea（28379）	Dyspnoea（292）	Headache（14441）	Pyrexia（71）	Dyspnoea（36258）
	Dyspnoea（4431）	Fatigue（3212）	Pyrexia（21852）	Headache（274）	Myalgia（10129）	Fatigue（70）	Fatigue（35751）
	Pyrexia（2547）	Pyrexia（3140）	Headache（19229）	Fatigue（223）	Arthralgia（10087）	Headache（66）	Headache（34176）
	Cardiac arrest（2348）	Headache（3082）	Fatigue（18867）	Breakthrough COVID-19（198）	Pyrexia（9814）	Foetal growth restriction（57）	Pyrexia（33526）
	Asthenia（1892）	Chest pain（2925）	Chest pain（15577）	Dizziness（196）	Nausea（7523）	Foetal death（56）	Dizziness（21308）
	General physical health deterioration（1733）	Dizziness（2198）	Dizziness（14636）	Chest pain（185）	Pain in extremity（7400）	Dyspnoea（50）	Nausea（20275）
	Fatigue（1608）	Cerebrovascular accident（1985）	Asthenia（13881）	Vomiting（144）	Malaise（6578）	Dizziness（49）	Chest pain(19602）
	Malaise（1606）	Nausea（1850）	Nausea（12076）	Malaise（135）	Pain（6497）	Pain（49）	Asthenia（18869）

	Pneumonia（1599）	Myocarditis（1799）	Malaise（10685）	Nausea（128）	Chills(6458）	Congenital anomaly（41）	Malaise（18352）
**Bivalent mRNA vaccines**	Death（314）	Dyspnoea（115）	Breakthrough COVID-19（1749）	Confusional state（2）	Fatigue（179）	Pain（3）	Breakthrough COVID-19（1802）
	Breakthrough COVID-19（110）	Fatigue（75）	Dyspnoea（630）	Cognitive disorder（2）	Headache（123）	Muscular weakness（3）	Dyspnoea（735）
	Dyspnoea（60）	Pulmonary embolism（70）	Acute respiratory failure（614）	T-cell prolymphocytic leukaemia（1）	Pyrexia（96）	Arthralgia（2）	Acute respiratory failure（621）
	Cardiac arrest（43）	Pyrexia（63）	Asthenia（450）	Exercise tolerance decreased（1）	Dyspnoea（95）	Pain in extremity（2）	Asthenia（517）
	Acute respiratory failure（41）	Chest pain（54）	Hypoxia（433）	Feeling abnormal（1）	Pain in extremity（95）	Fatigue（2）	Pyrexia（496）
	General physical health deterioration（41）	Asthenia（53）	Cough（410）	Gait disturbance（1）	Dizziness（95）	Ectopic pregnancy with contraceptive device（1）	Fatigue（472）
	Asthenia（39）	Cerebrovascular accident（52）	Pyrexia（404）	Loss of control of legs（1）	Arthralgia（94）	Haemorrhage（1）	Cough（444）
	Unresponsive to stimuli（36）	Atrial fibrillation（46）	Condition aggravated（370）	Muscle tightness（1）	Pain（89）	Pregnancy（1）	Hypoxia（442）
	Malaise（34）	Headache（43）	Fatigue（308）	Poor quality sleep（1）	Tinnitus（76）	Bone pain（1）	Condition aggravated（435）
	Condition aggravated（34）	Malaise（41）	Hypotension（260）	Sensory loss（1）	Myalgia（74）	Bursitis（1）	Death（314）

**All mRNA vaccines**	Death（18197）	Dyspnoea（5629）	Breakthrough COVID-19（48566）	Pyrexia（360）	Fatigue（16681）	Abortion spontaneous（249）	Breakthrough COVID-19（54316）
	Breakthrough COVID-19（10918）	Pulmonary embolism（4044）	Dyspnoea（29009）	Dyspnoea（293）	Headache（14564）	Pyrexia（72）	Dyspnoea（36993）
	Dyspnoea（4491）	Fatigue（3287）	Pyrexia（22256）	Headache（275）	Myalgia（10203）	Fatigue（72）	Fatigue（36223）
	Pyrexia（2560）	Pyrexia（3203）	Headache（19384）	Fatigue（223）	Arthralgia（10181）	Headache（66）	Headache（34456）
	Cardiac arrest（2391）	Headache（3125）	Fatigue（19175）	Breakthrough COVID-19（199）	Pyrexia（9910）	Foetal death（57）	Pyrexia（34022）
	Asthenia（1931）	Chest pain（2979）	Chest pain（15801）	Dizziness（197）	Nausea（7587）	Foetal growth restriction（57）	Dizziness（21554）
	General physical health deterioration（1774）	Dizziness（2234）	Dizziness（14796）	Chest pain（185）	Pain in extremity（7495）	Pain（52）	Nausea（20531）
	Malaise（1640）	Cerebrovascular accident（2037）	Asthenia（14331）	Vomiting（145）	Malaise（6646）	Dyspnoea（50）	Chest pain（19879）
	Pneumonia（1632）	Nausea（1873）	Nausea（12265）	Malaise（136）	Pain（6586）	Dizziness（49）	Asthenia（19386）
	Fatigue（1631）	Malaise（1836）	Cough（10943）	Nausea（129）	Dizziness（6530）	Congenital anomaly（41）	Malaise（18660）

DIED: died; L_THREAT: life threatening; HOSPITAL: hospitalized; X_STAY: prolonged hospitalization; DISABLE: disability; BIRTH_DEFECT: Congenital anomaly or birth defect.
