# Supplementary material for: Post-Marketing Safety of mRNA Vaccines: A Real-World Study Integrating Literature Case Reports and Vaccine Adverse Event Reporting System
Source: Vaccines (Basel). 2026 Jun 12;14(6):524. doi: 10.3390/vaccines14060524 (PMC13308135; doi:10.3390/vaccines14060524)
Supplement: Supplementary file 1 [file vaccines-14-00524-s001.zip › Table S11.pdf]

Table S11. Immunization schedules and SAEs analysis in VAERS.

Vaccines	Immunization schedule	DIED (n (%))	L_THREAT (n (%))	HOSPITAL (n (%))	X_STAY (n (%))	DISABLE (n (%))	BIRTH_DEFECT (n (%))	All SAEs (n (%))	SAE (%)
**Comirnaty**	Primary	57455 (69.15)	78436 (70.96)	427696 (70.06)	5726 (71.60)	187593 (72.55)	1935 (75.20)	634555 (70.50)	28.39
	Booster	12652 (15.23)	17543 (15.87)	107489 (17.61)	1113 (13.92)	43235 (16.72)	313 (12.16)	153432 (17.05)	31.67
	Unknown	12983 (15.63)	14564 (13.17)	75305 (12.34)	1158 (14.48)	27729 (10.72)	325 (12.63)	112124 (12.46)	NA
	Total	83090	110543	610490	7997	258557	2573	900111	28.97
**Spikevax**	Primary	30452 (73.28)	27076 (65.49)	149574 (64.86)	1261 (78.42)	54316 (61.92)	718 (66.11)	213797 (64.00)	16.31
	Booster	5595 (13.46)	6448 (15.60)	47964 (20.80)	73 (4.54)	21152 (24.12)	132 (12.15)	70374 (21.07)	26.77
	Unknown	5509 (13.26)	7821 (18.92)	33060 (14.34)	274 (17.04)	12245 (13.96)	236 (21.73)	49883 (14.93)	NA
	Total	41556	41345	230598	1608	87713	1086	334054	18.05
**mRESVIA**	Primary	NA	NA	3 (13.04)	0 (NaN)	NA	6 (100.00)	9 (22.50)	14.52
	Unknown	10 (100.00)	14 (100.00)	20 (86.96)	0 (NaN)	1 (100.00)	NA	31 (77.50)	NA
	Total	10	14	23	NA	1	6	40	14.52
**MNEXSPIKE**	Primary	18 (52.94)	89 (69.53)	154 (64.98)	1 (100.00)	168 (91.30)	39 (100.00)	234 (65.92)	38.30
	Booster	5 (14.71)	8 (6.25)	23 (9.70)	NA	NA	NA	30 (8.45)	15.62
	Unknown	11 (32.35)	31 (24.22)	60 (25.32)	NA	16 (8.70)	NA	91 (25.63)	NA
	Total	34	128	237	1	184	39	355	32.88
**Comirnaty Bivalent**	Primary	702 (42.34)	814 (37.25)	5170 (34.68)	20 (60.61)	1634 (51.53)	15 (44.12)	6866 (38.04)	48.04
	Booster	840 (50.66)	1120 (51.26)	8635 (57.92)	12 (36.36)	1313 (41.41)	19 (55.88)	9839 (54.51)	22.20
	Unknown	116 (7.00)	251 (11.49)	1103 (7.40)	1 (3.03)	224 (7.06)	NA	1344 (7.45)	NA

	Total	1658	2185	14908	33	3171	34	18049	28.50
**Spikevax Bivalent**	Primary	111 (15.86)	157 (14.51)	1489 (23.08)	4 (11.43)	668 (34.05)	5 (45.45)	2091 (24.75)	25.75
	Booster	437 (62.43)	702 (64.88)	4229 (65.56)	31 (88.57)	848 (43.22)	6 (54.55)	5174 (61.25)	15.13
	Unknown	152 (21.71)	223 (20.61)	733 (11.36)	NA	446 (22.73)	NA	1182 (13.99)	NA
	Total	700	1082	6451	35	1962	11	8447	17.17
**Monovalent mRNA vaccines**	Primary	87925 (70.51)	105601 (69.46)	577427 (68.63)	6988 (72.75)	242077 (69.87)	2698 (72.84)	848595 (68.74)	23.92
	Booster	18252 (14.64)	23999 (15.79)	155476 (18.48)	1186 (12.35)	64387 (18.58)	445 (12.01)	223836 (18.13)	29.94
	Unknown	18513 (14.85)	22430 (14.75)	108445 (12.89)	1432 (14.91)	39991 (11.54)	561 (15.15)	162129 (13.13)	NA
	Total	124690	152030	841348	9606	346455	3704	1234560	24.97
**Bivalent mRNA vaccines**	Primary	813 (34.48)	971 (29.72)	6659 (31.18)	24 (35.29)	2302 (44.85)	20 (44.44)	8957 (33.81)	39.97
	Booster	1277 (54.16)	1822 (55.77)	12864 (60.23)	43 (63.24)	2161 (42.10)	25 (55.56)	15013 (56.66)	19.12
	Unknown	268 (11.37)	474 (14.51)	1836 (8.60)	1 (1.47)	670 (13.05)	NA	2526 (9.53)	NA
	Total	2358	3267	21359	68	5133	45	26496	23.75
**All mRNA vaccines**	Primary	88738 (69.85)	106572 (68.62)	584086 (67.70)	7012 (72.48)	244379 (69.51)	2718 (72.50)	857552 (68.00)	24.03
	Booster	19529 (15.37)	25821 (16.63)	168340 (19.51)	1229 (12.70)	66548 (18.93)	470 (12.54)	238849 (18.94)	28.91
	Unknown	18781 (14.78)	22904 (14.75)	110281 (12.78)	1433 (14.81)	40661 (11.56)	561 (14.96)	164655 (13.06)	NA
	Total	127048	155297	862707	9674	351588	3749	1261056	24.94
