# Supplementary material for: Post-Marketing Safety of mRNA Vaccines: A Real-World Study Integrating Literature Case Reports and Vaccine Adverse Event Reporting System
Source: Vaccines (Basel). 2026 Jun 12;14(6):524. doi: 10.3390/vaccines14060524 (PMC13308135; doi:10.3390/vaccines14060524)
Supplement: Supplementary file 1 [file vaccines-14-00524-s001.zip › Table S12.pdf]

Table S12. Top 10 PTs Leading to SAEs during primary and booster immunization in VAERS.

SAEs	Primary immunization	Booster immunization	Primary immunization	Booster immunization	Primary immunization	Booster immunization
Vaccines	Comirnaty		Spikevax		mRESVIA	
**DIED**	Death（6701）	Death（1095）	Death（5629）	Death（845）	NA	NA
	Breakthrough COVID-19（5539）	Breakthrough COVID-19（672）	Breakthrough COVID-19（3466）	Breakthrough COVID-19（393）	NA	NA
	Dyspnoea（2061）	Dyspnoea（375）	Dyspnoea（1125）	Dyspnoea（148）	NA	NA
	Pyrexia（1228）	Pyrexia（285）	Cardiac arrest（503）	Pyrexia（111）	NA	NA
	Cardiac arrest（1099）	Cardiac arrest（256）	Pyrexia（493）	Cardiac arrest（91）	NA	NA
	Asthenia（894）	Malaise（190）	Asthenia（487）	Asthenia（76）	NA	NA
	General physical health deterioration（860）	Sudden death（185）	General physical health deterioration（481）	Condition aggravated（72）	NA	NA
	Fatigue（765）	Fatigue（175）	Pneumonia（404）	Unresponsive to stimuli（66）	NA	NA
	Pneumonia（741）	Asthenia（161）	Hypoxia（395）	Cardio-respiratory arrest（64）	NA	NA
	Cough（718）	Pulmonary embolism（147）	Cough（394）	General physical health deterioration（64）	NA	NA
**L_THREAT**	Dyspnoea（2789）	Dyspnoea（601）	Dyspnoea（1072）	Dyspnoea（209）	NA	NA
	Pulmonary embolism（1900）	Pulmonary embolism（508）	Pulmonary embolism（661）	Pulmonary embolism（203）	NA	NA
	Headache（1712）	Fatigue（392）	Pyrexia（631）	Fatigue（168）	NA	NA
	Fatigue（1673）	Chest pain（338）	Fatigue（577）	Pyrexia（144）	NA	NA

	Pyrexia（1559）	Pyrexia（324）	Headache（526）	Chest pain（130）	NA	NA
	Chest pain（1545）	Headache（282）	Chest pain（520）	Headache（117）	NA	NA
	Dizziness（1190）	Myocarditis（273）	Dizziness（400）	Cerebrovascular accident（114）	NA	NA
	Nausea（982）	Cerebrovascular accident（236）	Asthenia（399）	Nausea（80）	NA	NA
	Malaise（942）	Malaise（230）	Cerebrovascular accident（380）	Malaise（77）	NA	NA
	Myocarditis（912）	Breakthrough COVID-19（224）	Pain in extremity（360）	Pain（77）	NA	NA
**HOSPITAL**	Breakthrough COVID-19（20441）	Breakthrough COVID-19（5319）	Breakthrough COVID-19（12554）	Breakthrough COVID-19（4306）	Delivery（1）	NA
	Dyspnoea（14259）	Dyspnoea（3300）	Dyspnoea（5706）	Dyspnoea（1488）	Induced labour（1）	NA
	Pyrexia（10949）	Fatigue（2443）	Pyrexia（4441）	Pyrexia（1128）	Pre-eclampsia（1）	NA
	Headache（10615）	Pyrexia（2319）	Fatigue（3122）	Fatigue（1055）	NA	NA
	Fatigue（9738）	Headache（1966）	Headache（2880）	Headache（823）	NA	NA
	Chest pain（8364）	Chest pain（1844）	Asthenia（2788）	Asthenia（778）	NA	NA
	Dizziness（7854）	Asthenia（1679）	Chest pain（2533）	Dizziness（660）	NA	NA
	Asthenia（6923）	Dizziness（1648）	Cough（2466）	Chest pain（654）	NA	NA
	Nausea（6443）	Pulmonary embolism（1350）	Nausea（2224）	Cough（635）	NA	NA
	Malaise（5704）	Myocarditis（1312）	Dizziness（2114）	Acute respiratory failure（627）	NA	NA
**STAY**	Pyrexia（202）	Pyrexia（40）	Pyrexia（48）	Headache（4）	NA	NA
	Dyspnoea（174）	Headache（37）	Dyspnoea（35）	Dyspnoea（3）	NA	NA
	Headache（156）	Dyspnoea（34）	Headache（34）	Fatigue（2）	NA	NA

	Breakthrough COVID-19（135）	Fatigue（29）	Fatigue（32）	Myalgia（2）	NA	NA
	Fatigue（133）	Chest pain（27）	Breakthrough COVID-19（26）	Nausea（2）	NA	NA
	Dizziness（126）	Breakthrough COVID-19（26）	Dizziness（23）	Pain（2）	NA	NA
	Chest pain（112）	Malaise（20）	Nausea（21）	Palpitations（2）	NA	NA
	Vomiting（87）	Cough（19）	Chest pain（20）	Abdominal pain（1）	NA	NA
	Malaise（83）	Asthenia（18）	Chills（20）	Anaphylactic reaction（1）	NA	NA
	Palpitations（79）	Dizziness（18）	Myalgia（20）	Arthralgia（1）	NA	NA
**DISABLE**	Fatigue（9050）	Fatigue（2058）	Fatigue（2391）	Fatigue（1295）	NA	NA
	Headache（7724）	Headache（1716）	Headache（2146）	Headache（1188）	NA	NA
	Arthralgia（5497）	Arthralgia（1300）	Pyrexia（1929）	Pyrexia（968）	NA	NA
	Myalgia（5311）	Myalgia（1245）	Myalgia（1528）	Myalgia（967）	NA	NA
	Pyrexia（4565）	Pyrexia（1191）	Arthralgia（1462）	Chills（818）	NA	NA
	Pain in extremity（4066）	Pain in extremity（975）	Chills（1273）	Arthralgia（736）	NA	NA
	Nausea（3898）	Pain（847）	Nausea（1217）	Nausea（702）	NA	NA
	Dizziness（3775）	Nausea（836）	Malaise（1069）	Malaise（686）	NA	NA
	Pain（3535）	Malaise（824）	Pain in extremity（1063）	Vaccination site reaction（488）	NA	NA
	Malaise（3307）	Chills（792）	Pain（1035）	Pain in extremity（405）	NA	NA
**BIRTH_DEFECT**	Abortion spontaneous（138）	Abortion spontaneous（23）	Abortion spontaneous（48）	Abortion spontaneous（15）	Chills（1）	NA

	Foetal death（35）	Breakthrough COVID-19（6）	Pyrexia（30）	Fatigue（5）	Cough（1）	NA
	Foetal growth restriction（33）	Congenital anomaly（6）	Headache（21）	Headache（5）	Headache（1）	NA
	Headache（30）	Delivery（6）	Fatigue（20）	Nausea（3）	Myalgia（1）	NA
	Pyrexia（29）	Foetal death（6）	Pain（17）	Pregnancy（3）	Nausea（1）	NA
	Fatigue（28）	Dyspnoea（5）	Chills（14）	Pruritus（3）	Pain（1）	NA
	Dyspnoea（27）	Fatigue（5）	Dizziness（12）	Pyrexia（3）	NA	NA
	Congenital anomaly（25）	Ventricular septal defect（5）	Pain in extremity（12）	Vomiting（3）	NA	NA
	Dizziness（25）	Atrial septal defect（4）	Nausea（10）	Cerebrovascular accident（2）	NA	NA
	Stillbirth（22）	Chills（4）	Chest pain（9）	Chills（2）	NA	NA
**Vaccines**	**MNEXSPIKE**		**Comirnaty Bivalent**		**Spikevax Bivalent**	
**DIED**	Death（2）	Death（2）	Death（56）	Death（123）	Death（15）	Death（71）
	Dysphagia（2）	Dyspnoea（1）	Breakthrough COVID-19（29）	Breakthrough COVID-19（44）	Dyspnoea（5）	Breakthrough COVID-19（22）
	Amyotrophic lateral sclerosis（1）	Hyperhidrosis（1）	Dyspnoea（18）	Hypotension（19）	Acute respiratory failure（3）	Dyspnoea（12）
	Asthenia（1）	Unresponsive to stimuli（1）	General physical health deterioration（16）	Condition aggravated（17）	Breakthrough COVID-19（3）	Unresponsive to stimuli（11）
	Bulbar palsy（1）	NA	Malaise（16）	Dyspnoea（17）	Hypoxia（3）	Condition aggravated（9）
	Cardiac arrest（1）	NA	Cardiac arrest（15）	Acute respiratory failure（16）	Sudden death（3）	Asthenia（8）

	Dyspnoea（1）	NA	Asthenia（14）	Hypoxia（16）	Acute kidney injury（2）	Cardiac arrest（8）
	Gait disturbance（1）	NA	Myocardial infarction（13）	Unresponsive to stimuli（16）	Cardio-respiratory arrest（2）	Pneumonia（8）
	Head discomfort（1）	NA	Acute respiratory failure（11）	Cardiac arrest（15）	Chronic obstructive pulmonary disease（2）	Acute respiratory failure（7）
	Hypersomnia（1）	NA	Fatigue（11）	General physical health deterioration（15）	Condition aggravated（2）	General physical health deterioration（7）
**L_THREAT**	Dyspnoea（3）	Abdominal pain（1）	Dyspnoea（30）	Dyspnoea（33）	Dyspnoea（9）	Dyspnoea（24）
	Asthenia（2）	Confusional state（1）	Fatigue（27）	Pulmonary embolism（26）	Chest pain（6）	Asthenia（17）
	Confusional state（2）	Jugular vein thrombosis（1）	Pyrexia（21）	Fatigue（24）	Atrial fibrillation（4）	Atrial fibrillation（13）
	Deep vein thrombosis（2）	Loss of consciousness（1）	Pulmonary embolism（19）	Pyrexia（22）	Fatigue（4）	Fatigue（13）
	Diarrhoea（2）	Mesenteric vein thrombosis（1）	Chest pain（17）	Cerebrovascular accident（21）	Headache（4）	Headache（11）
	Dizziness（2）	Myocarditis（1）	Palpitations（15）	Condition aggravated（19）	Pyrexia（4）	Pulmonary embolism（11）
	Fatigue（2）	Pulmonary embolism（1）	Asthenia（13）	Asthenia（18）	Malaise（3）	Cerebrovascular accident（10）
	Headache（2）	Transient ischaemic attack（1）	Tachycardia（13）	Atrial fibrillation(17）	Pulmonary embolism（3）	Breakthrough COVID-19（9）
	Myocardial	NA	Headache（12）	Dizziness（17）	Tachycardia（3）	Chest pain（9）

	infarction（2）					
	Myocarditis（2）	NA	Cerebrovascular accident（11）	Malaise（15）	Vomiting（3）	Loss of consciousness（9）
**HOSPITAL**	Asthenia（4）	Tachycardia（2）	Breakthrough COVID-19（293）	Breakthrough COVID-19（752）	Breakthrough COVID-19（84）	Breakthrough COVID-19（519）
	Pyrexia（4）	Abdominal pain upper（1）	Dyspnoea（153）	Acute respiratory failure（288）	Dyspnoea（59）	Dyspnoea（125）
	Anxiety（3）	Anxiety（1）	Acute respiratory failure（150）	Hypoxia（248）	Acute respiratory failure（45）	Acute respiratory failure（99）
	Dizziness（3）	Appendicitis（1）	Cough（102）	Dyspnoea（223）	Cough（38）	Asthenia（81）
	Dyspnoea（3）	Ataxia（1）	Pyrexia（98）	Symptom recurrence（202）	Condition aggravated（34）	Hypoxia（79）
	Gait disturbance（3）	Atrial fibrillation（1）	Asthenia（94）	Asthenia（184）	Pyrexia（32）	Pyrexia（76）
	Myocardial infarction（3）	Atrial flutter（1）	Condition aggravated（94）	Hypotension（167）	Asthenia（31）	Cough（70）
	Nausea（3）	Condition aggravated（1）	Fatigue（79）	Condition aggravated（161）	Fatigue（30）	Condition aggravated（62）
	Respiratory failure（3）	Confusional state（1）	Hypoxia（69）	Cough（154）	Hypoxia（29）	Fatigue（62）
	Confusional state（2）	Dizziness（1）	Malaise（65）	Pyrexia（145）	Malaise（23）	Chest pain（48）
**X_STAY**	Pulmonary embolism（1）	NA	Cognitive disorder（2）	Breakthrough COVID-19（1）	Diarrhoea（1）	Appendix cancer（1）
	NA	NA	Confusional state（2）	Chills（1）	Pyrexia（1）	Arthritis（1）
	NA	NA	Aphasia（1）	Exercise tolerance decreased（1）	Tachycardia（1）	Bladder dysfunction（1）
	NA	NA	Atrial fibrillation（1）	Feeling abnormal（1）	Vomiting（1）	Cardiac arrest（1）

	NA	NA	Cerebral ischaemia（1）	Gait disturbance（1）	NA	Cardio-respiratory arrest（1）
	NA	NA	Circulatory collapse（1）	Loss of control of legs（1）	NA	Chronic obstructive pulmonary disease（1）
	NA	NA	General physical health deterioration（1）	Muscle tightness（1）	NA	Condition aggravated（1）
	NA	NA	Hypertension（1）	Poor quality sleep(1）	NA	Cough（1）
	NA	NA	Hypocalcaemia（1）	Sensory loss（1）	NA	Deep vein thrombosis（1）
	NA	NA	Hypokalaemia（1）	Spinal cord abscess（1）	NA	Dizziness（1）
**DISABLE**	Anxiety（4）	NA	Fatigue（66）	Tinnitus（33）	Fatigue（36）	Pain in extremity（22）
	Fatigue（4）	NA	Arthralgia（41）	Fatigue（31）	Headache（26）	Fatigue（21）
	Arthralgia（3）	NA	Pyrexia（36）	Dizziness（29）	Dyspnoea（20）	Pain（21）
	Burning sensation(3）	NA	Headache（35）	Cerebrovascular accident（23）	Pyrexia（20）	Headache（20）
	Dizziness（3）	NA	Myalgia（35）	Condition aggravated（21）	Nausea（19）	Dizziness（15）
	Gait disturbance（3）	NA	Dyspnoea（30）	Hypoaesthesia（21）	Pain in extremity（16）	Pyrexia（15）
	Muscular weakness（3）	NA	Pain（29）	Pain（21）	Palpitations（15）	Tinnitus（15）
	Nausea（3）	NA	Pain in extremity（29）	Dyspnoea（19）	Chest pain（14）	Mobility decreased（14）

	Arthritis（2）	NA	Malaise（28）	Malaise（18）	Dizziness（13）	Arthralgia（12）
	Breakthrough COVID-19（2）	NA	Palpitations（28）	Arthralgia（17）	Vaccination site pain（13）	Condition aggravated（12）
**BIRTH_DEFECT**	Abdominal distension（1）	NA	Arthralgia（1）	Pain（2）	Deep vein thrombosis（1）	Cerebral artery occlusion（1）
	Abdominal pain（1）	NA	Discomfort（1）	Arthralgia（1）	Eye disorder（1）	Fatigue（1）
	Amnesia（1）	NA	Dysmenorrhoea（1）	Bone pain（1）	Pharyngeal disorder（1）	Left-to-right cardiac shunt（1）
	Anaphylactic reaction（1）	NA	Foetal chromosome abnormality（1）	Bursitis（1）	Renal pain（1）	Muscular weakness（1）
	Anxiety（1）	NA	Foetal death（1）	Chest discomfort（1）	Urticaria（1）	Pyrexia（1）
	Arthralgia（1）	NA	Foetal hypokinesia（1）	Ectopic pregnancy with contraceptive device（1）	NA	Speech disorder（1）
	Bone pain（1）	NA	Gait disturbance（1）	Fatigue（1）	NA	NA
	Brain fog（1）	NA	Heavy menstrual bleeding（1）	Haemorrhage（1）	NA	NA
	Condition aggravated（1）	NA	Intermenstrual bleeding（1）	Hyperhidrosis（1）	NA	NA
	Confusional state（1）	NA	Menstruation irregular（1）	Joint range of motion decreased（1）	NA	NA
**Vaccines**	**Monovalent mRNA vaccines**		**Bivalent mRNA vaccines**		**All mRNA vaccines**	
**DIED**	Death（12332）	Death（1942）	Death（71）	Death（194）	Death（12403）	Death（2136）
	Breakthrough COVID-19（9005）	Breakthrough COVID-19（1065）	Breakthrough COVID-19（32）	Breakthrough COVID-19（66）	Breakthrough COVID-19（9037）	Breakthrough COVID-19（1131）
	Dyspnoea（3187）	Dyspnoea（524）	Dyspnoea（23）	Dyspnoea（29）	Dyspnoea（3210）	Dyspnoea（553）

	Pyrexia（1721）	Pyrexia（396）	General physical health deterioration（17）	Unresponsive to stimuli（27）	Pyrexia（1728）	Pyrexia（400）
	Cardiac arrest（1603）	Cardiac arrest（347）	Malaise（17）	Condition aggravated（26）	Cardiac arrest（1619）	Cardiac arrest（370）
	Asthenia（1382）	Malaise（253）	Cardiac arrest（16）	Hypotension（25）	Asthenia（1397）	Malaise（268）
	General physical health deterioration（1341）	Asthenia（237）	Asthenia（15）	Acute respiratory failure（23）	General physical health deterioration（1358）	Asthenia（258）
	Pneumonia（1145）	Fatigue（235）	Acute respiratory failure（14）	Cardiac arrest（23）	Pneumonia（1157）	Fatigue（242）
	Fatigue（1134）	Sudden death（228）	Myocardial infarction（14）	General physical health deterioration（22）	Fatigue（1146）	General physical health deterioration（231）
	Cough（1112）	General physical health deterioration（209）	Fatigue（12）	Asthenia（21）	Cough（1117）	Sudden death（229）
**L_THREAT**	Dyspnoea（3864）	Dyspnoea（810）	Dyspnoea（39）	Dyspnoea（57）	Dyspnoea（3903）	Dyspnoea（867）
	Pulmonary embolism（2562）	Pulmonary embolism（712）	Fatigue（31）	Fatigue（37）	Pulmonary embolism（2584）	Pulmonary embolism（749）
	Fatigue（2252）	Fatigue（560）	Pyrexia（25）	Pulmonary embolism（37）	Fatigue（2283）	Fatigue（597）
	Headache（2240）	Chest pain（468）	Chest pain（23）	Asthenia（35）	Headache（2256）	Pyrexia（498）
	Pyrexia（2192）	Pyrexia（468）	Pulmonary embolism（22）	Cerebrovascular accident（31）	Pyrexia（2217）	Chest pain（488）
	Chest pain（2066）	Headache（399）	Palpitations（17）	Atrial fibrillation（30）	Chest pain（2089）	Headache（423）

	Dizziness（1592）	Cerebrovascular accident（350）	Headache（16）	Pyrexia（30）	Dizziness（1598）	Cerebrovascular accident（381）
	Nausea（1331）	Myocarditis（345）	Tachycardia（16）	Condition aggravated（26）	Nausea（1338）	Myocarditis（353）
	Cerebrovascular accident（1288）	Malaise（307）	Asthenia（13）	Dizziness（25）	Cerebrovascular accident（1300）	Malaise（331）
	Asthenia（1259）	Breakthrough COVID-19（296）	Atrial fibrillation（12）	Headache（24）	Asthenia（1272）	Breakthrough COVID-19（317）
**HOSPITAL**	Breakthrough COVID-19（32996）	Breakthrough COVID-19（9625）	Breakthrough COVID-19（377）	Breakthrough COVID-19（1271）	Breakthrough COVID-19（33373）	Breakthrough COVID-19（10896）
	Dyspnoea（19968）	Dyspnoea（4788）	Dyspnoea（212）	Acute respiratory failure（387）	Dyspnoea（20180）	Dyspnoea（5136）
	Pyrexia（15394）	Fatigue（3498）	Acute respiratory failure（195）	Dyspnoea（348）	Pyrexia（15524）	Fatigue（3671）
	Headache（13497）	Pyrexia（3448）	Cough（140）	Hypoxia（327）	Headache（13542）	Pyrexia（3669）
	Fatigue（12862）	Headache（2790）	Pyrexia（130）	Asthenia（265）	Fatigue（12971）	Headache（2877）
	Chest pain（10898）	Chest pain（2498）	Condition aggravated（128）	Symptom recurrence（239）	Chest pain（10981）	Asthenia（2722）
	Dizziness（9971）	Asthenia（2457）	Asthenia（125）	Cough（224）	Dizziness（10011）	Chest pain（2612）
	Asthenia（9715）	Dizziness（2309）	Fatigue（109）	Condition aggravated（223）	Asthenia（9840）	Dizziness（2405）
	Nausea（8670）	Pulmonary embolism（1875）	Hypoxia（98）	Pyrexia（221）	Nausea（8720）	Acute respiratory failure（2165）
	Cough（7615）	Acute respiratory failure（1778）	Malaise（88）	Hypotension（214）	Cough（7755）	Pulmonary embolism（2002）
**X_STAY**	Pyrexia（250）	Headache（41）	Cognitive disorder	Appendix cancer（1）	Pyrexia（251）	Headache（42）

			（2）			
	Dyspnoea（209）	Pyrexia（41）	Confusional state（2）	Arthritis（1）	Dyspnoea（209）	Pyrexia（41）
	Headache（190）	Dyspnoea（37）	Aphasia（1）	Bladder dysfunction（1）	Headache（190）	Dyspnoea（38）
	Fatigue（165）	Fatigue（31）	Atrial fibrillation（1）	Breakthrough COVID-19（1）	Fatigue（165）	Fatigue（31）
	Breakthrough COVID-19（161）	Chest pain（28）	Cerebral ischaemia（1）	Cardiac arrest（1）	Breakthrough COVID-19（161）	Breakthrough COVID-19（28）
	Dizziness（149）	Breakthrough COVID-19（27）	Circulatory collapse（1）	Cardio-respiratory arrest（1）	Dizziness（149）	Chest pain（28）
	Chest pain（132）	Malaise（21）	Diarrhoea（1）	Chills（1）	Chest pain（132）	Malaise（22）
	Vomiting（103）	Cough（20）	General physical health deterioration（1）	Chronic obstructive pulmonary disease（1）	Vomiting（104）	Cough（21）
	Malaise（99）	Asthenia（18）	Hypertension（1）	Condition aggravated（1）	Malaise（99）	Dizziness（19）
	Nausea（88）	Dizziness（18）	Hypocalcaemia（1）	Cough（1）	Nausea（88）	Asthenia（18）
**DISABLE**	Fatigue（11445）	Fatigue（3353）	Fatigue（102）	Fatigue（52）	Fatigue（11547）	Fatigue（3405）
	Headache（9871）	Headache（2904）	Headache（61）	Tinnitus（48）	Headache（9932）	Headache（2941）
	Arthralgia（6962）	Myalgia（2212）	Pyrexia（56）	Dizziness（44）	Arthralgia（7015）	Myalgia（2231）
	Myalgia（6839）	Pyrexia（2159）	Arthralgia（53）	Pain（42）	Myalgia（6885）	Pyrexia（2188）
	Pyrexia（6495）	Arthralgia（2036）	Dyspnoea（50）	Headache（37）	Pyrexia（6551）	Arthralgia（2065）
	Pain in extremity（5131）	Chills（1610）	Myalgia（46）	Pain in extremity（36）	Pain in extremity（5176）	Chills（1625）
	Nausea（5118）	Nausea（1538）	Pain in extremity（45）	Condition aggravated（33）	Nausea（5156）	Nausea（1553）

	Dizziness（4679）	Malaise（1510）	Palpitations（43）	Cerebrovascular accident（31）	Dizziness（4718）	Malaise（1537）
	Pain（4572）	Pain in extremity（1380）	Dizziness（39）	Arthralgia（29）	Pain（4606）	Pain in extremity（1416）
	Malaise（4377）	Pain（1189）	Nausea（38）	Pyrexia（29）	Malaise（4412）	Pain（1231）
**BIRTH_DEFECT**	Abortion spontaneous（186）	Abortion spontaneous（38）	Arthralgia（1）	Fatigue（2）	Abortion spontaneous（186）	Abortion spontaneous（38）
	Pyrexia（59）	Fatigue（10）	Deep vein thrombosis（1）	Muscular weakness（2）	Pyrexia（59）	Fatigue（12）
	Headache（53）	Delivery（8）	Discomfort（1）	Pain（2）	Headache（53）	Delivery（8）
	Fatigue（49）	Headache（8）	Dysmenorrhoea（1）	Arthralgia（1）	Fatigue（49）	Headache（8）
	Foetal death（44）	Congenital anomaly（7）	Eye disorder（1）	Bone pain（1）	Foetal death（45）	Congenital anomaly（7）
	Foetal growth restriction（42）	Dyspnoea（7）	Foetal chromosome abnormality（1）	Bursitis（1）	Foetal growth restriction（42）	Dyspnoea（7）
	Pain（39）	Foetal death（7）	Foetal death（1）	Cerebral artery occlusion（1）	Pain（40）	Foetal death（7）
	Dizziness（37）	Breakthrough COVID-19（6）	Foetal hypokinesia（1）	Chest discomfort（1）	Dizziness（37）	Breakthrough COVID-19（6）
	Dyspnoea（36）	Chills（6）	Gait disturbance（1）	Ectopic pregnancy with contraceptive device（1）	Dyspnoea（36）	Chills（6）
	Chills（30）	Foetal growth restriction（6）	Heavy menstrual bleeding（1）	Haemorrhage（1）	Pain in extremity（31）	Foetal growth restriction（6）

DIED: died; L_THREAT: life threatening; HOSPITAL: hospitalized; X_STAY: prolonged hospitalization; DISABLE: disability; BIRTH_DEFECT: Congenital anomaly or birth defect.
