# Supplementary material for: Post-Marketing Safety of mRNA Vaccines: A Real-World Study Integrating Literature Case Reports and Vaccine Adverse Event Reporting System
Source: Vaccines (Basel). 2026 Jun 12;14(6):524. doi: 10.3390/vaccines14060524 (PMC13308135; doi:10.3390/vaccines14060524)
Supplement: Supplementary file 1 [file vaccines-14-00524-s001.zip › Table S13.pdf]

Table S13. Top-ranked positive PT of mRNA vaccines in literature case reports.

Vaccines	PT	n	ROR	ROR025	ROR975	IC	IC025	IC975	SOC
**Comirnaty**	Myocarditis	**198**	2.55	**2.46**	2.63	0.49	**0.45**	0.52	Cardiac disorders
	Breakthrough COVID-19	**157**	2.32	**2.30**	2.34	0.44	**0.43**	0.45	Infections and infestations
	Lymphadenopathy	**94**	1.53	1.50	1.56	0.26	0.24	0.28	Blood and lymphatic system disorders
	Myopericarditis	**62**	2.78	**2.40**	3.21	0.52	**0.38**	0.66	Cardiac disorders
	Guillain-Barre syndrome *	**52**	0.83	0.79	0.88	-0.13	-0.20	-0.06	Nervous system disorders
	Herpes zoster *	**49**	0.59	0.58	0.61	-0.40	-0.44	-0.37	Infections and infestations
	Pericarditis	**40**	3.04	**2.91**	3.17	0.55	**0.51**	0.59	Cardiac disorders
	Rash	**40**	0.62	0.61	0.63	-0.36	-0.38	-0.34	Skin and subcutaneous tissue disorders
	Immune thrombocytopenia * #	**39**	1.15	1.06	1.25	0.09	-0.01	0.20	Blood and lymphatic system disorders
	Thrombotic thrombocytopenic purpura #	**36**	1.77	1.35	2.30	0.33	0.04	0.62	Blood and lymphatic system disorders
	Autoimmune hepatitis * #	27	1.81	1.51	2.16	0.34	0.15	0.54	Hepatobiliary disorders
	Rheumatic disorder *	25	3.58	**2.90**	4.43	0.60	**0.41**	0.79	Musculoskeletal and connective tissue disorders
	Anaphylactic reaction #	23	2.12	2.03	2.22	0.41	**0.37**	0.46	Immune system disorders
	Erythema multiforme #	20	0.86	0.78	0.95	-0.11	-0.24	0.02	Skin and subcutaneous tissue disorders
	Rhabdomyolysis * #	11	1.29	1.13	1.47	0.16	0.00	0.32	Musculoskeletal and connective tissue disorders
	Angioedema #	10	1.40	1.32	1.49	0.21	0.14	0.28	Skin and subcutaneous tissue disorders
	Optic ischaemic neuropathy * #	9	1.91	1.51	2.42	0.37	0.11	0.62	Eye disorders
	Sarcoidosis *	8	2.98	**2.40**	3.71	0.54	0.34	0.75	Immune system disorders
	Pancreatitis acute * #	8	2.04	1.74	2.41	0.40	0.23	0.57	Gastrointestinal disorders
	Autoimmune haemolytic anaemia * #	7	1.58	1.25	2.00	0.28	0.01	0.54	Blood and lymphatic system disorders

	Acute kidney injury * #	7	1.09	1.03	1.16	0.06	-0.01	0.13	Renal and urinary disorders
	Deafness * #	5	1.63	1.53	1.73	0.29	0.22	0.36	Ear and labyrinth disorders
	Aplastic anaemia * #	5	1.42	1.05	1.92	0.22	-0.13	0.57	Blood and lymphatic system disorders
	Polyneuropathy *	4	2.64	**2.33**	3.00	0.50	**0.38**	0.62	Nervous system disorders
	Diabetes insipidus *	4	5.36	**2.75**	10.48	0.70	0.20	1.20	Endocrine disorders
	Pulmonary hypertension * #	4	1.20	1.01	1.44	0.12	-0.09	0.33	Respiratory, thoracic and mediastinal disorders
	Stevens-Johnson syndrome * #	4	0.98	0.74	1.28	-0.02	-0.36	0.32	Skin and subcutaneous tissue disorders
	Multisystem inflammatory syndrome in children *	3	7.15	**4.86**	10.52	0.75	**0.49**	1.01	Immune system disorders
	Cardiac failure *	3	2.45	**2.28**	2.63	0.47	**0.40**	0.54	Cardiac disorders
	Facial paralysis	3	2.05	1.97	2.14	0.40	**0.36**	0.44	Nervous system disorders
	Deafness neurosensory * #	3	1.30	1.13	1.50	0.17	0.00	0.33	Ear and labyrinth disorders
	Toxic epidermal necrolysis * #	3	1.23	0.76	1.99	0.13	-0.43	0.70	Skin and subcutaneous tissue disorders
**Spikevax**	Myocarditis	**83**	0.65	0.63	0.68	-0.45	-0.50	-0.40	Cardiac disorders
	Bacille Calmette-Guerin scar reactivation *	**37**	1.64	0.96	2.81	0.46	-0.25	1.16	Immune system disorders
	Breakthrough COVID-19	**31**	0.49	0.48	0.49	-0.77	-0.78	-0.75	Infections and infestations
	Skin reaction	**27**	2.26	**2.04**	2.49	0.71	**0.58**	0.83	Skin and subcutaneous tissue disorders
	Lymphadenopathy	**26**	0.96	0.94	0.98	-0.04	-0.07	-0.01	Blood and lymphatic system disorders
	Myopericarditis	**22**	0.43	0.36	0.51	-0.94	-1.19	-0.69	Cardiac disorders
	Guillain-Barre syndrome *	**21**	0.36	0.33	0.39	-1.16	-1.27	-1.04	Nervous system disorders
	Immune thrombocytopenia * #	**20**	0.52	0.47	0.58	-0.71	-0.87	-0.55	Blood and lymphatic system disorders
	Hypersensitivity	**19**	0.79	0.76	0.82	-0.25	-0.30	-0.19	Immune system disorders
	Haematuria *	**19**	0.78	0.70	0.87	-0.26	-0.42	-0.10	Renal and urinary disorders
	Autoimmune hepatitis * #	13	0.64	0.52	0.79	-0.47	-0.76	-0.18	Hepatobiliary disorders

	Pyrexia	12	1.35	**1.34**	1.36	0.28	**0.26**	0.29	General disorders and administration site conditions
	Vaccination site reaction	9	2.20	**2.13**	2.27	0.69	**0.65**	0.73	General disorders and administration site conditions
	Chronic spontaneous urticaria	9	3.01	**2.42**	3.75	0.90	**0.64**	1.17	Skin and subcutaneous tissue disorders
	Thrombotic thrombocytopenic purpura #	9	0.59	0.43	0.80	-0.57	-1.01	-0.13	Blood and lymphatic system disorders
	Urticaria chronic	7	2.06	**1.77**	2.40	0.64	**0.44**	0.84	Skin and subcutaneous tissue disorders
	Erythema multiforme #	7	1.20	1.08	1.33	0.17	0.03	0.32	Skin and subcutaneous tissue disorders
	Rhabdomyolysis * #	7	0.75	0.65	0.88	-0.29	-0.51	-0.08	Musculoskeletal and connective tissue disorders
	Chills	6	1.63	**1.61**	1.64	0.44	**0.43**	0.45	General disorders and administration site conditions
	Rash	6	1.36	**1.34**	1.38	0.29	**0.27**	0.31	Skin and subcutaneous tissue disorders
	Haemolysis * #	6	0.52	0.37	0.73	-0.71	-1.19	-0.23	Blood and lymphatic system disorders
	Angioedema #	5	0.77	0.72	0.83	-0.27	-0.36	-0.17	Skin and subcutaneous tissue disorders
	Anaphylactic reaction #	5	0.50	0.47	0.52	-0.76	-0.84	-0.69	Immune system disorders
	Aplastic anaemia * #	5	0.59	0.41	0.85	-0.57	-1.09	-0.05	Blood and lymphatic system disorders
	Rash erythematous	4	2.35	**2.28**	2.42	0.73	**0.69**	0.77	Skin and subcutaneous tissue disorders
	Headache	4	1.19	**1.18**	1.20	0.16	**0.15**	0.17	Nervous system disorders
	Acute kidney injury * #	4	1.04	0.97	1.10	0.03	-0.05	0.12	Renal and urinary disorders
	Autoimmune haemolytic anaemia * #	4	0.69	0.53	0.90	-0.39	-0.77	-0.01	Blood and lymphatic system disorders
	Urticaria	3	1.50	**1.47**	1.53	0.38	**0.35**	0.41	Skin and subcutaneous tissue disorders
	Stevens-Johnson syndrome * #	3	0.79	0.58	1.08	-0.25	-0.69	0.19	Skin and subcutaneous tissue disorders
**Comirnaty**	Immune thrombocytopenia #	**3**	0.61	**0.36**	1.03	-0.72	**-1.46**	0.03	Blood and lymphatic system disorders

**Bivalent**									
**Spikevax Bivalent**	Lymphadenopathy	**3**	0.50	**0.43**	0.57	-1.00	**-1.21**	-0.79	Blood and lymphatic system disorders
**Monovalent mRNA vaccines**	Myocarditis	**281**	5.20	**4.82**	5.61	0.21	**0.18**	0.24	Cardiac disorders
	Breakthrough COVID-19	**188**	1.73	1.71	1.75	0.10	**0.09**	0.11	Infections and infestations
	Lymphadenopathy	**120**	2.57	**2.48**	2.66	0.16	**0.14**	0.17	Blood and lymphatic system disorders
	Myopericarditis	**84**	2.17	1.73	2.71	0.14	0.00	0.27	Cardiac disorders
	Guillain-Barre syndrome	**73**	0.33	0.31	0.34	-0.43	-0.49	-0.36	Nervous system disorders
	Immune thrombocytopenia #	**59**	0.60	0.54	0.66	-0.15	-0.25	-0.06	Blood and lymphatic system disorders
	Bacille Calmette-Guerin scar reactivation	**55**	1.03	0.50	2.10	0.01	-0.56	0.58	Immune system disorders
	Herpes zoster	**53**	0.26	0.25	0.27	-0.56	-0.59	-0.53	Infections and infestations
	Pericarditis	**52**	3.98	**3.66**	4.32	0.19	**0.16**	0.23	Cardiac disorders
	Hypersensitivity	**46**	0.82	0.78	0.86	-0.05	-0.09	-0.01	Immune system disorders
	Thrombotic thrombocytopenic purpura #	45	1.33	0.92	1.93	0.06	-0.21	0.33	Blood and lymphatic system disorders
	Autoimmune hepatitis #	40	1.60	1.23	2.09	0.09	-0.09	0.27	Hepatobiliary disorders
	Anaphylactic reaction #	28	1.51	1.43	1.61	0.08	0.04	0.13	Immune system disorders
	Erythema multiforme #	27	1.00	0.88	1.15	0.00	-0.11	0.11	Skin and subcutaneous tissue disorders
	Rhabdomyolysis #	18	1.04	0.87	1.25	0.01	-0.13	0.15	Musculoskeletal and connective tissue disorders
	Angioedema #	15	1.28	1.17	1.39	0.05	-0.01	0.11	Skin and subcutaneous tissue disorders
	Acute kidney injury #	11	1.25	1.14	1.35	0.05	-0.01	0.11	Renal and urinary disorders
	Autoimmune haemolytic anaemia #	11	1.36	0.97	1.90	0.06	-0.18	0.31	Blood and lymphatic system disorders
	Aplastic anaemia #	10	0.90	0.62	1.33	-0.03	-0.35	0.30	Blood and lymphatic system disorders
	Optic ischaemic neuropathy #	9	1.64	1.15	2.33	0.10	-0.14	0.33	Eye disorders

	Pancreatitis acute #	8	2.16	1.65	2.82	0.14	-0.02	0.30	Gastrointestinal disorders
	Chest pain	7	1.83	1.78	1.88	0.11	**0.10**	0.13	General disorders and administration site conditions
	Urticaria chronic	7	2.68	**1.99**	3.62	0.16	0.00	0.32	Skin and subcutaneous tissue disorders
	Haemolysis #	7	1.18	0.81	1.70	0.04	-0.25	0.32	Blood and lymphatic system disorders
	Stevens-Johnson syndrome #	7	0.70	0.51	0.98	-0.10	-0.40	0.20	Skin and subcutaneous tissue disorders
	Deafness #	5	1.33	1.21	1.45	0.06	-0.01	0.13	Ear and labyrinth disorders
	Toxic epidermal necrolysis #	5	0.67	0.38	1.17	-0.12	-0.64	0.41	Skin and subcutaneous tissue disorders
	Hypertensive crisis	4	3.80	**3.14**	4.60	0.19	**0.10**	0.28	Vascular disorders
	Deafness neurosensory #	4	1.14	0.94	1.39	0.03	-0.12	0.18	Ear and labyrinth disorders
	Pulmonary hypertension #	4	1.13	0.89	1.45	0.03	-0.16	0.22	Respiratory, thoracic and mediastinal disorders
	Drug reaction with eosinophilia and systemic symptoms #	4	1.11	0.66	1.86	0.02	-0.38	0.43	Skin and subcutaneous tissue disorders
	Tachycardia	3	2.20	**2.10**	2.30	0.14	**0.11**	0.16	Cardiac disorders
	Angina pectoris	3	2.60	**2.39**	2.82	0.16	**0.11**	0.20	Cardiac disorders
	Pericardial effusion	3	2.66	**2.34**	3.04	0.16	**0.09**	0.23	Cardiac disorders
	Cardiac failure	3	2.31	**2.06**	2.59	0.14	**0.08**	0.21	Cardiac disorders
	Thrombophlebitis	3	3.51	**2.62**	4.71	0.18	0.04	0.32	Vascular disorders
	Dermatitis exfoliative generalised #	3	1.26	0.81	1.96	0.05	-0.28	0.38	Skin and subcutaneous tissue disorders
**Bivalent mRNA vaccines**	Lymphadenopathy	**5**	0.54	**0.50**	0.59	-0.86	**-1.00**	-0.73	Blood and lymphatic system disorders
	Immune thrombocytopenia #	**3**	0.44	**0.28**	0.70	-1.17	**-1.84**	-0.51	Blood and lymphatic system disorders
**All mRNA vaccines**	Myocarditis	**282**	5.22	**4.81**	5.66	0.18	**0.15**	0.21	Cardiac disorders
	Breakthrough COVID-19	**188**	2.08	2.05	2.12	0.11	**0.11**	0.12	Infections and infestations
	Lymphadenopathy	**125**	2.63	**2.53**	2.73	0.14	**0.12**	0.16	Blood and lymphatic system disorders

Myopericarditis	**84**	2.36	1.85	3.02	0.13	0.00	0.26	Cardiac disorders
Guillain-Barre syndrome	**73**	0.30	0.28	0.32	-0.43	-0.50	-0.37	Nervous system disorders
Immune thrombocytopenia #	**62**	0.54	0.49	0.60	-0.17	-0.26	-0.07	Blood and lymphatic system disorders
Bacille Calmette-Guerin scar reactivation	**55**	1.20	0.54	2.64	0.04	-0.53	0.60	Immune system disorders
Herpes zoster	**53**	0.23	0.23	0.24	-0.57	-0.60	-0.54	Infections and infestations
Pericarditis	**52**	4.18	**3.82**	4.58	0.17	**0.14**	0.21	Cardiac disorders
Hypersensitivity	**46**	0.77	0.74	0.81	-0.06	-0.10	-0.02	Immune system disorders
Thrombotic thrombocytopenic purpura #	45	1.24	0.85	1.82	0.04	-0.23	0.31	Blood and lymphatic system disorders
Autoimmune hepatitis #	40	1.59	1.20	2.11	0.08	-0.10	0.26	Hepatobiliary disorders
Anaphylactic reaction #	28	1.38	1.30	1.47	0.06	0.02	0.10	Immune system disorders
Erythema multiforme #	27	0.92	0.80	1.06	-0.02	-0.13	0.09	Skin and subcutaneous tissue disorders
Rhabdomyolysis #	18	1.12	0.93	1.36	0.02	-0.12	0.17	Musculoskeletal and connective tissue disorders
Angioedema #	15	1.21	1.11	1.32	0.04	-0.02	0.10	Skin and subcutaneous tissue disorders
Acute kidney injury #	11	1.51	1.37	1.66	0.07	0.01	0.14	Renal and urinary disorders
Autoimmune haemolytic anaemia #	11	1.25	0.88	1.76	0.04	-0.20	0.29	Blood and lymphatic system disorders
Aplastic anaemia #	10	0.96	0.64	1.45	-0.01	-0.33	0.31	Blood and lymphatic system disorders
Optic ischaemic neuropathy #	9	2.06	1.37	3.09	0.11	-0.12	0.35	Eye disorders
Pancreatitis acute #	8	2.35	1.75	3.16	0.13	-0.03	0.29	Gastrointestinal disorders
Chest pain	7	1.88	1.82	1.93	0.10	**0.09**	0.12	General disorders and administration site conditions
Urticaria chronic	7	3.54	**2.47**	5.07	0.16	0.00	0.32	Skin and subcutaneous tissue disorders
Haemolysis #	7	1.10	0.75	1.60	0.02	-0.26	0.30	Blood and lymphatic system disorders
Stevens-Johnson syndrome #	7	0.65	0.46	0.90	-0.11	-0.41	0.19	Skin and subcutaneous tissue disorders

Ventricular tachycardia	6	3.56	**2.58**	4.91	0.16	0.02	0.31	Cardiac disorders
Deafness #	5	1.30	1.18	1.43	0.05	-0.02	0.12	Ear and labyrinth disorders
Toxic epidermal necrolysis #	5	0.58	0.33	1.01	-0.15	-0.67	0.37	Skin and subcutaneous tissue disorders
Hypertensive crisis	4	3.88	**3.16**	4.77	0.17	**0.08**	0.26	Vascular disorders
Deafness neurosensory #	4	1.21	0.98	1.50	0.04	-0.11	0.19	Ear and labyrinth disorders
Pulmonary hypertension #	4	1.52	1.14	2.04	0.08	-0.11	0.26	Respiratory, thoracic and mediastinal disorders
Drug reaction with eosinophilia and systemic symptoms #	4	1.21	0.69	2.11	0.04	-0.36	0.44	Skin and subcutaneous tissue disorders
Angina pectoris	3	2.81	**2.56**	3.08	0.14	**0.10**	0.19	Cardiac disorders
Tachycardia	3	2.26	2.16	2.38	0.12	**0.10**	0.15	Cardiac disorders
Pericardial effusion	3	3.08	**2.66**	3.58	0.15	**0.08**	0.22	Cardiac disorders
Cardiac failure	3	2.83	**2.47**	3.23	0.15	**0.08**	0.21	Cardiac disorders
Thrombophlebitis	3	3.19	**2.36**	4.30	0.15	0.01	0.30	Vascular disorders
Dermatitis exfoliative generalised #	3	1.15	0.73	1.80	0.03	-0.30	0.35	Skin and subcutaneous tissue disorders

PT: Preferred Terms; n: Number of case report; N: Number of VAERS reports; ROR: reporting odds ratio of VAERS reports; ROR_{025}: Lower limit of the 95% CI for ROR of VAERS reports; ROR_{975}: Upper limit of the 95% CI for ROR of VAERS reports; IC: Information component of VAERS reports; IC025: Lower limit of the 95% credible interval for IC of VAERS reports; IC975: Upper limit of the 95% credible interval for IC of VAERS reports; SOC: System Organ Class. In PT column: ① # indicates PTs within designated medical events (DME) list; ② * indicates signals not listed in the product labeling. In other columns, values in bold indicate that the PT ranks among the top 10 highest values based on n, ROR_{025}, or IC−2SD.
