# Supplementary material for: Post-Marketing Safety of mRNA Vaccines: A Real-World Study Integrating Literature Case Reports and Vaccine Adverse Event Reporting System
Source: Vaccines (Basel). 2026 Jun 12;14(6):524. doi: 10.3390/vaccines14060524 (PMC13308135; doi:10.3390/vaccines14060524)
Supplement: Supplementary file 1 [file vaccines-14-00524-s001.zip › Table S14.pdf]

Table S14. Demographic characteristics of SAEs in literature case reports.

SAEs	Characteristic	Comirnaty	Spikevax	Comirnaty Bivalent	Spikevax Bivalent	Monovalent mRNA vaccines	Bivalent mRNA vaccines	All mRNA vaccines
**DIED**	Age (n (%))							
	Median	71	67	NA	NA	70	NA	70
	< 6 months	NA	NA	NA	NA	NA	NA	NA
	6 months-11 years	1 (1.10)	NA	NA	NA	1 (0.79)	NA	1 (0.79)
	12-17 years	2 (2.20)	NA	NA	NA	2 (1.59)	NA	2 (1.59)
	18-44 years	14 (15.38)	9 (25.71)	NA	NA	23 (18.25)	NA	23 (18.25)
	45-64 years	19 (20.88)	3 (8.57)	NA	NA	22 (17.46)	NA	22 (17.46)
	≥65 years	53 (58.24)	23 (65.71)	NA	NA	76 (60.32)	NA	76 (60.32)
	Unknown	2 (2.20)	NA	NA	NA	2 (1.59)	NA	2 (1.59)
	Sex (n (%))							
	F	42 (46.15)	10 (28.57)	NA	NA	52 (41.27)	NA	52 (41.27)
	M	41 (45.05)	24 (68.57)	NA	NA	65 (51.59)	NA	65 (51.59)
	Unknown	8 (8.79)	1 (2.86)	NA	NA	9 (7.14)	NA	9 (7.14)
	History (n (%))							
	No history	5 (5.49)	1 (2.86)	NA	NA	6 (4.76)	NA	6 (4.76)
	With history	61 (67.03)	22 (62.86)	NA	NA	83 (65.87)	NA	83 (65.87)
	Unknown	25 (27.47)	12 (34.29)	NA	NA	37 (29.37)	NA	37 (29.37)
	Total	91	35	NA	NA	126	NA	126
**L_THREAT**	Age (n (%))							
	Median	52	51	NA	NA	51	NA	51
	< 6 months	NA	NA	NA	NA	NA	NA	NA
	6 months-11 years	NA	NA	NA	NA	NA	NA	NA
	12-17 years	4 (4.88)	3 (1.38)	NA	NA	7 (2.34)	NA	7 (2.34)

	18-44 years	29 (35.37)	79 (36.41)	NA	NA	108 (36.12)	NA	108 (36.12)
	45-64 years	25 (30.49)	63 (29.03)	NA	NA	88 (29.43)	NA	88 (29.43)
	≥65 years	24 (29.27)	69 (31.80)	NA	NA	93 (31.10)	NA	93 (31.10)
	Unknown	NA	3 (1.38)	NA	NA	3 (1.00)	NA	3 (1.00)
	Sex (n (%))							
	F	44 (53.66)	97 (44.70)	NA	NA	141 (47.16)	NA	141 (47.16)
	M	36 (43.90)	120 (55.30)	NA	NA	156 (52.17)	NA	156 (52.17)
	Unknown	2 (2.44)	NA	NA	NA	2 (0.67)	NA	2 (0.67)
	History (n (%))							
	No history	2 (2.44)	27 (12.44)	NA	NA	29 (9.70)	NA	29 (9.70)
	With history	63 (76.83)	130 (59.91)	NA	NA	193 (64.55)	NA	193 (64.55)
	Unknown	17 (20.73)	60 (27.65)	NA	NA	77 (25.75)	NA	77 (25.75)
	Total	82	217	NA	NA	299	NA	299
**HOSPITAL**	Age (n (%))							
	Median	47	50	81	47	48	75	48
	< 6 months	NA	NA	NA	NA	NA	NA	NA
	6 months-11 years	12 (1.18)	NA	NA	NA	12 (0.80)	NA	12 (0.79)
	12-17 years	121 (11.92)	5 (1.02)	NA	NA	126 (8.37)	NA	126 (8.32)
	18-44 years	335 (33.00)	188 (38.29)	NA	NA	523 (34.73)	NA	523 (34.52)
	45-64 years	264 (26.01)	148 (30.14)	NA	2 (66.67)	412 (27.36)	2 (22.22)	414 (27.33)
	≥65 years	271 (26.70)	148 (30.14)	5 (83.33)	1 (33.33)	419 (27.82)	6 (66.67)	425 (28.05)
	Unknown	12 (1.18)	2 (0.41)	1 (16.67)	NA	14 (0.93)	1 (11.11)	15 (0.99)
	Sex (n (%))							
	F	511 (50.34)	207 (42.16)	3 (50.00)	NA	718 (47.68)	3 (33.33)	721 (47.59)
	M	476 (46.90)	280 (57.03)	3 (50.00)	3 (100.00)	756 (50.20)	6 (66.67)	762 (50.30)
	Unknown	28 (2.76)	4 (0.81)	NA	NA	32 (2.12)	NA	32 (2.11)

	History (n (%))							
	No history	222 (21.87)	33 (6.72)	NA	2 (66.67)	255 (16.93)	2 (22.22)	257 (16.96)
	With history	524 (51.63)	299 (60.90)	5 (83.33)	1 (33.33)	823 (54.65)	6 (66.67)	829 (54.72)
	Unknown	269 (26.50)	159 (32.38)	1 (16.67)	NA	428 (28.42)	1 (11.11)	429 (28.32)
	Total	1015	491	6	3	1506	9	1515
**X_STAY**	Age (n (%))							
	Median	54	60	NA	NA	59	NA	59
	< 6 months	NA	NA	NA	NA	NA	NA	NA
	6 months-11 years	NA	NA	NA	NA	NA	NA	NA
	12-17 years	NA	2 (2.22)	NA	NA	2 (1.68)	NA	2 (1.68)
	18-44 years	10 (34.48)	24 (26.67)	NA	NA	34 (28.57)	NA	34 (28.57)
	45-64 years	13 (44.83)	27 (30.00)	NA	NA	40 (33.61)	NA	40 (33.61)
	≥65 years	6 (20.69)	37 (41.11)	NA	NA	43 (36.13)	NA	43 (36.13)
	Unknown	NA	NA	NA	NA	NA	NA	NA
	Sex (n (%))							
	F	13 (44.83)	39 (43.33)	NA	NA	52 (43.70)	NA	52 (43.70)
	M	16 (55.17)	51 (56.67)	NA	NA	67 (56.30)	NA	67 (56.30)
	Unknown	NA	NA	NA	NA	NA	NA	NA
	History (n (%))							
	No history	3 (10.34)	7 (7.78)	NA	NA	10 (8.40)	NA	10 (8.40)
	With history	22 (75.86)	58 (64.44)	NA	NA	80 (67.23)	NA	80 (67.23)
	Unknown	4 (13.79)	25 (27.78)	NA	NA	29 (24.37)	NA	29 (24.37)
	Total	29	90	NA	NA	119	NA	119
**DISABLE**	Age (n (%))							
	Median	42	62	NA	NA	52	NA	52
	< 6 months	NA	NA	NA	NA	NA	NA	NA

	6 months-11 years	NA	NA	NA	NA	NA	NA	NA
	12-17 years	3 (4.76)	1 (1.02)	NA	NA	4 (2.48)	NA	4 (2.48)
	18-44 years	31 (49.21)	27 (27.55)	NA	NA	58 (36.02)	NA	58 (36.02)
	45-64 years	13 (20.63)	26 (26.53)	NA	NA	39 (24.22)	NA	39 (24.22)
	≥65 years	16 (25.40)	44 (44.90)	NA	NA	60 (37.27)	NA	60 (37.27)
	Unknown	NA	NA	NA	NA	NA	NA	NA
	Sex (n (%))							
	F	27 (42.86)	43 (43.88)	NA	NA	70 (43.48)	NA	70 (43.48)
	M	36 (57.14)	55 (56.12)	NA	NA	91 (56.52)	NA	91 (56.52)
	Unknown	NA	NA	NA	NA	NA	NA	NA
	History (n (%))							
	No history	3 (4.76)	5 (5.10)	NA	NA	8 (4.97)	NA	8 (4.97)
	With history	34 (53.97)	63 (64.29)	NA	NA	97 (60.25)	NA	97 (60.25)
	Unknown	26 (41.27)	30 (30.61)	NA	NA	56 (34.78)	NA	56 (34.78)
	Total	63	98	NA	NA	161	NA	161
**BIRTH_DEFECT**	Age (n (%))							
	Median	34	NA	NA	NA	34	NA	34
	< 6 months	NA	NA	NA	NA	NA	NA	NA
	6 months-11 years	NA	NA	NA	NA	NA	NA	NA
	12-17 years	NA	NA	NA	NA	NA	NA	NA
	18-44 years	1 (100.00)	NA	NA	NA	1 (100.00)	NA	1 (100.00)
	45-64 years	NA	NA	NA	NA	NA	NA	NA
	≥65 years	NA	NA	NA	NA	NA	NA	NA
	Unknown	NA	NA	NA	NA	NA	NA	NA
	Sex (n (%))							
	F	1 (100.00)	NA	NA	NA	1 (100.00)	NA	1 (100.00)

	M	NA	NA	NA	NA	NA	NA	NA
	Unknown	NA	NA	NA	NA	NA	NA	NA
	History (n (%))							
	No history	NA	NA	NA	NA	NA	NA	NA
	With history	1 (100.00)	NA	NA	NA	1 (100.00)	NA	1 (100.00)
	Unknown	NA	NA	NA	NA	NA	NA	NA
	Total	1	NA	NA	NA	1	NA	1
**All SAEs**	Age (n (%))							
	Median	47	51	81	47	48	75	48
	< 6 months	NA	NA	NA	NA	NA	NA	NA
	6 months-11 years	12 (1.10)	NA	NA	NA	12 (0.73)	NA	12 (0.73)
	12-17 years	127 (11.67)	6 (1.10)	NA	NA	133 (8.14)	NA	133 (8.10)
	18-44 years	363 (33.36)	207 (37.98)	NA	NA	570 (34.91)	NA	570 (34.71)
	45-64 years	278 (25.55)	159 (29.17)	NA	2 (66.67)	437 (26.76)	2 (22.22)	439 (26.74)
	≥65 years	296 (27.21)	168 (30.83)	5 (83.33)	1 (33.33)	464 (28.41)	6 (66.67)	470 (28.62)
	Unknown	12 (1.10)	5 (0.92)	1 (16.67)	NA	17 (1.04)	1 (11.11)	18 (1.10)
	Sex (n (%))							
	F	540 (49.63)	234 (42.94)	3 (50.00)	NA	774 (47.40)	3 (33.33)	777 (47.32)
	M	515 (47.33)	307 (56.33)	3 (50.00)	3 (100.00)	822 (50.34)	6 (66.67)	828 (50.43)
	Unknown	33 (3.03)	4 (0.73)	NA	NA	37 (2.27)	NA	37 (2.25)
	History (n (%))							
	No history	226 (20.77)	41 (7.52)	NA	2 (66.67)	267 (16.35)	2 (22.22)	269 (16.38)
	With history	564 (51.84)	326 (59.82)	5 (83.33)	1 (33.33)	890 (54.50)	6 (66.67)	896 (54.57)
	Unknown	298 (27.39)	178 (32.66)	1 (16.67)	NA	476 (29.15)	1 (11.11)	477 (29.05)
	Total	1088	545	6	3	1633	9	1642
