# Supplementary material for: Post-Marketing Safety of mRNA Vaccines: A Real-World Study Integrating Literature Case Reports and Vaccine Adverse Event Reporting System
Source: Vaccines (Basel). 2026 Jun 12;14(6):524. doi: 10.3390/vaccines14060524 (PMC13308135; doi:10.3390/vaccines14060524)
Supplement: Supplementary file 1 [file vaccines-14-00524-s001.zip › Table S15.pdf]

Table S15. Top 10 PTs Leading to SAEs in literature case reports.

Vaccines	DIED	L_THREAT	HOSPITAL	X_STAY	DISABLE	BIRTH_DEFECT	All SAEs
**Comirnaty**	Breakthrough COVID-19（13）	Myocarditis（5）	Myocarditis（77）	Myopericarditis（3）	Myocarditis（12）	Tachycardia foetal（1）	Myocarditis（87）
	Myocarditis（6）	Autoimmune hepatitis（5）	Breakthrough COVID-19（52）	Anxiety（2）	Guillain-Barre syndrome（6）	NA	Breakthrough COVID-19（61）
	Encephalitis autoimmune（4）	Glomerulonephritis minimal lesion（4）	Myopericarditis（37）	Autoimmune hepatitis（2）	Central serous chorioretinopathy（4）	NA	Myopericarditis（38）
	Interstitial lung disease（4）	Guillain-Barre syndrome（4）	Guillain-Barre syndrome（23）	Cerebral venous thrombosis（2）	Glomerulonephritis minimal lesion（3）	NA	Guillain-Barre syndrome（27）
	Encephalitis（3）	Breakthrough COVID-19（3）	Immune thrombocytopenia（18）	Viral pericarditis（1）	Myelitis transverse（2）	NA	Immune thrombocytopenia（18）
	Pulmonary embolism（3）	Immune thrombocytopenia（3）	Thrombotic thrombocytopenic purpura（18）	Breakthrough COVID-19（1）	Dermatomyositis（2）	NA	Thrombotic thrombocytopenic purpura（18）
	Status epilepticus（3）	Cerebral venous thrombosis（3）	Autoimmune hepatitis（15）	Colitis ulcerative（1）	Anaphylactic reaction（2）	NA	Autoimmune hepatitis（16）
	Autoimmune hepatitis（2）	Myopericarditis（3）	Multisystem inflammatory syndrome（15）	Cytokine release syndrome（1）	Autoimmune haemolytic anaemia（2）	NA	Multisystem inflammatory syndrome（16）
	Inflammation（2）	Hypersensitivity	Pneumonitis（15）	Capillary leak	Uveitis（1）	NA	Pneumonitis

		（2）		syndrome（1）			（15）
	Acquired haemophilia（2）	Acquired haemophilia（2）	Interstitial lung disease（13）	Antiphospholipid syndrome（1）	Colitis ulcerative（1）	NA	Pericarditis（14）
**Spikevax**	Thrombosis with thrombocytopenia syndrome（5）	Myocarditis（22）	Myocarditis（60）	Immune thrombocytopenia（5）	Myocarditis（8）	NA	Myocarditis（62）
	Systemic lupus erythematosus（2）	Immune thrombocytopenia（8）	Myopericarditis（18）	Pericarditis（4）	Guillain-Barre syndrome（5）	NA	Myopericarditis（18）
	Interstitial lung disease（2）	Myopericarditis（5）	Breakthrough COVID-19（14）	Myocarditis（4）	Encephalitis（4）	NA	Breakthrough COVID-19（16）
	Thrombotic thrombocytopenic purpura（2）	Pericarditis（5）	Immune thrombocytopenia（13）	Encephalitis（4）	Retinal artery occlusion（3）	NA	Immune thrombocytopenia（14）
	Acquired haemophilia（2）	Encephalitis（5）	Guillain-Barre syndrome（12）	Breakthrough COVID-19（4）	Neuralgic amyotrophy（3）	NA	Guillain-Barre syndrome（13）
	Hypertensive crisis（1）	Breakthrough COVID-19（5）	Encephalitis（10）	Systemic lupus erythematosus（3）	Breakthrough COVID-19（3）	NA	Encephalitis（11）
	Lichen planus（1）	Henoch-Schonlein purpura（4）	Henoch-Schonlein purpura（8）	Acquired haemophilia(3）	Autoimmune hepatitis（3）	NA	Henoch-Schonlein purpura（9）
	Chronic inflammatory demyelinating	Guillain-Barre syndrome（4）	Pericarditis（8）	Aplastic anaemia（2）	Chronic inflammatory demyelinating	NA	Pericarditis（8）

	polyradiculoneuropathy（1）				polyradiculoneuropathy（2）		
	Acute respiratory distress syndrome（1）	Glomerulonephritis（4）	Pulmonary embolism（7）	Guillain-Barre syndrome（2）	Anti-neutrophil cytoplasmic antibody positive vasculitis（2）	NA	Vasculitis（8）
	Parkinsonism（1）	Stress cardiomyopathy（4）	Encephalopathy（7）	Multisystem inflammatory syndrome（2）	Optic neuritis（2）	NA	Thrombotic thrombocytopenic purpura(7)
**Comirnaty Bivalent**	NA	NA	Cerebral venous sinus thrombosis（1）	NA	NA	NA	Cerebral venous sinus thrombosis（1）
	NA	NA	Myocarditis（1）	NA	NA	NA	Myocarditis（1）
	NA	NA	Ventricular tachycardia（1）	NA	NA	NA	Ventricular tachycardia（1）
	NA	NA	Lymphadenopathy（1）	NA	NA	NA	Lymphadenopathy（1）
	NA	NA	Idiopathic pulmonary fibrosis（1）	NA	NA	NA	Idiopathic pulmonary fibrosis（1）
	NA	NA	Colitis（1）	NA	NA	NA	Colitis（1）
	NA	NA	NA	NA	NA	NA	NA
	NA	NA	NA	NA	NA	NA	NA
	NA	NA	NA	NA	NA	NA	NA
	NA	NA	NA	NA	NA	NA	NA

**Spikevax Bivalent**	NA	NA	Encephalitis autoimmune（1）	NA	NA	NA	Encephalitis autoimmune（1）
	NA	NA	Mania（1）	NA	NA	NA	Mania（1）
	NA	NA	Psychotic disorder（1）	NA	NA	NA	Psychotic disorder（1）
	NA	NA	NA	NA	NA	NA	NA
	NA	NA	NA	NA	NA	NA	NA
	NA	NA	NA	NA	NA	NA	NA
	NA	NA	NA	NA	NA	NA	NA
	NA	NA	NA	NA	NA	NA	NA
	NA	NA	NA	NA	NA	NA	NA
	NA	NA	NA	NA	NA	NA	NA
**Monovalent mRNA vaccines**	Breakthrough COVID-19（13）	Myocarditis（27）	Myocarditis（137）	Immune thrombocytopenia（6）	Myocarditis（20）	Tachycardia foetal（1）	Myocarditis（149）
	Myocarditis（7）	Immune thrombocytopenia（11）	Breakthrough COVID-19（66）	Breakthrough COVID-19（5）	Guillain-Barre syndrome（11）	NA	Breakthrough COVID-19（77）
	Interstitial lung disease（6）	Autoimmune hepatitis（9）	Myopericarditis（55）	Autoimmune hepatitis（4）	Myelitis transverse（4）	NA	Myopericarditis（56）
	Thrombosis with thrombocytopenia syndrome（5）	Breakthrough COVID-19（8）	Guillain-Barre syndrome（35）	Pericarditis（4）	Glomerulonephritis minimal lesion（4）	NA	Guillain-Barre syndrome（40）
	Encephalitis（4）	Myopericarditis（8）	Immune thrombocytopeni	Myocarditis（4）	Central serous chorioretinopathy	NA	Immune thrombocytope

			a（31）		（4）		nia（32）
	Encephalitis autoimmune（4）	Guillain-Barre syndrome（8）	Thrombotic thrombocytopenic purpura（24）	Encephalitis（4）	Encephalitis（4）	NA	Thrombotic thrombocytopenic purpura（25）
	Acquired haemophilia（4）	Acquired haemophilia（5）	Autoimmune hepatitis（21）	Cerebral venous thrombosis（3）	Dermatomyositis（3）	NA	Autoimmune hepatitis（23）
	Thrombotic thrombocytopenic purpura（4）	Glomerulonephritis minimal lesion（5）	Pericarditis（20）	Aplastic anaemia（3）	Hyperglycaemic crisis（3）	NA	Pericarditis（22）
	Pericarditis（3）	Myelitis transverse（5）	Multisystem inflammatory syndrome（20）	Myopericarditis（3）	Myopericarditis（3）	NA	Encephalitis（22）
	Guillain-Barre syndrome（3）	Vasculitis（5）	Encephalitis（19）	Hyperglycaemic crisis（3）	Optic neuritis（3）	NA	Multisystem inflammatory syndrome（21）
**Bivalent mRNA vaccines**	NA	NA	Cerebral venous sinus thrombosis（1）	NA	NA	NA	Cerebral venous sinus thrombosis（1）
	NA	NA	Myocarditis（1）	NA	NA	NA	Myocarditis（1）
	NA	NA	Ventricular tachycardia（1）	NA	NA	NA	Ventricular tachycardia（1）
	NA	NA	Lymphadenopathy（1）	NA	NA	NA	Lymphadenopathy（1）

	NA	NA	Idiopathic pulmonary fibrosis（1）	NA	NA	NA	Idiopathic pulmonary fibrosis（1）
	NA	NA	Colitis（1）	NA	NA	NA	Colitis（1）
	NA	NA	Encephalitis autoimmune（1）	NA	NA	NA	Encephalitis autoimmune（1）
	NA	NA	Mania（1）	NA	NA	NA	Mania（1）
	NA	NA	Psychotic disorder（1）	NA	NA	NA	Psychotic disorder（1）
	NA	NA	NA	NA	NA	NA	NA
**All mRNA vaccines**	Breakthrough COVID-19（13）	Myocarditis（27）	Myocarditis（138）	Immune thrombocytopenia（6）	Myocarditis（20）	Tachycardia foetal（1）	Myocarditis（150）
	Myocarditis（7）	Immune thrombocytopenia（11）	Breakthrough COVID-19（66）	Breakthrough COVID-19（5）	Guillain-Barre syndrome（11）	NA	Breakthrough COVID-19（77）
	Interstitial lung disease（6）	Autoimmune hepatitis（9）	Myopericarditis（55）	Autoimmune hepatitis（4）	Myelitis transverse（4）	NA	Myopericarditis（56）
	Thrombosis with thrombocytopenia syndrome（5）	Breakthrough COVID-19（8）	Guillain-Barre syndrome（35）	Pericarditis（4）	Glomerulonephritis minimal lesion（4）	NA	Guillain-Barre syndrome（40）
	Encephalitis（4）	Myopericarditis（8）	Immune thrombocytopenia（31）	Myocarditis（4）	Central serous chorioretinopathy（4）	NA	Immune thrombocytopenia（32）
	Encephalitis	Guillain-Barre	Thrombotic	Encephalitis（4）	Encephalitis（4）	NA	Thrombotic

autoimmune（4）	syndrome（8）	thrombocytopenic purpura（24）				thrombocytopenic purpura（25）
Acquired haemophilia（4）	Acquired haemophilia（5）	Autoimmune hepatitis（21）	Cerebral venous thrombosis（3）	Dermatomyositis（3）	NA	Autoimmune hepatitis（23）
Thrombotic thrombocytopenic purpura（4）	Glomerulonephritis minimal lesion（5）	Pericarditis（20）	Aplastic anaemia（3）	Hyperglycaemic crisis（3）	NA	Pericarditis（22）
Pericarditis（3）	Myelitis transverse（5）	Multisystem inflammatory syndrome（20）	Myopericarditis（3）	Myopericarditis（3）	NA	Encephalitis（22）
Guillain-Barre syndrome（3）	Vasculitis（5）	Encephalitis（19）	Hyperglycaemic crisis（3）	Optic neuritis（3）	NA	Multisystem inflammatory syndrome（21）

DIED: died; L_THREAT: life threatening; HOSPITAL: hospitalized; X_STAY: prolonged hospitalization; DISABLE: disability; BIRTH_DEFECT: Congenital anomaly or birth defect.
