# Supplementary material for: Post-Marketing Safety of mRNA Vaccines: A Real-World Study Integrating Literature Case Reports and Vaccine Adverse Event Reporting System
Source: Vaccines (Basel). 2026 Jun 12;14(6):524. doi: 10.3390/vaccines14060524 (PMC13308135; doi:10.3390/vaccines14060524)
Supplement: Supplementary file 1 [file vaccines-14-00524-s001.zip › Table S16.pdf]

Table S16. Immunization schedules and SAEs analysis in literature case reports.

Vaccines	Immunization schedule	DIED (n (%))	L_THREAT (n (%))	HOSPITAL (n (%))	X_STAY (n (%))	DISABLE (n (%))	BIRTH_DEFECT (n (%))	All SAEs (n (%))	SAE (%)
**Comirnaty**	Primary	65 (71.43%)	73 (89.02%)	776 (76.38%)	24 (82.76%)	57 (90.48%)	1 (100.00%)	838 (76.95%)	32.31
	Booster	12 (13.19%)	3 (3.66%)	160 (15.75%)	5 (17.24%)	3 (4.76%)	NA	164 (15.06%)	44.09
	Unknown	14 (15.38%)	6 (7.32%)	80 (7.87%)	NA	3 (4.76%)	NA	87 (7.99%)	NA
	Total	91	82	1016	29	63	1	1089	33.78
**Spikevax**	Primary	23 (65.71%)	173 (79.72%)	379 (77.19%)	65 (72.22%)	79 (80.61%)	NA	426 (78.17%)	46.56
	Booster	5 (14.29%)	30 (13.82%)	78 (15.89%)	14 (15.56%)	11 (11.22%)	NA	84 (15.41%)	44.92
	Unknown	7 (20.00%)	14 (6.45%)	34 (6.92%)	11 (12.22%)	8 (8.16%)	NA	35 (6.42%)	NA
	Total	35	217	491	90	98	NA	545	46.28
**Comirnaty Bivalent**	Booster	NA	NA	5 (100.00%)	NA	NA	NA	5 (100.00%)	50.00
	Total	NA	NA	5	NA	NA	NA	5	50.00
**Spikevax Bivalent**	Booster	NA	NA	3 (100.00%)	NA	NA	NA	3 (100.00%)	42.86
	Total	NA	NA	3	NA	NA	NA	3	42.86
**Monovalent mRNA vaccines**	Primary	88 (69.84%)	246 (82.27%)	1155 (76.64%)	89 (74.79%)	136 (84.47%)	1 (100.00%)	1264 (77.36%)	36.02
	Booster	17 (13.49%)	33 (11.04%)	238 (15.79%)	19 (15.97%)	14 (8.70%)	NA	248 (15.18%)	44.36
	Unknown	21 (16.67%)	20 (6.69%)	114 (7.56%)	11 (9.24%)	11 (6.83%)	NA	122 (7.47%)	NA
	Total	126	299	1507	119	161	1	1634	37.17
**Bivalent**	Booster	NA	NA	8 (100.00%)	NA	NA	NA	8 (100.00%)	47.06

**mRNA vaccines**	Total	NA	NA	8	NA	NA	NA	8	47.06
**All mRNA vaccines**	Primary	88 (69.84)	246 (82.27)	1155 (76.24)	89 (74.79)	136 (84.47)	1 (100.00)	1264 (76.98)	36.02
	Booster	17 (13.49)	33 (11.04)	246 (16.24)	19 (15.97)	14 (8.7)	NA	256 (15.59)	44.44
	Unknown	21 (16.67)	20 (6.69)	114 (7.52)	11 (9.24)	11 (6.83)	NA	122 (7.43)	NA
	Total	126	299	1515	119	161	1	1642	37.21
