# Supplementary material for: Post-Marketing Safety of mRNA Vaccines: A Real-World Study Integrating Literature Case Reports and Vaccine Adverse Event Reporting System
Source: Vaccines (Basel). 2026 Jun 12;14(6):524. doi: 10.3390/vaccines14060524 (PMC13308135; doi:10.3390/vaccines14060524)
Supplement: Supplementary file 1 [file vaccines-14-00524-s001.zip › Table S17.pdf]

Table S17. Top 10 PTs Leading to SAEs during primary and booster immunization in literature case reports.

SAEs	Primary immunization	Booster immunization	Primary immunization	Booster immunization	Primary immunization	Booster immunization	Primary immunization	Booster immunization
**Vaccines**	**Comirnaty**		**Spikevax**		**Comirnaty Bivalent**		**Spikevax Bivalent**	
**DIED**	Breakthrough COVID-19（10）	Breakthrough COVID-19（2）	Thrombosis with thrombocytopenia syndrome（5）	Basal ganglia haemorrhage（1）	NA	NA	NA	NA
	Interstitial lung disease（3）	Diabetes mellitus（2）	Acquired haemophilia（2）	Chronic inflammatory demyelinating polyradiculoneuropathy（1）	NA	NA	NA	NA
	Myocarditis（3）	Idiopathic interstitial pneumonia（2）	Systemic lupus erythematosus（2）	Encephalitis（1）	NA	NA	NA	NA
	Pulmonary embolism（3）	Myocarditis（2）	Thrombotic thrombocytopenic purpura（2）	Guillain-Barre syndrome（1）	NA	NA	NA	NA
	Status epilepticus（3）	Dermatomyositis（1）	Acute respiratory distress syndrome（1）	Interstitial lung disease（1）	NA	NA	NA	NA
	Acquired haemophilia（2）	Encephalitis（1）	Cerebral venous thrombosis（1）	NA	NA	NA	NA	NA
	Autoimmune hepatitis（2）	Pancreatitis necrotising（1）	Encephalopathy（1）	NA	NA	NA	NA	NA
	Encephalitis（2）	Pericarditis（1）	Eosinophilia（1）	NA	NA	NA	NA	NA

	Guillain-Barre syndrome（2）	NA	Haemorrhage（1）	NA	NA	NA	NA	NA
	Myopericarditis（2）	NA	Lichen planus（1）	NA	NA	NA	NA	NA
**L_THREAT**	Autoimmune hepatitis（5）	Cronkhite-Canada syndrome（1）	Myocarditis（22）	Acute kidney injury（1）	NA	NA	NA	NA
	Myocarditis（5）	Dilated cardiomyopathy（1）	Breakthrough COVID-19（5）	Anhidrosis（1）	NA	NA	NA	NA
	Glomerulonephritis minimal lesion（4）	Pneumonitis（1）	Immune thrombocytopenia（5）	Antisynthetase syndrome（1）	NA	NA	NA	NA
	Breakthrough COVID-19（3）	NA	Autoimmune hepatitis（4）	Asthenia（1）	NA	NA	NA	NA
	Cerebral venous thrombosis（3）	NA	Encephalitis（4）	Brugada syndrome（1）	NA	NA	NA	NA
	Guillain-Barre syndrome（3）	NA	Glomerulonephritis（4）	Cerebral venous thrombosis（1）	NA	NA	NA	NA
	Myopericarditis（3）	NA	Myopericarditis（4）	Chronic inflammatory demyelinating polyradiculoneuropathy（1）	NA	NA	NA	NA
	Acquired haemophilia（2）	NA	Pericarditis（4）	Coronary artery	NA	NA	NA	NA

				thrombosis（1）				
	Anaphylactic reaction（2）	NA	Vasculitis（4）	Cronkhite-Canada syndrome（1）	NA	NA	NA	NA
	Autoimmune haemolytic anaemia（2）	NA	Acquired haemophilia（3）	Diabetes mellitus（1）	NA	NA	NA	NA
**HOSPITAL**	Myocarditis（70）	Breakthrough COVID-19（19）	Myocarditis（58）	Breakthrough COVID-19（4）	NA	Colitis（1）	NA	Encephalitis autoimmune（1）
	Myopericarditis（36）	Myocarditis（6）	Myopericarditis（15）	Angioedema（2）	NA	Idiopathic pulmonary fibrosis（1）	NA	Mania（1）
	Breakthrough COVID-19（26）	Pemphigoid（4）	Guillain-Barre syndrome（9）	Anhidrosis（2）	NA	Lymphadenopathy（1）	NA	Psychotic disorder（1）
	Guillain-Barre syndrome（20）	Pericarditis（4）	Breakthrough COVID-19（8）	Colitis ulcerative（2）	NA	Myocarditis（1）	NA	NA
	Thrombotic thrombocytopenic purpura（16）	Anxiety（3）	Immune thrombocytopenia（8）	Encephalitis（2）	NA	Ventricular tachycardia（1）	NA	NA
	Multisystem inflammatory syndrome（14）	Asthma（3）	Encephalitis（7）	Guillain-Barre syndrome（2）	NA	NA	NA	NA
	Pneumonitis（13）	Colitis ulcerative（3）	Henoch-Schonlein purpura（7）	Immune thrombocytopenia（2）	NA	NA	NA	NA

	Autoimmune hepatitis（12）	Diabetes mellitus（3）	Autoimmune hepatitis（6）	Myopericarditis（2）	NA	NA	NA	NA
	Immune thrombocytopenia（11）	Encephalitis（3）	Encephalopathy（6）	Pericarditis（2）	NA	NA	NA	NA
	Interstitial lung disease（11）	Guillain-Barre syndrome（3）	Pericarditis（6）	Pulmonary embolism（2）	NA	NA	NA	NA
**X_STAY**	Myopericarditis（3）	Anxiety（2）	Encephalitis（4）	Asthenia（1）	NA	NA	NA	NA
	Autoimmune hepatitis（2）	Cronkhite-Canada syndrome（1）	Acquired haemophilia（3）	Breakthrough COVID-19（1）	NA	NA	NA	NA
	Cerebral venous thrombosis（2）	Thrombotic thrombocytopenic purpura（1）	Breakthrough COVID-19（3）	Cerebral venous thrombosis（1）	NA	NA	NA	NA
	Acute kidney injury（1）	Viral pericarditis（1）	Immune thrombocytopenia（3）	Chronic inflammatory demyelinating polyradiculoneuropathy（1）	NA	NA	NA	NA
	Anaphylactic reaction（1）	NA	Myocarditis（3）	Cronkhite-Canada syndrome（1）	NA	NA	NA	NA
	Antiphospholipid syndrome（1）	NA	Pericarditis（3）	Epilepsy（1）	NA	NA	NA	NA
	Aplastic anaemia（1）	NA	Aplastic anaemia（2）	Guillain-Barre syndrome（1）	NA	NA	NA	NA

	Breakthrough COVID-19（1）	NA	Autoimmune hepatitis（2）	Hypoaesthesia（1）	NA	NA	NA	NA
	Capillary leak syndrome（1）	NA	Hyperglycaemic crisis（2）	Immune thrombocytopenia（1）	NA	NA	NA	NA
	Colitis ulcerative（1）	NA	Systemic lupus erythematosus（2）	Interstitial lung disease（1）	NA	NA	NA	NA
**DISABLE**	Myocarditis（12）	Cronkhite-Canada syndrome（1）	Myocarditis（8）	Cerebral venous thrombosis（1）	NA	NA	NA	NA
	Guillain-Barre syndrome（5）	Optic neuritis（1）	Autoimmune hepatitis（3）	Chronic inflammatory demyelinating polyradiculoneuropathy（1）	NA	NA	NA	NA
	Central serous chorioretinopathy（4）	Pneumonitis（1）	Breakthrough COVID-19（3）	Cronkhite-Canada syndrome（1）	NA	NA	NA	NA
	Glomerulonephritis minimal lesion（3）	NA	Encephalitis（3）	Diabetes mellitus（1）	NA	NA	NA	NA
	Anaphylactic reaction（2）	NA	Guillain-Barre syndrome（3）	Encephalitis（1）	NA	NA	NA	NA
	Autoimmune haemolytic	NA	Retinal artery occlusion（3）	Epilepsy（1）	NA	NA	NA	NA

	anaemia（2）							
	Dermatomyositis（2）	NA	Acute myocardial infarction（2）	Guillain-Barre syndrome（1）	NA	NA	NA	NA
	Acute kidney injury（1）	NA	Anti-neutrophil cytoplasmic antibody positive vasculitis（2）	Interstitial lung disease（1）	NA	NA	NA	NA
	Adrenal insufficiency（1）	NA	Chronic obstructive pulmonary disease（2）	Miller Fisher syndrome（1）	NA	NA	NA	NA
	Anaphylactoid reaction（1）	NA	Hyperglycaemic crisis（2）	Neuralgic amyotrophy（1）	NA	NA	NA	NA
**BIRTH_ DEFECT**	Tachycardia foetal（1）	NA	NA	NA	NA	NA	NA	NA

**Vaccines**	**Monovalent mRNA vaccines**		**Bivalent mRNA vaccines**		**All mRNA vaccines**	
**DIED**	Breakthrough COVID-19（10）	Breakthrough COVID-19（2）	NA	NA	Breakthrough COVID-19（10）	Breakthrough COVID-19（2）
	Thrombosis with thrombocytopenia syndrome（5）	Diabetes mellitus（2）	NA	NA	Thrombosis with thrombocytopenia syndrome（5）	Diabetes mellitus（2）
	Acquired haemophilia（4）	Encephalitis（2）	NA	NA	Acquired haemophilia（4）	Encephalitis（2）
	Thrombotic	Idiopathic	NA	NA	Thrombotic thrombocytopenic	Idiopathic interstitial pneumonia

	thrombocytopenic purpura（4）	interstitial pneumonia（2）			purpura（4）	（2）
	Interstitial lung disease（3）	Myocarditis（2）	NA	NA	Interstitial lung disease（3）	Myocarditis（2）
	Myocarditis（3）	Basal ganglia haemorrhage(1）	NA	NA	Myocarditis（3）	Basal ganglia haemorrhage（1）
	Pulmonary embolism（3）	Chronic inflammatory demyelinating polyradiculoneuropathy（1）	NA	NA	Pulmonary embolism（3）	Chronic inflammatory demyelinating polyradiculoneuropathy（1）
	Status epilepticus（3）	Dermatomyositis（1）	NA	NA	Status epilepticus（3）	Dermatomyositis（1）
	Autoimmune hepatitis（2）	Guillain-Barre syndrome（1）	NA	NA	Autoimmune hepatitis（2）	Guillain-Barre syndrome（1）
	Cerebral venous thrombosis（2）	Interstitial lung disease（1）	NA	NA	Cerebral venous thrombosis（2）	Interstitial lung disease（1）
**L_THREAT**	Myocarditis(27）	Cronkhite-Canada syndrome(2）	NA	NA	Myocarditis（27）	Cronkhite-Canada syndrome（2）
	Autoimmune hepatitis（9）	Acute kidney injury（1）	NA	NA	Autoimmune hepatitis（9）	Acute kidney injury（1）
	Breakthrough COVID-19（8）	Anhidrosis（1）	NA	NA	Breakthrough COVID-19（8）	Anhidrosis（1）
	Myopericarditis（7）	Antisynthetase syndrome（1）	NA	NA	Myopericarditis（7）	Antisynthetase syndrome（1）
	Immune	Asthenia（1）	NA	NA	Immune thrombocytopenia（6）	Asthenia（1）

	thrombocytopenia（6）					
	Acquired haemophilia（5）	Brugada syndrome（1）	NA	NA	Acquired haemophilia（5）	Brugada syndrome（1）
	Glomerulonephritis minimal lesion（5）	Cerebral venous thrombosis（1）	NA	NA	Glomerulonephritis minimal lesion（5）	Cerebral venous thrombosis（1）
	Guillain-Barre syndrome（5）	Chronic inflammatory demyelinating polyradiculoneuropathy（1）	NA	NA	Guillain-Barre syndrome（5）	Chronic inflammatory demyelinating polyradiculoneuropathy（1）
	Vasculitis（5）	Coronary artery thrombosis（1）	NA	NA	Vasculitis（5）	Coronary artery thrombosis（1）
	Aplastic anaemia（4）	Diabetes mellitus（1）	NA	NA	Aplastic anaemia（4）	Diabetes mellitus（1）
**HOSPITAL**	Myocarditis（128）	Breakthrough COVID-19（23）	NA	Colitis（1）	Myocarditis（128）	Breakthrough COVID-19（23）
	Myopericarditis（51）	Myocarditis（7）	NA	Encephalitis autoimmune（1）	Myopericarditis（51）	Myocarditis（8）
	Breakthrough COVID-19（34）	Pericarditis（6）	NA	Idiopathic pulmonary fibrosis（1）	Breakthrough COVID-19（34）	Pericarditis（6）
	Guillain-Barre syndrome（29）	Colitis ulcerative（5）	NA	Lymphadenopathy（1）	Guillain-Barre syndrome（29）	Colitis ulcerative（5）

	Thrombotic thrombocytopenic purpura（21）	Encephalitis（5）	NA	Mania（1）	Thrombotic thrombocytopenic purpura（21）	Encephalitis（5）
	Immune thrombocytopenia（19）	Guillain-Barre syndrome（5）	NA	Myocarditis（1）	Immune thrombocytopenia（19）	Guillain-Barre syndrome（5）
	Autoimmune hepatitis（18）	Pemphigoid（5）	NA	Psychotic disorder（1）	Autoimmune hepatitis（18）	Pemphigoid（5）
	Multisystem inflammatory syndrome（17）	Diabetes mellitus（4）	NA	Ventricular tachycardia（1）	Multisystem inflammatory syndrome（17）	Diabetes mellitus（4）
	Thrombosis（15）	Immune thrombocytopenia（4）	NA	NA	Thrombosis（15）	Immune thrombocytopenia（4）
	Acquired haemophilia（14）	Myasthenia gravis（4）	NA	NA	Acquired haemophilia（14）	Lymphadenopathy（4）
**X_STAY**	Autoimmune hepatitis（4）	Anxiety（2）	NA	NA	Autoimmune hepatitis（4）	Anxiety（2）
	Breakthrough COVID-19（4）	Cronkhite-Canada syndrome（2）	NA	NA	Breakthrough COVID-19（4）	Cronkhite-Canada syndrome（2）
	Encephalitis（4）	Asthenia（1）	NA	NA	Encephalitis（4）	Asthenia（1）
	Immune thrombocytopenia（4）	Breakthrough COVID-19（1）	NA	NA	Immune thrombocytopenia（4）	Breakthrough COVID-19（1）
	Acquired	Cerebral venous	NA	NA	Acquired haemophilia（3）	Cerebral venous thrombosis（1）

	haemophilia（3）	thrombosis（1）				
	Aplastic anaemia（3）	Chronic inflammatory demyelinating polyradiculoneuropathy（1）	NA	NA	Aplastic anaemia（3）	Chronic inflammatory demyelinating polyradiculoneuropathy（1）
	Hyperglycaemic crisis（3）	Epilepsy（1）	NA	NA	Hyperglycaemic crisis（3）	Epilepsy（1）
	Myocarditis（3）	Guillain-Barre syndrome（1）	NA	NA	Myocarditis（3）	Guillain-Barre syndrome（1）
	Myopericarditis（3）	Hypoaesthesia（1）	NA	NA	Myopericarditis（3）	Hypoaesthesia（1）
	Pericarditis（3）	Immune thrombocytopenia（1）	NA	NA	Pericarditis（3）	Immune thrombocytopenia（1）
**DISABLE**	Myocarditis(20）	Cronkhite-Canada syndrome(2）	NA	NA	Myocarditis（20）	Cronkhite-Canada syndrome（2）
	Guillain-Barre syndrome（8）	Cerebral venous thrombosis（1）	NA	NA	Guillain-Barre syndrome（8）	Cerebral venous thrombosis（1）
	Central serous chorioretinopathy（4）	Chronic inflammatory demyelinating polyradiculoneuropathy（1）	NA	NA	Central serous chorioretinopathy（4）	Chronic inflammatory demyelinating polyradiculoneuropathy（1）
	Glomerulonephritis minimal	Diabetes mellitus（1）	NA	NA	Glomerulonephritis minimal lesion（4）	Diabetes mellitus（1）

	lesion（4）					
	Autoimmune hepatitis（3）	Encephalitis（1）	NA	NA	Autoimmune hepatitis（3）	Encephalitis（1）
	Breakthrough COVID-19（3）	Epilepsy（1）	NA	NA	Breakthrough COVID-19（3）	Epilepsy（1）
	Dermatomyositis（3）	Guillain-Barre syndrome（1）	NA	NA	Dermatomyositis（3）	Guillain-Barre syndrome（1）
	Encephalitis（3）	Interstitial lung disease（1）	NA	NA	Encephalitis（3）	Interstitial lung disease（1）
	Hyperglycaemic crisis（3）	Miller Fisher syndrome（1）	NA	NA	Hyperglycaemic crisis（3）	Miller Fisher syndrome（1）
	Myopericarditis（3）	Neuralgic amyotrophy（1）	NA	NA	Myopericarditis（3）	Neuralgic amyotrophy（1）
**BIRTH_ DEFECT**	Tachycardia foetal（1）	NA	NA	NA	Tachycardia foetal（1）	NA

DIED: died; L_THREAT: life threatening; HOSPITAL: hospitalized; X_STAY: prolonged hospitalization; DISABLE: disability; BIRTH_DEFECT: Congenital anomaly or birth defect.
