# Supplementary material for: Post-Marketing Safety of mRNA Vaccines: A Real-World Study Integrating Literature Case Reports and Vaccine Adverse Event Reporting System
Source: Vaccines (Basel). 2026 Jun 12;14(6):524. doi: 10.3390/vaccines14060524 (PMC13308135; doi:10.3390/vaccines14060524)
Supplement: Supplementary file 1 [file vaccines-14-00524-s001.zip › Table S18.pdf]

Table S18. The age-sex stratified analysis of myocarditis and pericarditis following mRNA vaccines

Characteristic		Comirnaty		Spikevax		MNEXSPIKE		Comirnaty Bivalent		Spikevax Bivalent		Monovalent mRNA vaccines		Bivalent mRNA vaccines		All mRNA COVID-19 vaccines	
**SEX**		**F**	**M**	**F**	**M**	**F**	**M**	**F**	**M**	**F**	**M**	**F**	**M**	**F**	**M**	**F**	**M**
									**VAERS**								
**Myocarditis**	Median	40	24	41	28	52	55	54	42	56	42	40	27	54	42	40	27
	< 6 months	NA	1 (0.01)	NA	NA	NA	NA	NA	NA	NA	NA	NA	1 (0.01)	NA	NA	NA	1 (0.01)
	6 months - 11 years	8 (0.17)	27 (0.32)	NA	4 (0.14)	NA	NA	NA	NA	1 (5.88)	NA	8 (0.14)	31 (0.28)	1 (2.33)	NA	9 (0.16)	31 (0.27)
	12 - 17 years	149 (3.18)	902 (10.80)	13 (1.20)	129 (4.47)	NA	NA	NA	4 (14.29)	NA	3 (18.75)	162 (2.81)	1031 (9.17)	NA	7 (15.91)	162 (2.79)	1038 (9.20)
	18 - 44 years	846 (18.07)	1643 (19.67)	534 (49.49)	1963 (68.02)	1 (50.00)	NA	2 (7.69)	5 (17.86)	3 (17.65)	5 (31.25)	1381 (23.96)	3606 (32.08)	5 (11.63)	10 (22.73)	1386 (23.87)	3616 (32.04)
	45 - 64 years	492 (10.51)	434 (5.20)	283 (26.23)	382 (13.24)	NA	2 (66.67)	10 (38.46)	4 (14.29)	4 (23.53)	4 (25.00)	775 (13.45)	818 (7.28)	14 (32.56)	8 (18.18)	789 (13.59)	826 (7.32)
	≥65 years	202 (4.31)	211 (2.53)	140 (12.97)	123 (4.26)	1 (50.00)	NA	1 (3.85)	4 (14.29)	6 (35.29)	4 (25.00)	343 (5.95)	334 (2.97)	7 (16.28)	8 (18.18)	350 (6.03)	342 (3.03)

	Unknown	2985 (63.75)	5135 (61.47)	109 (10.10)	285 (9.88)	NA	1 (33.33)	13 (50.00)	11 (39.29)	3 (17.65)	NA	3094 (53.69)	5421 (48.22)	16 (37.21)	11 (25.00)	3110 (53.57)	5432 (48.13)
	Total	4682	8353	1079	2886	2	3	26	28	17	16	5763	11242	43	44	5806	11286
**Myocarditis & Death**	Median	67	59	56	51	NA	NA	59	54	NA	46	65	56	59	46	65	56
	< 6 months	NA	NA	NA	NA	NA	NA	NA	NA	NA	NA	NA	NA	NA	NA	NA	NA
	6 months - 11 years	1 (0.91)	2 (0.99)	NA	NA	NA	NA	NA	NA	NA	NA	1 (0.69)	2 (0.73)	NA	NA	1 (0.68)	2 (0.72)
	12 - 17 years	2 (1.82)	5 (2.46)	NA	2 (2.86)	NA	NA	NA	NA	NA	NA	2 (1.38)	7 (2.56)	NA	NA	2 (1.36)	7 (2.54)
	18 - 44 years	10 (9.09)	25 (12.32)	13 (37.14)	27 (38.57)	NA	NA	NA	1 (50.00)	NA	NA	23 (15.86)	52 (19.05)	NA	1 (33.33)	23 (15.65)	53 (19.20)
	45 - 64 years	10 (9.09)	13 (6.40)	6 (17.14)	17 (24.29)	NA	NA	1 (50.00)	NA	NA	1 (100.00)	16 (11.03)	30 (10.99)	1 (50.00)	1 (33.33)	17 (11.56)	31 (11.23)
	≥65 years	30 (27.27)	34 (16.75)	15 (42.86)	19 (27.14)	NA	NA	NA	1 (50.00)	NA	NA	45 (31.03)	53 (19.41)	NA	1 (33.33)	45 (30.61)	54 (19.57)
	Unknown	57 (51.82)	124 (61.08)	1 (2.86)	5 (7.14)	NA	NA	1 (50.00)	NA	NA	NA	58 (40.00)	129 (47.25)	1 (50.00)	NA	59 (40.14)	129 (46.74)
	Total	110	203	35	70	NA	NA	2	2	NA	1	145	273	2	3	147	276
**Pericarditis**	Median	43	31	45	38	72	82	62	38	47	53	44	34	62	40	45	35

	< 6 months	1 (0.02)	NA	NA	NA	NA	NA	NA	NA	NA	NA	1 (0.02)	NA	NA	NA	1 (0.02)	NA
	6 months - 11 years	1 (0.02)	13 (0.27)	2 (0.21)	1 (0.08)	NA	NA	NA	NA	1 (7.14)	NA	3 (0.06)	14 (0.23)	1 (2.00)	NA	4 (0.07)	14 (0.23)
	12 - 17 years	54 (1.21)	232 (4.87)	10 (1.07)	22 (1.81)	NA	NA	NA	1 (2.94)	NA	1 (7.14)	64 (1.18)	254 (4.25)	NA	2 (4.17)	64 (1.17)	256 (4.25)
	18 - 44 years	618 (13.83)	732 (15.37)	415 (44.58)	672 (55.40)	NA	NA	2 (5.56)	12 (35.29)	5 (35.71)	5 (35.71)	1033 (19.13)	1404 (23.50)	7 (14.00)	17 (35.42)	1040 (19.08)	1421 (23.59)
	45 - 64 years	474 (10.61)	278 (5.84)	320 (34.37)	291 (23.99)	1 (50.00)	NA	13 (36.11)	6 (17.65)	2 (14.29)	3 (21.43)	795 (14.72)	569 (9.52)	15 (30.00)	9 (18.75)	810 (14.86)	578 (9.60)
	≥65 years	159 (3.56)	182 (3.82)	130 (13.96)	140 (11.54)	1 (50.00)	1 (100.00)	9 (25.00)	5 (14.71)	5 (35.71)	4 (28.57)	290 (5.37)	323 (5.41)	14 (28.00)	9 (18.75)	304 (5.58)	332 (5.51)
	Unknown	3161 (70.75)	3324 (69.82)	54 (5.80)	87 (7.17)	NA	NA	12 (33.33)	10 (29.41)	1 (7.14)	1 (7.14)	3215 (59.53)	3411 (57.09)	13 (26.00)	11 (22.92)	3228 (59.22)	3422 (56.82)
	Total	4468	4761	931	1213	2	1	36	34	14	14	5401	5975	50	48	5451	6023
**Pericarditis & Death**	Median	68	72	76	59	NA	NA	84	NA	NA	NA	72	69	84	NA	73	69
	< 6 months	NA	NA	NA	NA	NA	NA	NA	NA	NA	NA	NA	NA	NA	NA	NA	NA
	6 months - 11 years	NA	NA	NA	NA	NA	NA	NA	NA	NA	NA	NA	NA	NA	NA	NA	NA

	12 - 17 years	NA	NA	NA	NA	NA	NA	NA	NA	NA	NA	NA	NA	NA	NA	NA	NA
	18 - 44 years	1 (5.26)	3 (6.52)	NA	2 (20.00)	NA	NA	NA	NA	NA	NA	1 (4.35)	5 (8.93)	NA	NA	1 (4.17)	5 (8.93)
	45 - 64 years	2 (10.53)	1 (2.17)	NA	3 (30.00)	NA	NA	NA	NA	NA	NA	2 (8.70)	4 (7.14)	NA	NA	2 (8.33)	4 (7.14)
	≥65 years	7 (36.84)	12 (26.09)	4 (100.00)	4 (40.00)	NA	NA	1 (100.00)	NA	NA	NA	11 (47.83)	16 (28.57)	1 (100.00)	NA	12 (50.00)	16 (28.57)
	Unknown	9 (47.37)	30 (65.22)	NA	1 (10.00)	NA	NA	NA	NA	NA	NA	9 (39.13)	31 (55.36)	NA	NA	9 (37.50)	31 (55.36)
	Total	19	46	4	10	NA	NA	1	NA	NA	NA	23	56	1	NA	24	56
**Myocarditis and Pericarditis**	Median	42	27	43	31	66	63	58	39	54	45	42	28	57	41	42	29
	< 6 months	1 (0.01)	1 (0.01)	NA	NA	NA	NA	NA	NA	NA	NA	1 (0.01)	1 (0.01)	NA	NA	1 (0.01)	1 (0.01)
	6 months - 11 years	9 (0.10)	40 (0.31)	2 (0.10)	5 (0.12)	NA	NA	NA	NA	2 (6.45)	NA	11 (0.10)	45 (0.26)	2 (2.15)	NA	13 (0.12)	45 (0.26)
	12 - 17 years	203 (2.22)	1134 (8.65)	23 (1.14)	151 (3.68)	NA	NA	NA	5 (8.06)	NA	4 (13.33)	226 (2.02)	1285 (7.46)	NA	9 (9.78)	226 (2.01)	1294 (7.48)
	18 - 44	1464	2375	949	2635	1	NA	4	17	8	10	2414	5010	12	27	2426	5037

	years	(16.00)	(18.11)	(47.21)	(64.28)	(25.00)		(6.45)	(27.42)	(25.81)	(33.33)	(21.62)	(29.10)	(12.90)	(29.35)	(21.55)	(29.10)
	45 - 64 years	966 (10.56)	712 (5.43)	603 (30.00)	673 (16.42)	1 (25.00)	2 (50.00)	23 (37.10)	10 (16.13)	6 (19.35)	7 (23.33)	1570 (14.06)	1387 (8.06)	29 (31.18)	17 (18.48)	1599 (14.20)	1404 (8.11)
	≥65 years	361 (3.95)	393 (3.00)	270 (13.43)	263 (6.42)	2 (50.00)	1 (25.00)	10 (16.13)	9 (14.52)	11 (35.48)	8 (26.67)	633 (5.67)	657 (3.82)	21 (22.58)	17 (18.48)	654 (5.81)	674 (3.89)
	Unknown	6146 (67.17)	8459 (64.50)	163 (8.11)	372 (9.08)	NA	1 (25.00)	25 (40.32)	21 (33.87)	4 (12.90)	1 (3.33)	6309 (56.51)	8832 (51.30)	29 (31.18)	22 (23.91)	6338 (56.30)	8854 (51.15)
	Total	9150	13114	2010	4099	4	4	62	62	31	30	11164	17217	93	92	11257	17309
**Myocarditis and Pericarditis & Death**	Median	67	64	64	55	NA	NA	72	54	NA	46	67	57	72	46	67	57
	< 6 months	NA	NA	NA	NA	NA	NA	NA	NA	NA	NA	NA	NA	NA	NA	NA	NA
	6 months - 11 years	1 (0.78)	2 (0.80)	NA	NA	NA	NA	NA	NA	NA	NA	1 (0.60)	2 (0.61)	NA	NA	1 (0.58)	2 (0.60)
	12 - 17 years	2 (1.55)	5 (2.01)	NA	2 (2.50)	NA	NA	NA	NA	NA	NA	2 (1.19)	7 (2.13)	NA	NA	2 (1.17)	7 (2.11)
	18 - 44 years	11 (8.53)	28 (11.24)	13 (33.33)	29 (36.25)	NA	NA	NA	1 (50.00)	NA	NA	24 (14.29)	57 (17.33)	NA	1 (33.33)	24 (14.04)	58 (17.47)
	45 - 64 years	12 (9.30)	14 (5.62)	6 (15.38)	20 (25.00)	NA	NA	1 (33.33)	NA	NA	1 (100.00)	18 (10.71)	34 (10.33)	1 (33.33)	1 (33.33)	19 (11.11)	35 (10.54)

	≥65 years	37 (28.68)	46 (18.47)	19 (48.72)	23 (28.75)	NA	NA	1 (33.33)	1 (50.00)	NA	NA	56 (33.33)	69 (20.97)	1 (33.33)	1 (33.33)	57 (33.33)	70 (21.08)
	Unknown	66 (51.16)	154 (61.85)	1 (2.56)	6 (7.50)	NA	NA	1 (33.33)	NA	NA	NA	67 (39.88)	160 (48.63)	1 (33.33)	NA	68 (39.77)	160 (48.19)
	Total	129	249	39	80	NA	NA	3	2	NA	1	168	329	3	3	171	332
**Literature Case Reports**																	
**Myocarditis**	Median	31	21	42	23	NA	NA	NA	81	NA	NA	37	22	NA	81	37	22
	< 6 months	NA	NA	NA	NA	NA	NA	NA	NA	NA	NA	NA	NA	NA	NA	NA	NA
	6 months - 11 years	3 (7.32)	2 (1.32)	1 (4.55)	3 (4.92)	NA	NA	NA	NA	NA	NA	4 (6.35)	5 (2.35)	NA	NA	4 (6.35)	5 (2.34)
	12 - 17 years	10 (24.39)	52 (34.21)	2 (9.09)	3 (4.92)	NA	NA	NA	NA	NA	NA	12 (19.05)	55 (25.82)	NA	NA	12 (19.05)	55 (25.70)
	18 - 44 years	15 (36.59)	78 (51.32)	8 (36.36)	47 (77.05)	NA	NA	NA	NA	NA	NA	23 (36.51)	125 (58.69)	NA	NA	23 (36.51)	125 (58.41)
	45 - 64 years	10 (24.39)	12 (7.89)	9 (40.91)	8 (13.11)	NA	NA	NA	NA	NA	NA	19 (30.16)	20 (9.39)	NA	NA	19 (30.16)	20 (9.35)
	≥65 years	3 (7.32)	5 (3.29)	2 (9.09)	NA	NA	NA	NA	1 (100.00)	NA	NA	5 (7.94)	5 (2.35)	NA	1 (100.00)	5 (7.94)	6 (2.80)
	Unknown	NA	3	NA	NA	NA	NA	NA	NA	NA	NA	NA	3	NA	NA	NA	3

			(1.97)										(1.41)				(1.40)
	Total	41	152	22	61	NA	NA	NA	1	NA	NA	63	213	NA	1	63	214
**Myocarditis & Death**	Median	7	49	NA	27	NA	NA	NA	NA	NA	NA	NA	27	NA	NA	7	27
	< 6 months	NA	NA	NA	NA	NA	NA	NA	NA	NA	NA	NA	NA	NA	NA	NA	NA
	6 months - 11 years	1 (100.00)	NA	NA	NA	NA	NA	NA	NA	NA	NA	NA	NA	NA	NA	1 (100.00)	NA
	12 - 17 years	NA	NA	NA	NA	NA	NA	NA	NA	NA	NA	NA	NA	NA	NA	NA	NA
	18 - 44 years	NA	2 (50.00)	NA	1 (100.00)	NA	NA	NA	NA	NA	NA	NA	3 (60.00)	NA	NA	NA	3 (60.00)
	45 - 64 years	NA	NA	NA	NA	NA	NA	NA	NA	NA	NA	NA	NA	NA	NA	NA	NA
	≥65 years	NA	2 (50.00)	NA	NA	NA	NA	NA	NA	NA	NA	NA	2 (40.00)	NA	NA	NA	2 (40.00)
	Unknown	NA	NA	NA	NA	NA	NA	NA	NA	NA	NA	NA	NA	NA	NA	NA	NA
	Total	1	4	NA	1	NA	NA	NA	NA	NA	NA	NA	5	NA	NA	1	5
**Pericarditis**	Median	56	36	70	34	NA	NA	NA	NA	NA	NA	59	36	NA	NA	59	36
	< 6 months	NA	NA	NA	NA	NA	NA	NA	NA	NA	NA	NA	NA	NA	NA	NA	NA
	6 months - 11 years	NA	NA	NA	NA	NA	NA	NA	NA	NA	NA	NA	NA	NA	NA	NA	NA
	12 - 17 years	NA	2 (7.6	NA	NA	NA	NA	NA	NA	NA	NA	NA	2 (5.5	NA	NA	NA	2 (5.56

			9)										6)				)
	18 - 44 years	3 (21.43)	14 (53.85)	NA	7 (70.00)	NA	NA	NA	NA	NA	NA	3 (18.75)	21 (58.33)	NA	NA	3 (18.75)	21 (58.33)
	45 - 64 years	6 (42.86)	3 (11.54)	NA	NA	NA	NA	NA	NA	NA	NA	6 (37.50)	3 (8.33)	NA	NA	6 (37.50)	3 (8.33)
	≥65 years	5 (35.71)	7 (26.92)	2 (100.00)	3 (30.00)	NA	NA	NA	NA	NA	NA	7 (43.75)	10 (27.78)	NA	NA	7 (43.75)	10 (27.78)
	Unknown	NA	NA	NA	NA	NA	NA	NA	NA	NA	NA	NA	NA	NA	NA	NA	NA
	Total	14	26	2	10	NA	NA	NA	NA	NA	NA	16	36	NA	NA	16	36
**Pericarditis & Death**	Median	NA	56	NA	30	NA	NA	NA	NA	NA	NA	NA	30	NA	NA	NA	30
	< 6 months	NA	NA	NA	NA	NA	NA	NA	NA	NA	NA	NA	NA	NA	NA	NA	NA
	6 months - 11 years	NA	NA	NA	NA	NA	NA	NA	NA	NA	NA	NA	NA	NA	NA	NA	NA
	12 - 17 years	NA	NA	NA	NA	NA	NA	NA	NA	NA	NA	NA	NA	NA	NA	NA	NA
	18 - 44 years	NA	1 (50.00)	NA	1 (100.00)	NA	NA	NA	NA	NA	NA	NA	2 (66.67)	NA	NA	NA	2 (66.67)
	45 - 64 years	NA	NA	NA	NA	NA	NA	NA	NA	NA	NA	NA	NA	NA	NA	NA	NA
	≥65 years	NA	1 (50.00)	NA	NA	NA	NA	NA	NA	NA	NA	NA	1 (33.33)	NA	NA	NA	1 (33.33)

	Unknown	NA	NA	NA	NA	NA	NA	NA	NA	NA	NA	NA	NA	NA	NA	NA	NA
	Total	NA	2	NA	1	NA	NA	NA	NA	NA	NA	NA	3	NA	NA	NA	3
**Myocarditis and Pericarditis**	Median	38	22	45	25	NA	NA	NA	81	NA	NA	43	23	NA	81	43	23
	< 6 months	NA	NA	NA	NA	NA	NA	NA	NA	NA	NA	NA	NA	NA	NA	NA	NA
	6 months - 11 years	3 (5.45)	2 (1.12)	1 (4.17)	3 (4.23)	NA	NA	NA	NA	NA	NA	4 (5.06)	5 (2.01)	NA	NA	4 (5.06)	5 (2.00)
	12 - 17 years	10 (18.18)	54 (30.34)	2 (8.33)	3 (4.23)	NA	NA	NA	NA	NA	NA	12 (15.19)	57 (22.89)	NA	NA	12 (15.19)	57 (22.80)
	18 - 44 years	18 (32.73)	92 (51.69)	8 (33.33)	54 (76.06)	NA	NA	NA	NA	NA	NA	26 (32.91)	146 (58.63)	NA	NA	26 (32.91)	146 (58.40)
	45 - 64 years	16 (29.09)	15 (8.43)	9 (37.50)	8 (11.27)	NA	NA	NA	NA	NA	NA	25 (31.65)	23 (9.24)	NA	NA	25 (31.65)	23 (9.20)
	≥65 years	8 (14.55)	12 (6.74)	4 (16.67)	3 (4.23)	NA	NA	NA	1 (100.00)	NA	NA	12 (15.19)	15 (6.02)	NA	1 (100.00)	12 (15.19)	16 (6.40)
	Unknown	NA	3 (1.69)	NA	NA	NA	NA	NA	NA	NA	NA	NA	3 (1.20)	NA	NA	NA	3 (1.20)
	Total	55	178	24	71	NA	NA	NA	1	NA	NA	79	249	NA	1	79	250
**Myocarditis and Pericarditis**	Median	7	49	NA	28	NA	NA	NA	NA	NA	NA	7	28	NA	NA	7	28
	< 6 months	NA	NA	NA	NA	NA	NA	NA	NA	NA	NA	NA	NA	NA	NA	NA	NA
	6 months -	1	NA	NA	NA	NA	NA	NA	NA	NA	NA	1	NA	NA	NA	1	NA

**& Death**	11 years	(100.00)										(100.00)				(100.00)	
	12 - 17 years	NA	NA	NA	NA	NA	NA	NA	NA	NA	NA	NA	NA	NA	NA	NA	NA
	18 - 44 years	NA	3 (50.00)	NA	2 (100.00)	NA	NA	NA	NA	NA	NA	NA	5 (62.50)	NA	NA	NA	5 (62.50)
	45 - 64 years	NA	NA	NA	NA	NA	NA	NA	NA	NA	NA	NA	NA	NA	NA	NA	NA
	≥65 years	NA	3 (50.00)	NA	NA	NA	NA	NA	NA	NA	NA	NA	3 (37.50)	NA	NA	NA	3 (37.50)
	Unknown	NA	NA	NA	NA	NA	NA	NA	NA	NA	NA	NA	NA	NA	NA	NA	NA
	Total	1	6	NA	2	NA	NA	NA	NA	NA	NA	1	8	NA	NA	1	8
