# Supplementary material for: Post-Marketing Safety of mRNA Vaccines: A Real-World Study Integrating Literature Case Reports and Vaccine Adverse Event Reporting System
Source: Vaccines (Basel). 2026 Jun 12;14(6):524. doi: 10.3390/vaccines14060524 (PMC13308135; doi:10.3390/vaccines14060524)
Supplement: Supplementary file 1 [file vaccines-14-00524-s001.zip › Table S19.pdf]

Table S19. Safety characteristics of mRNA vaccines in clinical trials.

Author's name	Year	DOI	NCT Number	Phase	Vaccine	Conditions	Dosage	With drug= N	AEFIs (%)	No.of Serious Adverse events (%)	Discount, due to AE (%)	No. of deaths due to AE (%)	Report AEFIs
Haranaka et al	2021	10.1038/s41467-021-27316-2	NCT04588480	Ⅳ	Comirnaty	NA	30 μg × 2	119	12（10.1）	0	1（0.8）	0	Fever（+++）、injection site Pain（+++）、Redness（++）、Swelling（++）、Fatigue（+++）、Headache（++）、Chills（+++）、Vomiting（+）、Diarrhea（++）、Muscle pain (++)、Joint pain (+++)
Simões et al	2023	10.1093/jpids/piad015	NCT04816643	Ⅱ/Ⅲ	Comirnaty	NA	10 μg × 3	401	36（9）	0	0	0	Fever（++++）、Pain at the injection site（++）、Redness（++）、Swelling（++）、Fatigue（+++）、Headache（++）、Chills（+++）、Vomiting（++）、Diarrhea（++）、Muscle pain (+++)、Joint pain (++)
Muñoz et al	2023	10.1056/NEJMoa2211031	NCT04816643	Ⅱ/Ⅲ	Comirnaty	6month-2years	3 μg × 3	1178	355（30.1）	17（1.4）	3（0.3）	0	Fever（++++）、Tenderness（++）、Redness（++）、Swelling（++）、Decreased appetite (loss of appetite)（+++）、Drowsiness (increased sleep)（+++）、Irritability (fussiness)（+++）
Muñoz et al	2023	10.1056/NEJMoa2211031	NCT04816643	Ⅱ/Ⅲ	Comirnaty	2-4years	3 μg × 3	1835	344（18.7）	12（0.7）	3（0.2）	0	Fever（++++）、Pain（++）、Redness（++）、Swelling（++）、Vomiting（++）、Diarrhea（++）、Headache（++）、Fatigue/tiredness（+++）、Chills（++）、Muscle pain（++）、

													Joint pain（++）
Polack et al	2020	10.1056/NEJMoa2034577	NCT04368728	II /III	Comirnaty	NA	30 μg ×2	21,720	5865（27）	4（0.02）	0	0	Fever（+++）、Pain at injection site（+++）、Redness（+++）、Swelling（+++）、Fatigue（+++）、Headache（+++）、Chills（+）、Vomiting（+）、Diarrhea（++）、Muscle pain (+++)、Joint pain (+++)
Frenck et al	2021	10.1056/NEJMoa2107456	NCT04368728	II /III	Comirnaty	12-15years	30 μg ×2	1131	68（6）	7 (0.6)	3	0	Fever（+++）、Pain at injection site（+++）、Redness（++）、Swelling（++）、Fatigue（+++）、Headache（+++）、Chills（++）、Vomiting（+）、Diarrhea（++）、Muscle pain (++)、Joint pain (++)
Frenck et al	2021	10.1056/NEJMoa2107456	NCT04368728	II /III	Comirnaty	16-25years	30 μg ×2	536	58 (10.8)	9 (1.7)	1	0	Fever（+++）、Pain at injection site（+++）、Redness（++）、Swelling（++）、Fatigue（+++）、Headache（+++）、Chills（++）、Vomiting（+）、Diarrhea（++）、Muscle pain (++)、Joint pain (++)
Thomas et al	2021	10.1056/NEJMoa2110345	NCT04368728	III	Comirnaty	NA	30 μg	21926	6617 (30.2)	127 (0.6)	32（0.1）	0	Fever（+++）、Pain at injection site（+++）、Redness（+++）、Swelling（++）、Fatigue（+++）、Headache（+++）、Chills（++）、Vomiting（+++）、Diarrhea（++）、Muscle pain (+++)、Joint pain (+++)
Thoma	2021	10.1016/j.va	NCT043	III	Comirnat	Cancer &	30 μg	1898	669	47（2.5）	6 (0.3)	1（0.05）	Injection site pain、Fatigue、Pyrexia、

s et al		ccine.2021.12.046	68728		y	COVID-19			（35）				Chills、Headache、Myalgia、Pain、Arthralgia、 Nausea、Injection site erythema、Pain in extremity、Diarrhea、Injection site swelling
Moreira et al	2022	10.1056/NEJMoa2200674	NCT04955626	III	Comirnaty	NA	30 μg	5055	2100 (42)	3 (0.06)	0	0	Blood or lymphatic system disorder、Lymphadenopathy、Cardiac disorder、Ear or labyrinth disorder、Endocrine disorder、Eye disorder、Gastrointestinal disorder、Nausea、Diarrhea、Vomiting、General disorder or injection-site condition、Injection site、Pain、Erythema、Swelling、Fatigue、Pyrexia、Chills、Pain、Malaise、Axillary pain、Asthenia、Hepatobiliary disorder、Immune system disorder、Infection or infestation、Injury, poisoning, or procedural complication、Investigations、Increased body temperature、Metabolism or nutrition disorder、Decreased appetite、Musculoskeletal or connective-tissue disorder、Myalgia、Arthralgia、Limb pain、Neck pain、Benign, malignant, or unspecified neoplasm、Nervous system disorder、Headache、Lethargy、

													Dizziness、Psychiatric disorder、Renal or urinary disorder、Reproductive system or breast disorder、Respiratory, thoracic, or mediastinal disorder、Skin or subcutaneous tissue disorder、Surgical or medical procedure、Vascular disorder
Hui al	2022	10.1016/j.lanwpc.2022.100586	NCT04649021	II	Comirnaty	18–85 years	30 μg	720	111（15.4）	5 (0.7)	4 (0.6)	0	Fever（+++）、Pain（++）、Swelling（++）、Redness（++）、Fatigue（++）、Headache（++）、Chills（++）、Nausea/vomiting（++）、Diarrhea（++）、Muscle pain (++)、Joint pain (++)
El Sahly et al	2021	10.1056/NEJMoa2113017	NCT04470427	III	Spikevax	NA	100 μg ×2	15184	4752 (31.3)	98（0.6）	61（0.4）	0	Fever（++++）、Pain（+++）、Erythema（+++）、Swelling（+++）、Axillary swelling/tenderness（+++）、Fatigue（+++）、Headache（+++）、Chills（+++）、Nausea/vomiting（+++）、Myalgia（+++）、Arthralgia (+++)
Chu et al	2021	10.1016/j.vaccine.2021.02.007	NCT04405076	II	Spikevax	NA	50/100 μg ×2	400	113（28.25）	12 (3)	0	0	Fever（++）、Pain（+++）、Erythema（+）、Swelling（++）、Axillary swelling/tenderness（++）、Fatigue（+++）、Headache（+++）、Chills（+++）、Nausea/vomiting（+++）、Myalgia（+++）、Arthralgia (+++)、Rash
Baden	2020	10.1056/NE	NCT044	III	Spikevax	NA	100 μg	15185	3632	93 (0.6)	50 (0.3)	2（0.01）	Nervous system disorders、

et al		JMoa20353 89	70427				×2		(23.9)				Headache 、Respiratory, thoracic and mediastinal disorders、Cough 、 Oropharyngeal pain、Gastrointestinal disorders、Diarrhea、Musculoskeletal and connective tissue disorders、 Arthralgia 、Myalgia 、General disorders and administration site conditions 、Fatigue 、Injection site pain
Ali et al	2021	10.1056/NE JMoa21095 22	NCT046 49151	II /III	Spikevax	NA	100 μg ×2	2486	510 (20.5)	0	1 （<0.1 ）	0	Fever（++++）、Pain（+++）、Erythema （+++）、Swelling（+++）、Axillary swelling/tenderness（+++）、Fatigue （+++）、Headache（++++）、Chills （+++）、 Nausea/vomiting（++++）、 Myalgia（+++） 、 Arthralgia (+++)
Creech et al	2022	10.1056/NE JMoa22033 15	NCT047 96896	II /III	Spikevax	Open-label dose-selecti on phase	50 μg / 100 μg	751	219 (29.2)	1 (0.1)	1 (0.1)	0	Fever（++++）、Pain（+++）、Erythema （+++）、Swelling（+++）、Axillary swelling/tenderness（+++）、Fatigue （+++）、Headache（+++）、Chills（+++）、 Nausea/vomiting（+++） 、Myalgia （+++） 、 Arthralgia (+++)
Creech et al	2022	10.1056/NE JMoa22033 15	NCT047 96896	II /III	Spikevax	Controlled expansion phase	50 μg ×2	3007	891 (29.6)	3 (<0.1)	2 (<0.1)	0	Fever（+++）、Pain（+++）、Erythema （+++）、Swelling（+++）、Axillary swelling/tenderness（+++）、Fatigue （+++）、Headache（+++）、Chills（+++）、 Nausea/vomiting（+++） 、Myalgia

													（+++） 、 Arthralgia (+++)
Berthaud et al	2024	10.1093/cid/ciae420	NCT04796896	II/III	Spikevax	6month–5years	10 μg	153	97 (63.4)	1 (0.7)	0	0	COVID-19、Injection site cellulitis、Serum sickness-like reaction、Decreased appetite、Headache、Supraventricular tachycardia、Constipation、Urticaria、Alopecia、Idiopathic urticaria、Arthralgia、Myalgia、Fatigue、Pyrexia、Chills、Injection site hematoma、Injection site lymphadenopathy、Injection site pain、Injection site urticaria、Non-cardiac chest pain、Vaccination site lymphadenopathy、Weight decreased、Headache、Vomiting、Arthralgia、Myalgia、Fatigue、Injection site pain、Pyrexia、Chills
Berthaud et al	2024	10.1093/cid/ciae420	NCT04796896	II/III	Spikevax	6–11years	25 μg	2519	1152 (45.7)	11 (0.4)	0	0	COVID-19、Injection site cellulitis、Serum sickness-like reaction、Decreased appetite、Headache、Supraventricular tachycardia、Constipation、Urticaria、Alopecia、Idiopathic urticaria、Arthralgia、Myalgia、Fatigue、Pyrexia、Chills、Injection site hematoma、Injection site lymphadenopathy、Injection site pain、Injection site urticaria、Non-cardiac

													chest pain、Vaccination site lymphadenopathy、Weight decreased、Headache、Vomiting、Arthralgia、Myalgia、Fatigue、Injection site pain、Pyrexia、Chills
Anderson et al	2022	10.1056/NEJMoa2209367	NCT04796896	Ⅱ/Ⅲ	Spikevax	6-23years	25 μg × 2	1911	869 (49.3)	8 (0.5)	1 (<0.1)	0	Fever（+++）、Pain（++）、Erythema（+++）、Swelling（+++）、Axillary swelling/tenderness（++）、Irritability/Crying（++）、Sleepiness（++）、Loss of Appetite（+++）
Anderson et al	2022	10.1056/NEJMoa2209367	NCT04796896	Ⅱ/Ⅲ	Spikevax	2-5years	25 μg × 2	3031	1212 (40.0)	4 (0.1)	0	0	Fever（+++）、Pain（++）、Erythema（+++）、Swelling（+++）、Axillary swelling/tenderness（++）、Irritability/Crying（++）、Sleepiness（++）、Loss of Appetite（+++）
Figueroa et al	2024	10.1093/infdis/jiae140	NCT04860297	Ⅲ	Spikevax	Covid-19& Solid Organ Transplant	100 μg × 3	214	90 (42.1)	13 (6.1)	0	0	Fever（+++）、Pain（+++）、Erythema（+++）、Swelling（+++）、Axillary swelling/tenderness（+++）、Fatigue（+++）、Headache（+++）、Chills（+++）、Nausea/vomiting（++）、Myalgia（+++）、Arthralgia (+++)、Rash
Shaw et al	2024	10.1093/infdis/jiae081	NCT04528719	Ⅰ	mRESVIA	NA	12.5, 25, 50, 100, 200	239	135 (56.5)	14 (5.9)	1 (0.4)	0	Fever（+）、injection site Pain（+++）、Erythema（+）、Swelling（+）、Fatigue（+++）、Headache（++）、Chills（+++）、Vomiting（+++）、Arthralgia（++）、Myalgia (+++)、Lymphadenopathy (++)

Shaw et al	2024	10.1093/infdis/jiae035	NCT04528719	Ⅰ	mRESVIA	NA	50, 100, 200; 100×3	71	9（12.7）	0	0	0	Fever（++）、Pain（+++）、Erythema（++）、Swelling（++）、Fatigue（+++）、Headache（+++）、Chills（++）、Nausea/vomiting（++）、Myalgia（+++）、Arthralgia (+++)、Lymphadenopathy（++）
Usdan et al	2023	10.1093/cid/ciad718	NCT05472038	Ⅱ/Ⅲ	Comirnaty Bivalent	Comirnaty-Omi.BA.4/BA.5	30 μg，60 μg	938	64（6.8）	3（0.3）	0	0	Fever（+++）、Pain at the injection site（++）、Redness（+++）、Swelling（++）、Vomiting（++）、Diarrhea（++）、Headache（+++）、Fatigue/tiredness（+++）、Chills（++）、Muscle pain（+++）、Joint pain（+++）
Chalkias et al	2023	10.1038/s41591-023-02517-y	NCT04927065	Ⅱ/Ⅲ	Spikevax Bivalent	NA	50 μg	511	129 (25.2)	3（0.6）	0	1（0.2）	Fever（+++）、Pain（+++）、Erythema（+++）、Swelling（+++）、Axillary swelling/tenderness（+++）、Fatigue（+++）、Headache（+++）、Chills（+++）、Nausea/vomiting（+++）、Myalgia（+++）、Arthralgia (+++)
