# Supplementary material for: Post-Marketing Safety of mRNA Vaccines: A Real-World Study Integrating Literature Case Reports and Vaccine Adverse Event Reporting System
Source: Vaccines (Basel). 2026 Jun 12;14(6):524. doi: 10.3390/vaccines14060524 (PMC13308135; doi:10.3390/vaccines14060524)
Supplement: Supplementary file 1 [file vaccines-14-00524-s001.zip › Table S2.pdf]

**Table S2.** PICOS of literature case reports

| PICOS Element | Definition |
|---|---|
| P (Population) | It was individuals of any age, sex, ethnicity, or health status who received any of the five target mRNA vaccines (Comirnaty, Spikevax, mRESVIA, Comirnaty Bivalent, Spikevax Bivalent) |
| I (Intervention/Exposure) | It was administration of at least one dose of a target mRNA vaccine |
| C (Comparator) | It eviewed a single-arm aggregate analysis and no comparator group was set |
| O (Outcomes) | It reported AEFIs following vaccination, including their time of onset, duration, and clinical outcomes |
| S (Study) | It included published case reports and case series involving any of the target mRNA vaccines. We excluded duplicate publications and studies unrelated to RNA vaccines, including those that were not case reports, involved non-target vaccines, or contained incomplete data (a total of 3,563 articles). |
