# Supplementary material for: Post-Marketing Safety of mRNA Vaccines: A Real-World Study Integrating Literature Case Reports and Vaccine Adverse Event Reporting System
Source: Vaccines (Basel). 2026 Jun 12;14(6):524. doi: 10.3390/vaccines14060524 (PMC13308135; doi:10.3390/vaccines14060524)
Supplement: Supplementary file 1 [file vaccines-14-00524-s001.zip › Table S3.pdf]

Table S3. AEFI terms without clear clinical significance.

SOC	PT
Product issues	No adverse event
Injury, poisoning and procedural complications	
Investigations	
Social circumstances	
Surgical and medical procedures	
