# Supplementary material for: Post-Marketing Safety of mRNA Vaccines: A Real-World Study Integrating Literature Case Reports and Vaccine Adverse Event Reporting System
Source: Vaccines (Basel). 2026 Jun 12;14(6):524. doi: 10.3390/vaccines14060524 (PMC13308135; doi:10.3390/vaccines14060524)
Supplement: Supplementary file 1 [file vaccines-14-00524-s001.zip › Table S4.pdf]

Table S4. Two-by-two contingency table for DPA.

**Item**	**Target AEFI Cases**	**Other AEFI Cases**	**Total**
Target Vaccine	a	b	$a+b$
Other Vaccines	c	d	$c+d$
Total	$a+c$	$b+d$	$N=a+b+c+d$

a: Number of cases with target AEFIs in the target vaccine; b: Number of cases with non-target AEFIs in the target vaccine; c: Number of cases with target AEFIs in the non-target vaccine; d: Number of cases with non-target AEFIs in the non-target vaccine.
