# Supplementary material for: Post-Marketing Safety of mRNA Vaccines: A Real-World Study Integrating Literature Case Reports and Vaccine Adverse Event Reporting System
Source: Vaccines (Basel). 2026 Jun 12;14(6):524. doi: 10.3390/vaccines14060524 (PMC13308135; doi:10.3390/vaccines14060524)
Supplement: Supplementary file 1 [file vaccines-14-00524-s001.zip › Table S5.pdf]

**Table S5.** The principles of disproportionate measurement and the criteria for signal detection of DPA.

| Method | Calculation formula | Criteria |
| --- | --- | --- |
| ROR | $$ROR = \frac{(a/c)}{(b/d)} = \frac{ad}{bc}$$ $$SE\ (ln\ ROR) = \sqrt{(\frac{1}{a} + \frac{1}{b} + \frac{1}{c} + \frac{1}{d})}$$ $$95\%CI = e^{ln(ROR) \pm 1.96 SE(ln\ ROR)}$$ | a ≥ 3<br>$ROR_{025}$ > 1. |
| BCPNN | $$IC = log_2 \frac{p(x,y)}{p(x)p(y)} = log_2 \frac{a(a+b+c+d)}{(a+b)(a+c)}$$ $$E(IC) = \log_2 \frac{(a+\gamma_{11})(a+b+c+d+\alpha)(a+b+c+d+\beta)}{(a+b+c+d+\gamma)(a+b+\alpha_1)(a+c+\beta_1)}$$ $$V(IC) = \frac{1}{(\ln 2)^2}\left\{\left[\frac{(a+b+c+d)-a+\gamma-\gamma_{11}}{(a+\gamma_{11})(1+a+b+c+d+\gamma)}\right] + \left[\frac{(a+b+c+d)-(a+b)+\alpha-\alpha_1}{(a+b+\alpha_1)(1+a+b+c+d+\alpha)}\right] + \left[\frac{(a+b+c+d)-(a+c)+\beta-\beta_1}{(a+c+\beta_1)(1+a+b+c+d+\beta}\right]\right\}$$ $$\gamma = \gamma_{11} \frac{(a+b+c+d+\alpha)(a+b+c+d+\beta)}{(a+b+\alpha_1)(a+c+\beta 1)}$$ $$95\%CI = E(IC) \pm 2\sqrt{V(IC)}$$ $$\alpha_1 = \beta_1 = 1; \alpha = \beta = 2; \gamma_{11} = 1$$ | $IC_{025}$ > 0. |

a: Number of cases with target AEFIs in the target vaccine; b: Number of cases with non-target AEFIs in the target vaccine; c: Number of cases with target AEFIs in the non-target vaccine; d: Number of cases with non-target AEFIs in the non-target vaccine. N, the number of reports; ROR: reporting odds ratio; 95%CI, 95% confidence/credible interval; $ROR_{025}$: the lower limit of the 95% CI of the ROR; BCPNN: Bayesian confidence propagation neural network; IC: information component; $IC_{025}$: the lower limit of 95% CI of the IC; E(IC), the IC expectations; V(IC), the variance of IC.
