# Supplementary material for: Post-Marketing Safety of mRNA Vaccines: A Real-World Study Integrating Literature Case Reports and Vaccine Adverse Event Reporting System
Source: Vaccines (Basel). 2026 Jun 12;14(6):524. doi: 10.3390/vaccines14060524 (PMC13308135; doi:10.3390/vaccines14060524)
Supplement: Supplementary file 1 [file vaccines-14-00524-s001.zip › Table S6.pdf]

Table S6. Search terms of literature case reports.

Literature databases	Inclusion criteria	Exclusion criteria
PubMed	((Pfizer BioNTech/ Moderna COVID-19/ RSV Vaccine) OR (BNT162b2/ mRNA-1273/ mRNA-1345/ BNT162b5/ mRNA-1273.222) OR (Comirnaty/ Spikevax/ mRESVIA/ Comirnaty Bivalent/ Spikevax Bivalent)) AND (case report)	① Duplicate records: repeated entries are excluded. ② Reports not retrieved: any report that could not be accessed or obtained is excluded. ③ The research content is not coincident are excluded. ④ The reference type is not matched are excluded, including review, meta-analyses, conference papers, clinical trial results, animal experiments, letters.
Embase	(Pfizer BioNTech/ Moderna COVID-19/RSV Vaccine)/ br OR ((BNT162b2/ mRNA-1273/ mRNA-1345/ BNT162b5/ mRNA-1273.222)/ br) OR ((Comirnaty/ Spikevax/ mRESVIA/ Comirnaty Bivalent/ Spikevax Bivalent)/ br) AND ((case report)/ br)	
Web of science	TS= (((Pfizer BioNTech/ Moderna COVID-19/ RSV Vaccine) OR (BNT162b2/ mRNA-1273/ mRNA-1345/ BNT162b5/ mRNA-1273.222) OR (Comirnaty/ Spikevax/ mRESVIA/ Comirnaty Bivalent/ Spikevax Bivalent)) AND (case report))	
Scopus	TITLE-ABS-KEY (((Pfizer BioNTech/ Moderna COVID-19/ RSV Vaccine) OR (BNT162b2/ mRNA-1273/ mRNA-1345/ BNT162b5/ mRNA-1273.222) OR (Comirnaty/ Spikevax/ mRESVIA/ Comirnaty Bivalent/ Spikevax Bivalent)) AND (case report))	
