# Supplementary material for: Post-Marketing Safety of mRNA Vaccines: A Real-World Study Integrating Literature Case Reports and Vaccine Adverse Event Reporting System
Source: Vaccines (Basel). 2026 Jun 12;14(6):524. doi: 10.3390/vaccines14060524 (PMC13308135; doi:10.3390/vaccines14060524)
Supplement: Supplementary file 1 [file vaccines-14-00524-s001.zip › Table S7.pdf]

Table S7. Top-ranked positive PT of mRNA vaccines in VAERS.

Vaccines	PT	n	ROR	ROR025	ROR975	IC	IC025	IC975	SOC
**Comirnaty**	Breakthrough COVID-19	**176395**	2.32	2.30	2.34	0.44	0.43	0.45	Infections and infestations
	Dizziness	**76529**	1.14	1.13	1.15	0.09	0.07	0.10	Nervous system disorders
	Dyspnoea	**60640**	1.35	1.33	1.37	0.18	0.17	0.20	Respiratory, thoracic and mediastinal disorders
	Malaise	**40972**	1.25	1.23	1.27	0.14	0.12	0.16	General disorders and administration site conditions
	Asthenia	**38851**	1.11	1.10	1.13	0.07	0.05	0.09	General disorders and administration site conditions
	Chest pain	**37883**	1.67	1.64	1.70	0.30	0.28	0.32	General disorders and administration site conditions
	Paraesthesia	**30332**	1.36	1.33	1.38	0.19	0.17	0.21	Nervous system disorders
	Lymphadenopathy	**28545**	1.53	1.50	1.56	0.26	0.24	0.28	Blood and lymphatic system disorders
	Palpitations	**27104**	1.74	1.70	1.77	0.32	0.30	0.34	Cardiac disorders
	Hypoaesthesia	**26842**	1.31	1.28	1.33	0.17	0.15	0.19	Nervous system disorders
	Arrhythmia	13882	3.32	3.20	3.44	0.58	**0.54**	0.61	Cardiac disorders
	Suspected COVID-19	8106	4.45	4.22	4.69	0.66	**0.61**	0.70	Infections and infestations
	Dysmenorrhoea *	7237	3.46	3.28	3.64	0.59	**0.54**	0.63	Reproductive system and breast disorders
	Anaphylactic reaction #	7108	2.12	2.03	2.22	0.41	0.37	0.46	Immune system disorders
	Menstruation delayed *	4029	3.53	3.29	3.78	0.60	**0.53**	0.66	Reproductive system and breast disorders
	Disease recurrence *	3855	17.97	**15.55**	20.77	0.85	**0.79**	0.92	General disorders and administration site conditions

Postmenopausal haemorrhage *	3761	3.43	3.20	3.68	0.59	**0.52**	0.65	Reproductive system and breast disorders
Angioedema #	2921	1.40	1.32	1.49	0.21	0.14	0.28	Skin and subcutaneous tissue disorders
Deafness * #	2671	1.63	1.53	1.73	0.29	0.22	0.36	Ear and labyrinth disorders
Hypertensive crisis *	1784	3.95	3.55	4.40	0.63	**0.53**	0.72	Vascular disorders
Blindness * #	1717	1.30	1.20	1.40	0.16	0.08	0.25	Eye disorders
Sudden hearing loss * #	1351	1.98	1.80	2.18	0.38	0.28	0.49	Ear and labyrinth disorders
Anaphylactic shock * #	1251	1.92	1.74	2.12	0.37	0.26	0.48	Immune system disorders
Renal failure * #	1077	1.74	1.57	1.93	0.32	0.21	0.44	Renal and urinary disorders
Performance status decreased	840	4.55	3.85	5.38	0.66	**0.53**	0.80	General disorders and administration site conditions
Rhabdomyolysis * #	519	1.29	1.13	1.47	0.16	0.00	0.32	Musculoskeletal and connective tissue disorders
Pancreatitis acute * #	473	2.04	1.74	2.41	0.40	0.23	0.57	Gastrointestinal disorders
Pancreatitis * #	450	1.49	1.28	1.73	0.24	0.07	0.42	Gastrointestinal disorders
Ventricular fibrillation #	406	1.75	1.48	2.06	0.32	0.14	0.51	Cardiac disorders
Autoimmune hepatitis # *	366	1.81	1.51	2.16	0.34	0.15	0.54	Hepatobiliary disorders
Dysphoria *	310	7.49	**5.33**	10.53	0.76	**0.53**	0.98	Psychiatric disorders
Multisystem inflammatory syndrome in children *	232	7.15	**4.86**	10.52	0.75	0.49	1.01	Immune system disorders
Optic ischaemic neuropathy * #	218	1.91	1.51	2.42	0.37	0.11	0.62	Eye disorders
Autoimmune haemolytic anaemia * #	193	1.58	1.25	2.00	0.28	0.01	0.54	Blood and lymphatic system disorders
Hyperleukocytosis *	191	10.67	**6.41**	17.77	0.81	**0.52**	1.09	Blood and lymphatic system

									disorders
	Thrombotic thrombocytopenic purpura * #	162	1.77	1.35	2.30	0.33	0.04	0.62	Blood and lymphatic system disorders
	Sleep disorder due to a general medical condition *	151	9.00	**5.29**	15.30	0.78	0.46	1.11	Psychiatric disorders
	Haemolysis * #	151	1.80	1.36	2.37	0.34	0.04	0.64	Blood and lymphatic system disorders
	Ocular vascular disorder *	135	9.28	**5.25**	16.40	0.79	0.45	1.13	Eye disorders
	Sudden cardiac death * #	125	1.77	1.31	2.40	0.33	0.00	0.66	General disorders and administration site conditions
	Neoplasm progression *	119	10.64	**5.58**	20.28	0.81	0.44	1.17	Neoplasms benign, malignant and unspecified (incl cysts and polyps)
	Dermatitis exfoliative generalised * #	114	1.92	1.39	2.66	0.37	0.02	0.72	Skin and subcutaneous tissue disorders
	Sleep disorder due to general medical condition, insomnia type *	104	13.28	**6.18**	28.55	0.83	0.44	1.22	Psychiatric disorders
	Toxoplasmosis *	99	9.83	**4.97**	19.46	0.80	0.40	1.20	Infections and infestations
	Marasmus *	39	34.87	**4.79**	253.78	0.88	0.24	1.53	Metabolism and nutrition disorders
**Spikevax**	Headache	**85601**	1.19	1.18	1.20	0.16	0.15	0.17	Nervous system disorders
	Pyrexia	**80507**	1.35	1.34	1.36	0.28	0.26	0.29	General disorders and administration site conditions
	Fatigue	**75257**	1.19	1.18	1.20	0.17	0.15	0.18	General disorders and administration site conditions

Chills	**61625**	1.63	1.61	1.64	0.44	0.43	0.45	General disorders and administration site conditions
Pain	**52765**	1.21	1.19	1.22	0.18	0.16	0.19	General disorders and administration site conditions
Nausea	**46555**	1.19	1.17	1.20	0.16	0.15	0.18	Gastrointestinal disorders
Pain in extremity *	**46246**	1.25	1.24	1.27	0.21	0.20	0.23	Musculoskeletal and connective tissue disorders
Vaccination site pain	**43080**	1.60	1.58	1.62	0.43	0.41	0.44	General disorders and administration site conditions
Myalgia	**40975**	1.44	1.42	1.46	0.34	0.32	0.35	Musculoskeletal and connective tissue disorders
Vaccination site erythema	**32297**	3.14	**3.09**	3.19	0.92	**0.90**	0.94	General disorders and administration site conditions
Vaccination site pruritus	21797	5.64	**5.50**	5.78	1.22	**1.20**	1.25	General disorders and administration site conditions
Vaccination site warmth	16399	3.96	**3.86**	4.06	1.06	**1.03**	1.08	General disorders and administration site conditions
Vaccination site rash	12673	5.78	**5.59**	5.97	1.23	**1.20**	1.27	General disorders and administration site conditions
Vaccination site induration	6219	4.31	**4.13**	4.49	1.10	**1.05**	1.15	General disorders and administration site conditions
Vaccination site urticaria	1586	3.27	**3.03**	3.53	0.95	**0.86**	1.04	General disorders and administration site conditions
Mechanical urticaria	960	8.45	**7.37**	9.69	1.37	**1.25**	1.50	Skin and subcutaneous tissue disorders
Erythema multiforme #	523	1.20	1.08	1.33	0.17	0.03	0.32	Skin and subcutaneous tissue

									disorders
	Sedation *	402	3.77	**3.21**	4.42	1.03	**0.85**	1.22	Nervous system disorders
	Type IV hypersensitivity reaction	177	5.87	**4.44**	7.75	1.24	**0.96**	1.53	Immune system disorders
	Vaccination site anaesthesia	33	12.58	**5.27**	30.02	1.48	**0.80**	2.15	General disorders and administration site conditions
**mRESVIA**	Arthralgia	**5**	2.53	**1.03**	6.19	1.30	**0.10**	2.51	Musculoskeletal and connective tissue disorders
	Feeling abnormal *	**3**	3.77	**1.20**	11.84	1.89	**0.42**	3.35	General disorders and administration site conditions
	Muscular weakness	**3**	7.36	**2.34**	23.16	2.85	**1.38**	4.32	Musculoskeletal and connective tissue disorders
	Rash	**4**	2.90	**1.07**	7.87	1.51	**0.19**	2.82	Skin and subcutaneous tissue disorders
**MNEXSPIKE**	Pain in extremity *	**56**	1.38	1.06	1.81	0.46	0.07	0.84	Musculoskeletal and connective tissue disorders
	Vaccination site pain	**55**	1.70	1.30	2.22	0.75	0.36	1.14	General disorders and administration site conditions
	Vomiting	**31**	1.54	1.08	2.20	0.61	0.10	1.13	Gastrointestinal disorders
	Loss of consciousness *	**25**	3.04	**2.05**	4.51	1.59	1.02	2.16	Nervous system disorders
	Urticaria *	**24**	1.96	1.31	2.93	0.96	0.38	1.54	Skin and subcutaneous tissue disorders
	Vaccination site swelling	**24**	1.59	1.06	2.37	0.66	0.08	1.24	General disorders and administration site conditions
	Erythema	**22**	1.68	1.11	2.57	0.75	0.14	1.35	Skin and subcutaneous tissue disorders

	Hyperhidrosis *	**19**	1.60	1.02	2.52	0.67	0.03	1.32	Skin and subcutaneous tissue disorders
	Gait disturbance *	**14**	2.40	1.42	4.06	1.26	0.51	2.01	General disorders and administration site conditions
	Feeling hot	**14**	2.23	1.32	3.77	1.15	0.40	1.90	General disorders and administration site conditions
	Injected limb mobility decreased *	10	6.49	**3.48**	12.09	2.69	**1.82**	3.56	General disorders and administration site conditions
	Vaccination site bruising	9	5.26	**2.73**	10.13	2.39	**1.47**	3.30	General disorders and administration site conditions
	Dysstasia *	8	4.68	**2.34**	9.38	2.22	**1.26**	3.18	Nervous system disorders
	Brain fog *	4	7.00	**2.62**	18.68	2.80	**1.51**	4.10	Nervous system disorders
	Temperature intolerance *	3	13.61	**4.38**	42.35	3.76	**2.31**	5.21	General disorders and administration site conditions
	Conjunctival haemorrhage *	3	12.68	**4.08**	39.45	3.66	**2.21**	5.11	Eye disorders
	Ataxia *	3	10.49	**3.37**	32.62	3.38	**1.94**	4.83	Nervous system disorders
	Shoulder injury related to vaccine administration *	3	9.43	**3.03**	29.30	3.23	**1.78**	4.68	General disorders and administration site conditions
	Bursitis *	3	7.25	**2.33**	22.51	2.85	**1.41**	4.30	Musculoskeletal and connective tissue disorders
	Vaccination site haemorrhage	3	5.80	1.87	18.01	2.53	**1.08**	3.98	General disorders and administration site conditions
**Comirnaty Bivalent**	Breakthrough COVID-19	**3746**	1.44	1.39	1.49	0.49	0.44	0.54	Infections and infestations
	Cough	**2028**	3.72	3.56	3.89	1.82	1.75	1.89	Respiratory, thoracic and mediastinal disorders
	Oropharyngeal pain	**1122**	3.96	3.73	4.20	1.92	1.83	2.01	Respiratory, thoracic and

								mediastinal disorders
Malaise	**1084**	1.44	1.36	1.53	0.51	0.42	0.60	General disorders and administration site conditions
Respiratory tract congestion	**887**	10.01	**9.34**	10.73	3.17	**3.07**	3.28	Respiratory, thoracic and mediastinal disorders
Asthenia	**825**	1.09	1.02	1.17	0.12	0.02	0.23	General disorders and administration site conditions
Rhinorrhoea	**736**	4.66	4.32	5.02	2.15	2.04	2.26	Respiratory, thoracic and mediastinal disorders
Condition aggravated	**644**	1.94	1.79	2.10	0.93	0.82	1.05	General disorders and administration site conditions
Feeling abnormal	**572**	1.35	1.25	1.47	0.43	0.31	0.55	General disorders and administration site conditions
Acute respiratory failure	**467**	6.84	6.22	7.51	2.68	2.54	2.82	Respiratory, thoracic and mediastinal disorders
Symptom recurrence	228	22.23	**19.23**	25.70	4.17	**3.96**	4.38	General disorders and administration site conditions
Acute kidney injury #	116	2.41	2.00	2.89	1.24	0.97	1.51	Renal and urinary disorders
Obstructive sleep apnoea syndrome	110	16.02	**13.08**	19.63	3.78	**3.49**	4.08	Respiratory, thoracic and mediastinal disorders
Chronic respiratory failure	76	14.52	**11.40**	18.50	3.66	**3.31**	4.01	Respiratory, thoracic and mediastinal disorders
Paranasal sinus hypersecretion	55	20.84	**15.55**	27.93	4.10	**3.68**	4.52	Respiratory, thoracic and mediastinal disorders
Pulmonary hypertension #	18	3.44	2.15	5.51	1.75	1.07	2.42	Respiratory, thoracic and mediastinal disorders

	Rhabdomyolysis #	16	1.70	1.04	2.79	0.75	0.05	1.46	Musculoskeletal and connective tissue disorders
	Thalamic stroke	7	22.91	**10.01**	52.45	4.21	**3.08**	5.34	Nervous system disorders
	Febrile neutropenia #	5	3.72	1.52	9.11	1.86	0.65	3.06	Blood and lymphatic system disorders
	Idiopathic partial epilepsy	3	54.98	**13.14**	230.07	5.12	**3.38**	6.85	Nervous system disorders
	Oropharyngeal discolouration	3	39.27	**10.15**	151.87	4.80	**3.11**	6.48	Respiratory, thoracic and mediastinal disorders
	Pulmonary septal thickening	3	30.54	**8.27**	112.83	4.53	**2.88**	6.18	Respiratory, thoracic and mediastinal disorders
	Scalp haematoma	3	30.54	**8.27**	112.83	4.53	**2.88**	6.18	Vascular disorders
**Spikevax Bivalent**	Breakthrough COVID-19	**2895**	1.46	1.41	1.52	0.51	0.46	0.57	Infections and infestations
	Fatigue	**2027**	1.12	1.07	1.18	0.16	0.09	0.23	General disorders and administration site conditions
	Pyrexia	**1953**	1.09	1.04	1.14	0.12	0.05	0.19	General disorders and administration site conditions
	Cough	**1563**	3.74	3.56	3.94	1.84	1.76	1.91	Respiratory, thoracic and mediastinal disorders
	Pain	**1514**	1.21	1.15	1.27	0.26	0.19	0.34	General disorders and administration site conditions
	Oropharyngeal pain	**897**	4.14	3.87	4.42	1.99	1.89	2.09	Respiratory, thoracic and mediastinal disorders
	Malaise	**856**	1.49	1.40	1.60	0.57	0.47	0.67	General disorders and administration site conditions
	Respiratory tract congestion	**685**	9.94	**9.19**	10.75	3.19	**3.08**	3.31	Respiratory, thoracic and mediastinal disorders

	Rhinorrhoea	**594**	4.91	**4.52**	5.33	2.24	**2.11**	2.36	Respiratory, thoracic and mediastinal disorders
	Feeling abnormal	**440**	1.37	1.24	1.50	0.44	0.30	0.58	General disorders and administration site conditions
	Sneezing	178	5.46	**4.70**	6.35	2.39	**2.17**	2.61	Respiratory, thoracic and mediastinal disorders
	Upper-airway cough syndrome	86	9.36	**7.52**	11.66	3.13	**2.81**	3.45	Respiratory, thoracic and mediastinal disorders
	Secretion discharge	63	6.86	**5.32**	8.84	2.71	**2.34**	3.08	General disorders and administration site conditions
	Paranasal sinus hypersecretion	37	17.18	**12.17**	24.25	3.92	**3.42**	4.42	Respiratory, thoracic and mediastinal disorders
	Pulmonary fibrosis #	11	2.95	1.62	5.36	1.54	0.69	2.38	Respiratory, thoracic and mediastinal disorders
	Nasal disorder	9	9.70	**4.92**	19.12	3.18	**2.23**	4.13	Respiratory, thoracic and mediastinal disorders
	Lower urinary tract symptoms	6	10.97	**4.76**	25.30	3.34	**2.20**	4.48	Renal and urinary disorders
	Nasal mucosal discolouration	4	53.63	**16.51**	174.14	5.23	**3.72**	6.73	Respiratory, thoracic and mediastinal disorders
	Pulmonary septal thickening	3	40.22	**10.89**	148.56	4.93	**3.28**	6.58	Respiratory, thoracic and mediastinal disorders
**Monovalent mRNA vaccines**	Breakthrough COVID-19	**220482**	1.73	1.71	1.75	0.10	0.09	0.11	Infections and infestations
	Headache	**214645**	1.18	1.16	1.19	0.04	0.03	0.04	Nervous system disorders
	Fatigue	**191123**	1.32	1.30	1.33	0.06	0.05	0.07	General disorders and administration site conditions
	Nausea	**116539**	1.17	1.15	1.18	0.03	0.02	0.05	Gastrointestinal disorders

Dizziness	**115589**	1.11	1.09	1.12	0.02	0.01	0.03	Nervous system disorders
Myalgia	**93581**	1.46	1.43	1.48	0.08	0.06	0.09	Musculoskeletal and connective tissue disorders
Dyspnoea	**88211**	1.39	1.36	1.41	0.07	0.06	0.08	Respiratory, thoracic and mediastinal disorders
Arthralgia	**84207**	1.21	1.18	1.23	0.04	0.03	0.05	Musculoskeletal and connective tissue disorders
Asthenia	**59382**	1.12	1.09	1.14	0.03	0.01	0.04	General disorders and administration site conditions
Chest pain	**52492**	1.83	1.78	1.88	0.11	0.10	0.13	General disorders and administration site conditions
Lymphadenopathy	41935	2.57	2.48	2.66	0.16	**0.14**	0.17	Blood and lymphatic system disorders
Myocarditis	17579	5.20	**4.82**	5.61	0.21	**0.18**	0.24	Cardiac disorders
Arrhythmia	16709	3.64	3.40	3.89	0.19	**0.16**	0.22	Cardiac disorders
Heavy menstrual bleeding	15790	3.75	**3.50**	4.02	0.19	**0.16**	0.22	Reproductive system and breast disorders
Pericarditis	11636	3.98	**3.66**	4.32	0.19	**0.16**	0.23	Cardiac disorders
Axillary pain	10905	2.84	2.64	3.06	0.17	**0.13**	0.20	General disorders and administration site conditions
Anaphylactic reaction #	8920	1.51	1.43	1.61	0.08	0.04	0.13	Immune system disorders
Dysmenorrhoea	8714	4.41	**3.99**	4.87	0.20	**0.16**	0.24	Reproductive system and breast disorders
Menstruation delayed	4868	5.35	**4.62**	6.21	0.21	**0.15**	0.27	Reproductive system and breast disorders
Intermenstrual bleeding	4224	3.95	3.45	4.53	0.19	**0.13**	0.25	Reproductive system and breast

									disorders
	Disease recurrence	3976	11.27	**8.91**	14.25	0.24	**0.17**	0.30	General disorders and administration site conditions
	Sudden hearing loss #	1749	1.66	1.44	1.91	0.10	0.00	0.19	Ear and labyrinth disorders
	Performance status decreased	970	5.57	**3.98**	7.81	0.21	0.08	0.34	General disorders and administration site conditions
	Premenstrual syndrome	877	6.53	**4.45**	9.58	0.22	0.08	0.36	Reproductive system and breast disorders
	Menometrorrhagia	832	15.21	**8.39**	27.58	0.25	0.10	0.39	Reproductive system and breast disorders
	Hemianaesthesia	352	7.87	**4.06**	15.24	0.23	0.01	0.44	Nervous system disorders
**Bivalent mRNA vaccines**	Breakthrough COVID-19	**6641**	1.45	1.42	1.49	0.50	0.47	0.54	Infections and infestations
	Cough	**3591**	3.83	3.70	3.96	1.83	1.78	1.88	Respiratory, thoracic and mediastinal disorders
	Pain	**3164**	1.09	1.05	1.13	0.12	0.07	0.17	General disorders and administration site conditions
	Oropharyngeal pain	**2019**	4.15	3.97	4.35	1.95	1.89	2.02	Respiratory, thoracic and mediastinal disorders
	Malaise	**1940**	1.47	1.41	1.54	0.54	0.47	0.60	General disorders and administration site conditions
	Respiratory tract congestion	**1572**	10.90	**10.32**	11.51	3.18	**3.10**	3.26	Respiratory, thoracic and mediastinal disorders
	Rhinorrhoea	**1330**	4.94	4.67	5.23	2.19	2.11	2.27	Respiratory, thoracic and mediastinal disorders
	Condition aggravated	**1026**	1.76	1.65	1.87	0.79	0.70	0.88	General disorders and administration site conditions

Feeling abnormal	**1012**	1.36	1.28	1.45	0.44	0.34	0.53	General disorders and administration site conditions
Nasopharyngitis	**791**	4.08	3.79	4.39	1.94	1.83	2.05	Infections and infestations
Symptom recurrence	280	16.25	**14.21**	18.59	3.65	**3.46**	3.84	General disorders and administration site conditions
Upper-airway cough syndrome	187	9.57	**8.19**	11.19	3.04	**2.81**	3.27	Respiratory, thoracic and mediastinal disorders
Acute kidney injury #	160	1.89	1.61	2.21	0.89	0.66	1.12	Renal and urinary disorders
Secretion discharge	137	6.83	5.71	8.16	2.62	**2.36**	2.88	General disorders and administration site conditions
Obstructive sleep apnoea syndrome	131	11.11	**9.20**	13.42	3.22	**2.95**	3.49	Respiratory, thoracic and mediastinal disorders
Chronic respiratory failure	94	10.50	**8.41**	13.11	3.15	**2.83**	3.47	Respiratory, thoracic and mediastinal disorders
Paranasal sinus hypersecretion	92	23.17	**18.12**	29.63	4.03	**3.68**	4.37	Respiratory, thoracic and mediastinal disorders
Pulmonary hypertension #	24	2.62	1.74	3.95	1.34	0.75	1.94	Respiratory, thoracic and mediastinal disorders
Pulmonary fibrosis #	18	2.10	1.31	3.36	1.04	0.36	1.71	Respiratory, thoracic and mediastinal disorders
Pulmonary septal thickening	6	51.59	**16.64**	159.96	4.72	**3.36**	6.07	Respiratory, thoracic and mediastinal disorders
Nasal mucosal discolouration	6	44.22	**14.86**	131.58	4.60	**3.27**	5.94	Respiratory, thoracic and mediastinal disorders
Idiopathic partial epilepsy	3	30.95	**7.40**	129.52	4.30	**2.57**	6.04	Nervous system disorders
Oropharyngeal discolouration	3	22.11	**5.72**	85.50	3.98	2.29	5.66	Respiratory, thoracic and

									mediastinal disorders
**All mRNA Vaccines**	Myocarditis	17669	5.22	**4.81**	5.66	0.18	**0.15**	0.21	Cardiac disorders
	Disease recurrence	3995	13.40	**10.19**	17.61	0.21	**0.15**	0.28	General disorders and administration site conditions
	Pericarditis	11735	4.18	**3.82**	4.58	0.17	**0.14**	0.21	Cardiac disorders
	Arrhythmia	16839	3.70	3.44	3.97	0.17	**0.13**	0.20	Cardiac disorders
	Heavy menstrual bleeding	15837	3.45	3.21	3.70	0.16	**0.13**	0.19	Reproductive system and breast disorders
	Dysmenorrhoea	8728	3.97	**3.58**	4.39	0.17	**0.13**	0.21	Reproductive system and breast disorders
	Menstruation delayed	4884	5.10	**4.37**	5.95	0.18	**0.13**	0.24	Reproductive system and breast disorders
	Lymphadenopathy	42408	2.63	2.53	2.73	0.14	**0.12**	0.16	Blood and lymphatic system disorders
	Breakthrough COVID-19	***227123***	2.08	2.05	2.12	0.11	**0.11**	0.12	Infections and infestations
	Palpitations	38260	2.34	2.25	2.43	0.13	0.11	0.15	Cardiac disorders
	Axillary pain	11010	2.88	2.66	3.11	0.15	**0.11**	0.19	General disorders and administration site conditions
	Mechanical urticaria	1191	7.16	**4.95**	10.35	0.20	0.08	0.31	Skin and subcutaneous tissue disorders
	Menometrorrhagia	832	13.18	**7.27**	23.90	0.21	0.07	0.35	Reproductive system and breast disorders
	Premenstrual syndrome	881	6.68	**4.41**	10.10	0.19	0.06	0.33	Reproductive system and breast disorders
	Fatigue	***195422***	1.37	1.35	1.38	0.06	0.05	0.07	General disorders and administration site conditions

Myalgia	***94653***	1.39	1.36	1.42	0.06	0.05	0.07	Musculoskeletal and connective tissue disorders
Dyspnoea	***89999***	1.43	1.40	1.45	0.06	0.05	0.08	Respiratory, thoracic and mediastinal disorders
Performance status decreased	972	5.13	**3.63**	7.26	0.18	0.05	0.31	General disorders and administration site conditions
Vaccination site lymphadenopathy	705	5.59	**3.65**	8.54	0.19	0.04	0.34	General disorders and administration site conditions
Headache	***218398***	1.16	1.14	1.17	0.03	0.02	0.04	Nervous system disorders
Arthralgia	***85622***	1.18	1.16	1.20	0.03	0.02	0.05	Musculoskeletal and connective tissue disorders
Anaphylactic reaction #	8975	1.38	1.30	1.47	0.06	0.02	0.10	Immune system disorders
Nausea	***118229***	1.12	1.10	1.14	0.02	0.01	0.03	Gastrointestinal disorders
Asthenia	***60776***	1.14	1.11	1.16	0.03	0.01	0.04	General disorders and administration site conditions
Acute kidney injury #	4067	1.51	1.37	1.66	0.07	0.01	0.14	Renal and urinary disorders
Dizziness	***117419***	1.07	1.05	1.08	0.01	0.00	0.02	Nervous system disorders
Malaise	***60638***	1.09	1.07	1.11	0.02	0.00	0.03	General disorders and administration site conditions

PT: Preferred Terms; n: Number of reports; ROR: reporting odds ratio; ROR_{025}: Lower limit of the 95% CI for ROR; ROR_{975}: Upper limit of the 95% CI for ROR; IC: Information component; IC025: Lower limit of the 95% credible interval for IC; IC975: Upper limit of the 95% credible interval for IC; SOC: System Organ Class. In PT column: ① # indicates PTs within designated medical events (DME) list; ② * indicates signals not listed in the product labeling. In other columns, values in bold indicate that the PT ranks among the top 10 highest values based on n, ROR_{025}, or IC−2SD.
