# Supplementary material for: Post-Marketing Safety of mRNA Vaccines: A Real-World Study Integrating Literature Case Reports and Vaccine Adverse Event Reporting System
Source: Vaccines (Basel). 2026 Jun 12;14(6):524. doi: 10.3390/vaccines14060524 (PMC13308135; doi:10.3390/vaccines14060524)
Supplement: Supplementary file 1 [file vaccines-14-00524-s001.zip › Table S9.pdf]

Table S9. Demographic characteristics of SAEs in VAERS.

SAEs	Characteristic	Comirnaty	Spikevax	mRESVIA	MNEXSPIKE	Comirnaty Bivalent	Spikevax Bivalent	Monovalent mRNA vaccines	Bivalent mRNA vaccines	All mRNA vaccines
**DIED**	Age (n (%))									
	Median	75	75	93	80	79	78	75	79	75
	< 6 months	9 (0.01)	NA	NA	NA	NA	NA	9 (0.01)	NA	9 (0.01)
	6 months-11 years	160 (0.19)	11 (0.03)	NA	NA	NA	1 (0.14)	171 (0.14)	1 (0.04)	172 (0.14)
	12-17 years	576 (0.69)	63 (0.15)	NA	NA	6 (0.36)	NA	639 (0.51)	6 (0.25)	645 (0.51)
	18-44 years	2838 (3.42)	2291 (5.51)	NA	NA	42 (2.53)	15 (2.14)	5129 (4.11)	57 (2.42)	5186 (4.08)
	45-64 years	9150 (11.01)	7220 (17.37)	NA	2 (5.88)	124 (7.48)	129 (18.43)	16372 (13.13)	253 (10.73)	16625 (13.09)
	≥65 years	39417 (47.44)	30866 (74.28)	10 (100.00)	32 (94.12)	1155 (69.66)	504 (72.00)	70325 (56.40)	1659 (70.36)	71984 (56.66)
	Unknown	30940 (37.24)	1105 (2.66)	NA	NA	331 (19.96)	51 (7.29)	32045 (25.70)	382 (16.20)	32427 (25.52)
	Sex (n (%))									
	F	36708 (44.18)	16940 (40.76)	10 (100.00)	12 (35.29)	717 (43.24)	291 (41.57)	53670 (43.04)	1008 (42.75)	54678 (43.04)
	M	44337 (53.36)	24176 (58.18)	NA	22 (64.71)	934 (56.33)	377 (53.86)	68535 (54.96)	1311 (55.60)	69846 (54.98)
	Unknown	2045 (2.46)	440 (1.06)	NA	NA	7 (0.42)	32 (4.57)	2485 (1.99)	39 (1.65)	2524 (1.99)
	History (n (%))									
	No history	1316 (1.58)	992 (2.39)	NA	2 (5.88)	79 (4.76)	12 (1.71)	2310 (1.85)	91 (3.86)	2401 (1.89)
	With history	29658	23015	10 (100.00)	27 (79.41)	800 (48.25)	471	52710	1271	53981

		(35.69)	(55.38)				(67.29)	(42.27)	(53.90)	(42.49)
	Unknown	52116 (62.72)	17549 (42.23)	NA	5 (14.71)	779 (46.98)	217 (31.00)	69670 (55.87)	996 (42.24)	70666 (55.62)
	Total	83090	41556	10	34	1658	700	124690	2358	127048
**L_THREAT**	Age (n (%))									
	Median	51	57	59	48	65	71	53	68	54
	< 6 months	19 (0.02)	18 (0.04)	NA	NA	NA	NA	37 (0.02)	NA	37 (0.02)
	6 months-11 years	532 (0.48)	71 (0.17)	NA	4 (3.12)	10 (0.46)	7 (0.65)	607 (0.40)	17 (0.52)	624 (0.40)
	12-17 years	2721 (2.46)	146 (0.35)	NA	NA	14 (0.64)	5 (0.46)	2867 (1.89)	19 (0.58)	2886 (1.86)
	18-44 years	20995 (18.99)	10893 (26.35)	NA	17 (13.28)	190 (8.70)	99 (9.15)	31905 (20.99)	289 (8.85)	32194 (20.73)
	45-64 years	20883 (18.89)	14849 (35.91)	14 (100.00)	63 (49.22)	487 (22.29)	222 (20.52)	35809 (23.55)	709 (21.70)	36518 (23.51)
	≥65 years	17500 (15.83)	14169 (34.27)	NA	41 (32.03)	857 (39.22)	712 (65.80)	31710 (20.86)	1569 (48.03)	33279 (21.43)
	Unknown	47893 (43.33)	1199 (2.90)	NA	3 (2.34)	627 (28.70)	37 (3.42)	49095 (32.29)	664 (20.32)	49759 (32.04)
	Sex (n (%))									
	F	62436 (56.48)	22321 (53.99)	14 (100.00)	81 (63.28)	1195 (54.69)	474 (43.81)	84852 (55.81)	1669 (51.09)	86521 (55.71)
	M	46775 (42.31)	18566 (44.91)	NA	45 (35.16)	977 (44.71)	599 (55.36)	65386 (43.01)	1576 (48.24)	66962 (43.12)
	Unknown	1332 (1.20)	458 (1.11)	NA	2 (1.56)	13 (0.59)	9 (0.83)	1792 (1.18)	22 (0.67)	1814 (1.17)
	History (n (%))									
	No history	7361 (6.66)	5311 (12.85)	NA	17 (13.28)	177 (8.10)	126 (11.65)	12689 (8.35)	303 (9.27)	12992 (8.37)

	With history	26691 (24.15)	19618 (47.45)	14 (100.00)	105 (82.03)	1072 (49.06)	665 (61.46)	46428 (30.54)	1737 (53.17)	48165 (31.01)
	Unknown	76491 (69.20)	16416 (39.70)	NA	6 (4.69)	936 (42.84)	291 (26.89)	92913 (61.11)	1227 (37.56)	94140 (60.62)
	Total	110543	41345	14	128	2185	1082	152030	3267	155297
**HOSPITAL**	Age (n (%))									
	Median	57	63	59	62	74	74	59	74	60
	< 6 months	69 (0.01)	23 (0.01)	NA	NA	NA	NA	92 (0.01)	NA	92 (0.01)
	6 months-11 years	3555 (0.58)	410 (0.18)	NA	12 (5.06)	126 (0.85)	60 (0.93)	3977 (0.47)	186 (0.87)	4163 (0.48)
	12-17 years	17042 (2.79)	1128 (0.49)	NA	NA	71 (0.48)	48 (0.74)	18170 (2.16)	119 (0.56)	18289 (2.12)
	18-44 years	93033 (15.24)	52469 (22.75)	3 (13.04)	51 (21.52)	622 (4.17)	416 (6.45)	145556 (17.30)	1038 (4.86)	146594 (16.99)
	45-64 years	98949 (16.21)	64402 (27.93)	14 (60.87)	71 (29.96)	2522 (16.92)	1123 (17.41)	163436 (19.43)	3645 (17.07)	167081 (19.37)
	≥65 years	136932 (22.43)	104974 (45.52)	6 (26.09)	102 (43.04)	9766 (65.51)	4682 (72.58)	242014 (28.77)	14448 (67.64)	256462 (29.73)
	Unknown	260910 (42.74)	7192 (3.12)	NA	1 (0.42)	1801 (12.08)	122 (1.89)	268103 (31.87)	1923 (9.00)	270026 (31.30)
	Sex (n (%))									
	F	344174 (56.38)	122248 (53.01)	17 (73.91)	136 (57.38)	7839 (52.58)	3145 (48.75)	466575 (55.46)	10984 (51.43)	477559 (55.36)
	M	259363 (42.48)	106561 (46.21)	6 (26.09)	101 (42.62)	7038 (47.21)	3261 (50.55)	366031 (43.51)	10299 (48.22)	376330 (43.62)
	Unknown	6953 (1.14)	1789 (0.78)	NA	NA	31 (0.21)	45 (0.70)	8742 (1.04)	76 (0.36)	8818 (1.02)

	History (n (%))									
	No history	17601 (2.88)	11617 (5.04)	NA	45 (18.99)	605 (4.06)	280 (4.34)	29263 (3.48)	885 (4.14)	30148 (3.49)
	With history	142935 (23.41)	94518 (40.99)	23 (100.00)	157 (66.24)	5787 (38.82)	2962 (45.92)	237633 (28.24)	8749 (40.96)	246382 (28.56)
	Unknown	449954 (73.70)	124463 (53.97)	NA	35 (14.77)	8516 (57.12)	3209 (49.74)	574452 (68.28)	11725 (54.89)	586177 (67.95)
	Total	610490	230598	23	237	14908	6451	841348	21359	862707
**X_STAY**	Age (n (%))									
	Median	48	58	/	67	56	83	50	74	50
	< 6 months	9 (0.11)	NA	NA	NA	NA	NA	9 (0.09)	NA	9 (0.09)
	6 months-11 years	168 (2.10)	1 (0.06)	NA	NA	NA	NA	169 (1.76)	NA	169 (1.75)
	12-17 years	297 (3.71)	15 (0.93)	NA	NA	NA	12 (34.29)	312 (3.25)	12 (17.65)	324 (3.35)
	18-44 years	1368 (17.11)	495 (30.78)	NA	NA	NA	NA	1863 (19.39)	NA	1863 (19.26)
	45-64 years	926 (11.58)	487 (30.29)	NA	NA	13 (39.39)	NA	1413 (14.71)	13 (19.12)	1426 (14.74)
	≥65 years	1252 (15.66)	537 (33.40)	NA	1 (100.00)	9 (27.27)	23 (65.71)	1790 (18.63)	32 (47.06)	1822 (18.83)
	Unknown	3977 (49.73)	73 (4.54)	NA	NA	11 (33.33)	NA	4050 (42.16)	11 (16.18)	4061 (41.98)
	Sex (n (%))									
	F	4624 (57.82)	1019 (63.37)	NA	NA	8 (24.24)	NA	5643 (58.74)	8 (11.76)	5651 (58.41)
	M	3233 (40.43)	571 (35.51)	NA	1 (100.00)	25 (75.76)	33 (94.29)	3805 (39.61)	58 (85.29)	3863 (39.93)

	Unknown	140 (1.75)	18 (1.12)	NA	NA	NA	2 (5.71)	158 (1.64)	2 (2.94)	160 (1.65)
	History (n (%))									
	No history	107 (1.34)	59 (3.67)	NA	NA	9 (27.27)	8 (22.86)	166 (1.73)	17 (25.00)	183 (1.89)
	With history	1258 (15.73)	670 (41.67)	NA	NA	5 (15.15)	15 (42.86)	1928 (20.07)	20 (29.41)	1948 (20.14)
	Unknown	6632 (82.93)	879 (54.66)	NA	1 (100.00)	19 (57.58)	12 (34.29)	7512 (78.20)	31 (45.59)	7543 (77.97)
	Total	7997	1608	NA	1	33	35	9606	68	9674
**DISABLE**	Age (n (%))									
	Median	47	47	34	48	61	64	47	63	47
	< 6 months	34 (0.01)	27 (0.03)	NA	NA	NA	NA	61 (0.02)	NA	61 (0.02)
	6 months-11 years	493 (0.19)	110 (0.13)	NA	4 (2.17)	6 (0.19)	NA	607 (0.18)	6 (0.12)	613 (0.17)
	12-17 years	2644 (1.02)	353 (0.40)	NA	NA	39 (1.23)	1 (0.05)	2997 (0.87)	40 (0.78)	3037 (0.86)
	18-44 years	52141 (20.17)	36702 (41.84)	1 (100.00)	68 (36.96)	479 (15.11)	300 (15.29)	88912 (25.66)	779 (15.18)	89691 (25.51)
	45-64 years	46013 (17.80)	31207 (35.58)	NA	78 (42.39)	629 (19.84)	637 (32.47)	77298 (22.31)	1266 (24.66)	78564 (22.35)
	≥65 years	20021 (7.74)	15323 (17.47)	NA	34 (18.48)	770 (24.28)	872 (44.44)	35378 (10.21)	1642 (31.99)	37020 (10.53)
	Unknown	137211 (53.07)	3991 (4.55)	NA	NA	1248 (39.36)	152 (7.75)	141202 (40.76)	1400 (27.27)	142602 (40.56)
	Sex (n (%))									
	F	178228 (68.93)	59095 (67.37)	1 (100.00)	139 (75.54)	2145 (67.64)	1232 (62.79)	237463 (68.54)	3377 (65.79)	240840 (68.50)
	M	76546 (29.61)	27648 (31.52)	NA	45 (24.46)	941 (29.68)	662 (33.74)	104239 (30.09)	1603 (31.23)	105842 (30.10)

	Unknown	3783 (1.46)	970 (1.11)	NA	NA	85 (2.68)	68 (3.47)	4753 (1.37)	153 (2.98)	4906 (1.40)
	History (n (%))									
	No history	12613 (4.88)	9070 (10.34)	1 (100.00)	73 (39.67)	223 (7.03)	202 (10.30)	21757 (6.28)	425 (8.28)	22182 (6.31)
	With history	38421 (14.86)	25259 (28.80)	NA	106 (57.61)	1191 (37.56)	725 (36.95)	63786 (18.41)	1916 (37.33)	65702 (18.69)
	Unknown	207523 (80.26)	53384 (60.86)	NA	5 (2.72)	1757 (55.41)	1035 (52.75)	260912 (75.31)	2792 (54.39)	263704 (75.00)
	Total	258557	87713	1	184	3171	1962	346455	5133	351588
**BIRTH_DEFECT**	Age (n (%))									
	Median	37	40	76	48	33	48	38	37	38
	< 6 months	2 (0.08)	12 (1.10)	NA	NA	NA	NA	14 (0.38)	NA	14 (0.37)
	6 months-11 years	52 (2.02)	1 (0.09)	NA	NA	NA	NA	53 (1.43)	NA	53 (1.41)
	12-17 years	39 (1.52)	NA	NA	NA	NA	NA	39 (1.05)	NA	39 (1.04)
	18-44 years	867 (33.70)	570 (52.49)	NA	NA	26 (76.47)	NA	1437 (38.80)	26 (57.78)	1463 (39.02)
	45-64 years	260 (10.10)	227 (20.90)	NA	39 (100.00)	6 (17.65)	11 (100.00)	526 (14.20)	17 (37.78)	543 (14.48)
	≥65 years	138 (5.36)	172 (15.84)	6 (100.00)	NA	NA	NA	316 (8.53)	NA	316 (8.43)
	Unknown	1215 (47.22)	104 (9.58)	NA	NA	2 (5.88)	NA	1319 (35.61)	2 (4.44)	1321 (35.24)
	Sex (n (%))									
	F	1590 (61.80)	806 (74.22)	6 (100.00)	39 (100.00)	32 (94.12)	6 (54.55)	2441 (65.90)	38 (84.44)	2479 (66.12)
	M	604 (23.47)	253	NA	NA	NA	5 (45.45)	857 (23.14)	5 (11.11)	862 (22.99)

			(23.30)							
	Unknown	379 (14.73)	27 (2.49)	NA	NA	2 (5.88)	NA	406 (10.96)	2 (4.44)	408 (10.88)
	History (n (%))									
	No history	342 (13.29)	186 (17.13)	NA	NA	11 (32.35)	NA	528 (14.25)	11 (24.44)	539 (14.38)
	With history	617 (23.98)	410 (37.75)	6 (100.00)	39 (100.00)	15 (44.12)	7 (63.64)	1072 (28.94)	22 (48.89)	1094 (29.18)
	Unknown	1614 (62.73)	490 (45.12)	NA	NA	8 (23.53)	4 (36.36)	2104 (56.80)	12 (26.67)	2116 (56.44)
	Total	2573	1086	6	39	34	11	3704	45	3749
**All SAEs**	Age (n (%))									
	Median	55	59	70	60	72	72	56	72	57
	< 6 months	100 (0.01)	63 (0.02)	NA	NA	NA	NA	163 (0.01)	NA	163 (0.01)
	6 months-11 years	4204 (0.47)	472 (0.14)	NA	12 (3.38)	132 (0.73)	60 (0.71)	4688 (0.38)	192 (0.72)	4880 (0.39)
	12-17 years	19679 (2.19)	1534 (0.46)	NA	NA	100 (0.55)	60 (0.71)	21213 (1.72)	160 (0.60)	21373 (1.69)
	18-44 years	145808 (16.20)	89642 (26.83)	4 (10.00)	80 (22.54)	1078 (5.97)	684 (8.10)	235534 (19.08)	1762 (6.65)	237296 (18.82)
	45-64 years	146502 (16.28)	97955 (29.32)	14 (35.00)	112 (31.55)	3039 (16.84)	1743 (20.63)	244583 (19.81)	4782 (18.05)	249365 (19.77)
	≥65 years	170303 (18.92)	132212 (39.58)	22 (55.00)	148 (41.69)	10571 (58.57)	5596 (66.25)	302685 (24.52)	16167 (61.02)	318852 (25.28)
	Unknown	413515 (45.94)	12176 (3.64)	NA	3 (0.85)	3129 (17.34)	304 (3.60)	425694 (34.48)	3433 (12.96)	429127 (34.03)
	Sex (n (%))									
	F	537071	187325	34 (85.00)	196 (55.21)	9775	4379	724626	14154	738780

	(59.67)	(56.08)			(54.16)	(51.84)	(58.70)	(53.42)	(58.58)
M	350181 (38.90)	143458 (42.94)	6 (15.00)	157 (44.23)	8158 (45.20)	3924 (46.45)	493802 (40.00)	12082 (45.60)	505884 (40.12)
Unknown	12859 (1.43)	3271 (0.98)	NA	2 (0.56)	116 (0.64)	144 (1.70)	16132 (1.31)	260 (0.98)	16392 (1.30)
History (n (%))									
No history	28438 (3.16)	19910 (5.96)	1 (2.50)	84 (23.66)	729 (4.04)	454 (5.37)	48433 (3.92)	1183 (4.46)	49616 (3.93)
With history	187648 (20.85)	124923 (37.40)	39 (97.50)	223 (62.82)	6894 (38.20)	3770 (44.63)	312833 (25.34)	10664 (40.25)	323497 (25.65)
Unknown	684025 (75.99)	189221 (56.64)	NA	48 (13.52)	10426 (57.76)	4223 (49.99)	873294 (70.74)	14649 (55.29)	887943 (70.41)
Total	900111	334054	40	355	18049	8447	1234560	26496	1261056

DIED: died; L_THREAT: life threatening; HOSPITAL: hospitalized; X_STAY: prolonged hospitalization; DISABLE: disability; BIRTH_DEFECT: Congenital anomaly or birth defect.
